# Restoring Tumor Cell Immunogenicity Through Ion‐Assisted *p53* mRNA Domestication for Enhanced In Situ Cancer Vaccination Effect

**DOI:** 10.1002/advs.202500825

**Published:** 2025-02-18

**Authors:** Yan Liang, Jingge Zhang, Jinjin Wang, Yuhe Yang, Xinyu Tan, Shuguang Li, Zhenzhen Guo, Zhenzhong Zhang, Junjie Liu, Jinjin Shi, Kaixiang Zhang

**Affiliations:** ^1^ School of Pharmaceutical Sciences Tianjian Laboratory of Advanced Biomedical Sciences Zhengzhou University Zhengzhou 450001 P. R. China; ^2^ Beijing Life Science Academy Beijing 102209 P. R. China

**Keywords:** cancer immune, in situ cancer vaccine, mRNA delivery, *p53*, tumor cell immunogenicity

## Abstract

The efficacy of in situ cancer vaccines (ISCVs) is hindered by the poor immunogenicity of tumor cells. Here, PRIZE, a P53‐repair nanosystem based on a virus‐mimicking nanostructure to deliver *p53* mRNA and Zn (II) into tumor cells, domesticating tumor cells by restoring intracellular P53 levels to bolster their immunogenicity, is designed. PRIZE ensures precise delivery to tumor sites, stabilizes *p53* mRNA with its biomineralized structure, and extends the half‐life of P53. This research highlights that PRIZE can efficiently repair P53 abnormalities in 4T1 (P53‐deficient) and MC38 (P53‐mutant) cells, subsequently upregulating the expression of major histocompatibility complex (MHC) class I molecules and the surface co‐stimulatory molecule CD80 on tumor cells, enhancing antigen presentation and transforming tumor cells into in situ antigen reservoirs. The co‐delivered photothermal agent (ICG) can trigger immunogenic cell death under laser irradiation, effectively releasing tumor‐associated antigens, and inducing the formation of ISCVs. Importantly, in P53 abnormal tumor mouse models, the induced ISCVs initiate the cancer immune cycle (CIC), demonstrating outstanding tumoricidal immunity and effectively thwarting tumor metastasis and postoperative recurrence, which provides valuable insights for advancing personalized cancer immunotherapy.

## Graphical abstract

PRIZE, a P53‐repair nanosystem based on a virus‐mimicking nanostructure, delivers *p53* mRNA into tumor cells, restoring P53 levels and enhancing immunogenicity. Its biomineralized structure stabilizes the mRNA, prolongs P53's half‐life, and precisely targets tumors. PRIZE also upregulates MHC I antigen presentation efficiency, induces immunogenic cell death with NIR irradiation, and activates the cancer immune cycle (CIC) by releasing tumor‐associated antigens.

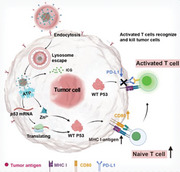

## 1 Introduction

ISCVs convert autologous tumor antigens into vaccines, offering a promising approach for personalized immunotherapy against diverse tumor types. ISCVs bypass the laborious tasks of identifying, synthesizing, and delivering specific antigens associated with traditional vaccines,^[^ ^1^ ^]^ thereby facilitating rapid translation to address pressing clinical demands.^[^ ^2^ ^]^ While other inoculation strategies such as photothermal therapy (PTT), radiotherapy, and in situ chemotherapy can be used to develop ISCVs,^[^ ^3^ ^]^ tumor cells possess the ability to cleverly evade systemic immune surveillance by reducing their immunogenicity, which limits the clinical application of ISCVs.^[^ ^4^ ^]^ Currently, some studies focus on enhancing the efficacy of ISCVs by accelerating the efficient recognition and clearance of tumor antigens by specific immune cells.^[^ ^5^ ^]^ However, the engineering modification of immune cells introduces challenges related to complexity, safety, and production costs.^[^ ^6^ ^]^ Hence, focusing on the early stage of tumor antigen release, enhancing the immunogenicity of tumor cells by “domesticating” them and transforming them into an antigen reservoir rich in abundant and high‐quality in situ antigens is a promising strategy.

At present, studies have shown that enhancing the immunogenicity of tumor cells can be achieved by blocking the surface exposure of phosphatidylserine on tumor cells or reducing the expression of the CD47 molecule on the surface of tumor cells.^[^ ^7^ ^]^ However, tumors are complex ecosystems, and cancer cells employ various strategies to evade immune attacks. Simply inhibiting or blocking a single factor may trigger compensatory mechanisms in the tumor, resulting in suboptimal immunogenic activation.^[^ ^8^ ^]^ Recent studies have shown that the tumor suppressor protein P53 through promotes apoptosis, regulates the major histocompatibility complex (MHC) class I pathway, and influences the expression of the costimulatory molecule CD80 to exert profound effects on the immunogenicity of cancer cells.^[^ ^9^ ^]^ However, ≈50% of human cancers exhibit *p53* abnormality, with wild‐type P53 (WT P53) levels being extremely low, leading to loss of its tumor‐suppressive function, reduced recruitment of anti‐cancer immune cells, and promoting immune evasion, thereby facilitating cancer cell proliferation, invasion, and metastasis.^[^ ^9^, ^10^ ^]^ Thus, repairing P53 abnormalities in tumor cells to boost their immunogenicity and promote immune cell infiltration may be critical for improving the efficacy of ISCVs and advancing their clinical translation.^[^ ^11^ ^]^

Considering the challenges in P53's undruggable reputation and the short half‐life of WT P53, we opt for delivering *p53* mRNA to restore the P53 level in tumor cells.^[^ ^12^ ^]^ The strategy of delivering *p53* mRNA circumvents the risk of gene integration by virus vectors and overcomes the ineffectiveness of small molecule compounds, which holds great promise for repairing P53 abnormalities.^[^ ^13^ ^]^ Yet, except for the challenge in delivery, maintaining the stability and functionality of the translated P53 protein still presents significant hurdles.^[^ ^14^ ^]^ Zn (II) has been shown to bind to the DNA‐binding domain (DBD) of WT P53 protein, maintaining its structural stability and promoting its transcriptional activation function.^[^ ^15^ ^]^ Besides, Zn (II) was reported to induce the generation of reactive oxygen species (ROS), leading to the degradation of mutant P53 protein (Mutp53) via ubiquitin‐mediated proteasomal pathways.^[^ ^16^ ^]^ However, current lipid nanoparticle (LNP)‐based delivery systems for *p53* mRNA are unable to concurrently transport metal ions.^[^ ^13^, ^17^ ^]^ Therefore, developing mRNA delivery vectors based on Zn (II) represents a promising *p53* mRNA delivery strategy for repairing P53 abnormalities.

**Scheme 1:**
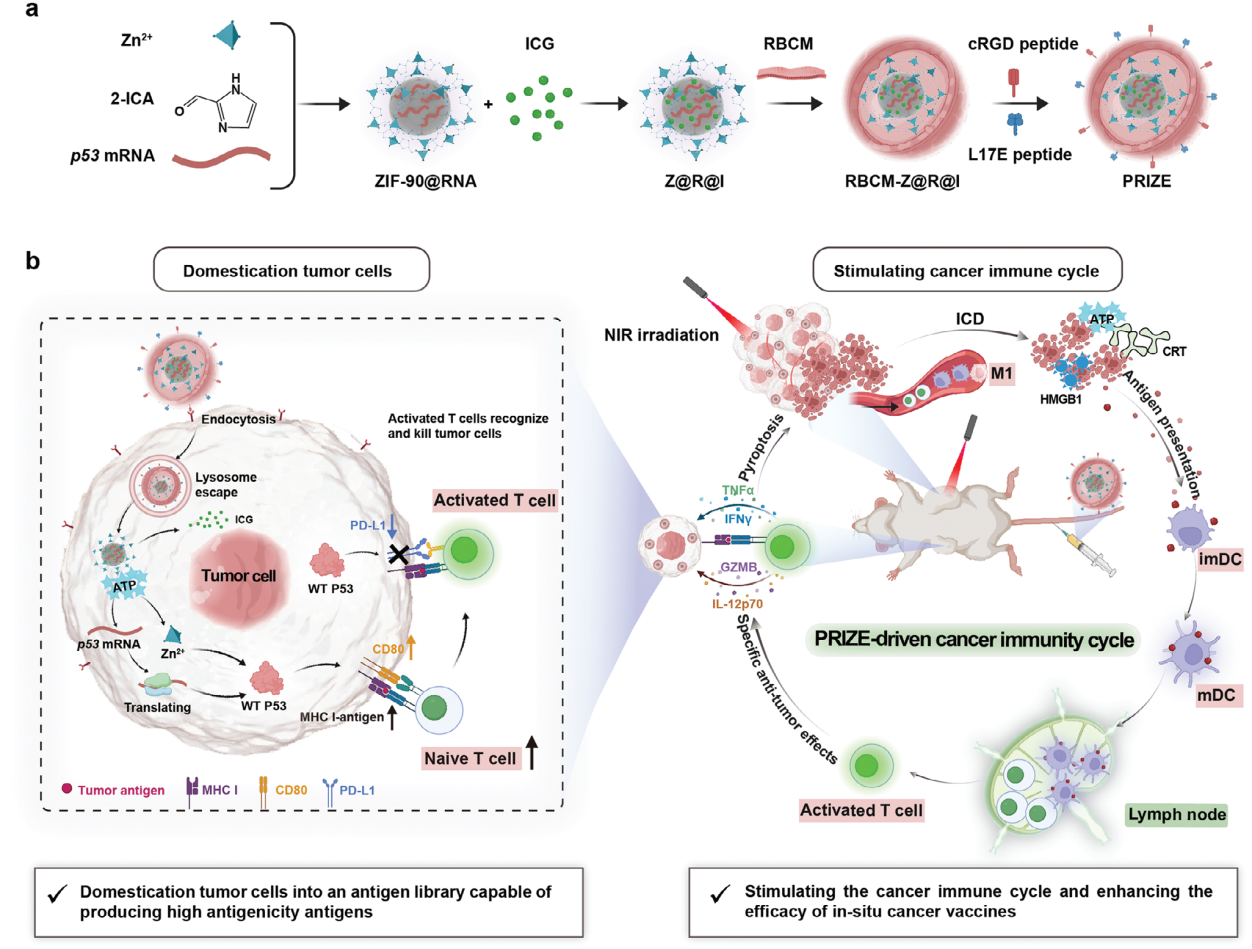
a) Synthesis Process of PRIZE. b) PRIZE enhanced the immunogenicity of domesticated tumor cells by targeting them and upregulating P53 expression, thereby facilitating effective antigen presentation. NIR irradiation efficiently induces ICD, leading to the release of tumor antigens, the formation of ISCVs, and the subsequent stimulation of DCs maturation and migration to LNs. This activation of systemic anti‐tumor immunity establishes a positive feedback loop that initiates CIC.

Here, we designed PRIZE, a P53‐repair nanosystem based on a virus‐mimicking nanostructure to deliver *p53* mRNA, aiming to domesticate tumor cells for enhanced efficacy of ISCVs. As shown in **Scheme** **1**, PRIZE delivered Zn (II) and *p53* mRNA to restore WT P53 levels in tumor cells and prolonged the half‐life of WT P53. Additionally, P53 can regulate multiple pathways in tumor cells, domesticating tumor cells into an in situ antigen reservoir while reducing tumor cell expression levels of PD‐L1, enhancing T cell recognition and increasing the killing efficiency against tumor cells. Next, PRIZE co‐delivered photothermal agent (ICG) into tumor cells, where it eradicated tumor cells under laser irradiation, triggering ICD efficiently and releasing tumor antigens, then inducing the formation of ISCVs and subsequently stimulating the maturation and migration to lymph nodes (LNs) of dendritic cells (DCs), activating systemic anti‐tumor immunity, establishing a positive feedback loop that initiates the cancer immune cycle (CIC).^[^ ^5^, ^18^ ^]^ In P53 abnormal tumor mouse models, PRIZE‐induced ISCVs activated CIC and generated robust immune memory. This strategy of enhancing the immunogenicity of tumor cells by repairing P53 abnormalities could present an attractive approach to advancing the clinical development of ISCVs.

## 2 Results and Discussion

### 2.1 Synthesis and Characterization of P53 Restoration Nano‐System

**Figure 1:**
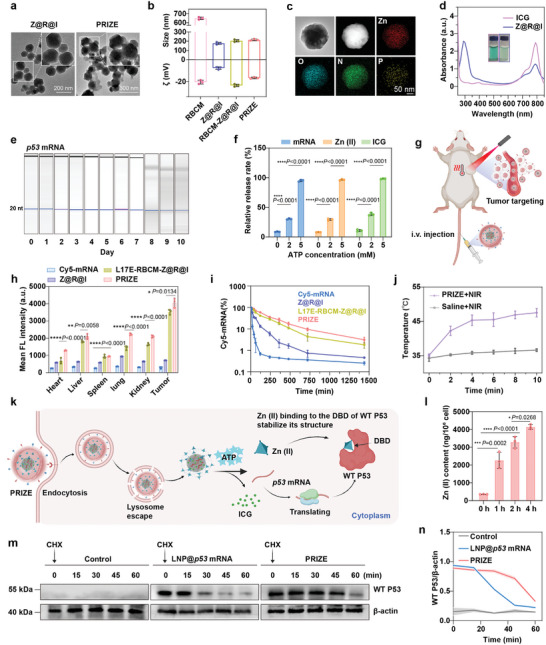
Synthesis and Characterization of P53 restoration nano‐system. a) Representative TEM image of Z@R@I and PRIZE. b) Size and zeta potentials (ζ) of RBCM, Z@R@I, RBCM‐Z@R@I and PRIZE (n = 3 independent samples). c) TEM mapping analysis of Z@R@I and corresponding element mapping images of Zn, O, N, P. Scale bar, 50 nm. d) UV–vis absorption spectra of free ICG and Z@R@I. e) Capillary electrophoresis analysis of *p53* mRNA stability in Z@R@I under 4 °C conditions. f) In vitro mRNA, Zn (II) and ICG release rate of Z@R@I at ATP concentrations of 0, 2, and 5 mM (n = 3 independent samples). g) Schematic diagram of PRIZE in vivo. h) The corresponding quantitative data showing the Cy5 fluorescence in different organs including tumors collected from mice injected via tail vein with various formulations with Cy5‐labeled *p53* mRNA and sacrificed at 30 h after the injections (n = 3 biologically independent samples). i) Circulation profile of mice injected via tail vein with various formulations with Cy5‐labeled *p53* mRNA. j) Temperature profiles of the mouse tumors after treatment with saline or PRIZE under persistent 2.0 W cm⁻^2^ laser irradiation (808 nm) (n = 3 biologically independent samples). k) The schematic illustration of PRIZE within tumor cell. l) ICP‐MS analysis of Zn (II) levels in 4T1 cells after PRIZE treatment (n = 3 biologically independent samples). m,n) Western blot analysis and quantitative assessment of WT P53 expression were performed at the indicated time points after 4T1 cells were treated with PRIZE or LNP@*p53* mRNA for 4 h, CHX was added to inhibit protein synthesis after 24 h of cultivation (n = 3 biologically independent samples). Data are presented as mean ± SD. Statistical analyses were done using one‐way analysis of variance (ANOVA) with Tukey's multiple comparisons test and correction. *P <0.05, **P <0.01, ***P <0.001, ****P <0.0001, ns, not significant.

To effectively repair P53 abnormalities, we designed a P53 restoration nano‐system based on a virus‐mimicking nanostructure termed PRIZE, with a schematic synthesis illustrated in Scheme 1a. The core consists of ZIF‐90 nanoparticles loaded with *p53* mRNA and ICG, termed Z@R@I, mimicking the structure of viral nucleocapsids. The outer layer is enveloped by red blood cell membranes (RBCM) modified with tumor‐targeting peptide cyclic RGDfK (cRGD) and lysosome‐escaping peptide L17E,^[^ ^19^ ^]^ imitating viral envelopes. Transmission electron microscopy (TEM) and dynamic light scattering (DLS) analysis revealed that the average size of the Z@R@I core was 177.6 ± 14.7 nm, with a zeta potential of ‐6.5 ± 1.9 mV, exhibiting a rhombic dodecahedral structural morphology identical to ZIF‐90 (**Figure** **1**a,b; Figure S1a, Supporting Information).^[^ ^20^ ^]^ TEM mapping analysis confirmed the distribution of N, O, Zn, and P elements in Z@R@I, validating the effective mRNA loading (Figure 1c). When the input amount of *p53* mRNA was 10 µg (500 µL synthesis system), the mRNA encapsulation efficiency of ZIF‐90@*p53* mRNA was 70.8% (Figure S1b, Supporting Information). This may be attributed to the efficient loading of mRNA by ZIF‐90 through π‐π stacking interactions between the aromatic rings of the imidazole linkers in ZIF‐90 and the aromatic rings of the nucleotide bases. UV‐visible spectra at 784 nm confirmed the successful encapsulation of ICG, suggesting the successful fabrication of Z@R@I (Figure 1d). Due to the inherent mRNA degradation propensity, we investigated the stability of *p53* mRNA encapsulated within nanoparticles. Using capillary electrophoresis to assess the integrity of *p53* mRNA stored in Z@R@I at 4 °C for 10 days. Results demonstrated that ZIF‐90 extended the stability of *p53* mRNA to 7 days, highlighting its robust mRNA protective effect (Figure 1e). This may be attributed to the biomimetic mineralization function of ZIF‐90.^[^ ^21^ ^]^ To ensure effective drug release inside cells, we evaluated the ATP‐responsive characteristics of Z@R@I and the release efficiency of mRNA, Zn (II), and ICG. It is known that tumor cytoplasmic ATP concentration ranges from 1–10 mM, and ATP competes with 2‐ICA for coordination, leading to the disintegration of ZIF‐90 and triggering drug release.^[^ ^22^ ^]^ Under the action of 5 mM ATP, the nanoparticles underwent complete disintegration instantaneously, with a release rate of 96% for Cy5‐labeled mRNA, a release rate of 97.5% for Zn (II) measured by inductively coupled plasma mass spectrometry (ICP‐MS) and a release rate of 98% for ICG measured by UV–vis spectra, confirming that the nanoparticles can respond to ATP and release drugs (Figure 1f; Movie S1, Supporting Information).

Subsequently, RBCM was coated onto the surface of Z@R@I, forming RBCM‐Z@R@I. After coextrusion, the particle size of RBCM‐Z@R@I increased to 203.6 ± 15.2 nm, and the zeta potential significantly decreased to ‐23.4 ± 2.3 mV, which demonstrated the successful encapsulation of RBCM (Figure 1b). Then the cRGD and L17E peptides were incorporated through a lipid‐insertion method, forming the PRIZE. PRIZE reached a particle size of 212.6 ± 12.6 nm, with a zeta potential of ‐16.8 mV ± 0.9 mV, confirming successful cRGD and L17E peptide modification (Figure 1b) and TEM revealed that PRIZE has the evident membrane structure (Figure 1a). Fluorescence quantitative analysis of FITC‐labeled cRGD peptide and Cy5‐labeled L17E peptide indicated modification efficiencies of 26.58% and 37.42%, respectively (Figure S2, Supporting Information). Furthermore, compared to Z@R@I, PRIZE exhibited consistent particle size, drug leakage rate, and polymer dispersity index (PDI) within 7 days at room temperature in PBS or 10% FBS, indicating superior physical stability (Figure S3, Supporting Information).

To explore the in vivo tumor‐targeting capability of PRIZE, we employed BALB/C mice and created 4T1 breast tumor models (Figure 1g). Prior to conducting targeted validation, hemolysis assays confirmed that PRIZE does not demonstrate significant blood incompatibility (Figure S4, Supporting Information). Once tumors reached ≈100 mm^3^ in size, various formulations containing Cy5‐*p53* mRNA (free Cy5‐*p53* mRNA, Z@R@I, L17E‐RBCM‐Z@R@I, and PRIZE) were intravenously administered at a consistent ZIF‐90 dose of 155 µg. Following injection, Cy5 fluorescence was monitored in the mice using whole animal imaging. Unlike free Cy5‐*p53* mRNA, Z@R@I, and L17E‐RBCM‐Z@R@I, PRIZE exhibited gradual accumulation at the tumor site, peaking in fluorescence intensity at 9 h post‐injection. Ex vivo fluorescence imaging demonstrated sustained retention of PRIZE in tumor tissue even up to 30 h after injection, highlighting the enhanced tumor‐targeting capability facilitated by RBCM and functional peptides (Figure 1h; Figure S5, Supporting Information). Meanwhile, thermal imaging experiments show that ICG can maintain its photothermal conversion capability for more than 24 h at tumor site (Figure S6, Supporting Information). Blood samples were collected at each time point to measure Cy5 fluorescence. While free Cy5‐*p53* mRNA and Z@R@I groups showed diminished fluorescence (decreased by one order of magnitude) after 8–12 h, L17E‐RBCM‐Z@R@I and PRIZE groups exhibited detectable fluorescence even at 24 h. This suggested that encapsulation of Cy5‐*p53* mRNA in NPs with RBCM prolonged its circulation time by protecting it from degradation (Figure 1i).

Then, the capability of PRIZE for converting near‐infrared (NIR) light to heat in vivo was evaluated by measuring the temperature alternation upon persistent NIR laser irradiation at an output power (808 nm, 2 W cm⁻^2^). The results demonstrated the excellent photothermal conversion capability of PRIZE and its potential to induce ICD (Figure 1j; Figure S7, Supporting Information).^[^ ^23^ ^]^

### 2.2 PRIZE Effectively Repaired P53 Abnormality through the Co‐Delivery of *p53* mRNA and Zn (II)

First, the uptake of nanoparticles (containing Cy5‐*p53* mRNA) by 4T1 (*p53*‐deficient) and MC38 (*p53*‐mutant) cells was analyzed via flow cytometry. It was found that over 90% of tumor cells internalized the NPs within 4 h, surpassing the uptake efficiency of free mRNA. Particularly noteworthy was PRIZE, with its uptake efficiency exceeding that of L17E‐RBCM‐Z@R@I, attributable to the enhanced specific absorption facilitated by cRGD peptide (Figure S8a,b, Supporting Information). In contrast, only ≈5.5% of RAW264.7 cells were Cy5‐positive, indicating that PRIZE could effectively evade immune surveillance and avoid rapid clearance by the immune system (Figure S8c, Supporting Information). Additionally, the lysosomal escape experimental results demonstrating the effective promotion of PRIZE escape by L17E peptide (Figure S9, Supporting Information).

Next, to demonstrate the response of intracellular ATP decomposition and the release of Zn (II) and mRNA after the lysosomal escape, we evaluated the intracellular concentrations of Zn (II) and the expression levels of P53 protein (Figure 1k). ICP‐MS results quantified the increase in intracellular Zn (II) content from 500 ng/10^6^ cells to 4000 ng/10^6^ cells, indicating effective intracellular accumulation of free Zn (II) (Figure 1l). Then, to determine whether PRIZE could induce therapeutic P53 expression in *p53*‐null 4T1 cells, P53 expression was assessed after treatment with PRIZE compared to PIZE (PRIZE without *p53* mRNA), PZE (PRIZE without *p53* mRNA and ICG), *p53* mRNA. Western blot confirmed the successful restoration of WT P53 expression after treatment with PRIZE (Figure S10, Supporting Information).

Given that Zn (II) can stabilize WT P53, we investigated whether PRIZE could effectively stabilize WT P53 through the co‐delivery of Zn (II) (Figure 1k). Next, we determined the half‐life of WT P53 by the protein synthesis inhibitor cycloheximide (CHX) after treatment with PRIZE,^[^ ^24^ ^]^ while comparing the results with those obtained from LNP@*p53* mRNA (without Zn (II)). Results revealed that the half‐life of WT P53 in 4T1 cells treated with PRIZE was found to be over 50 min, the stability of WT P53 significantly increased compared to LNP@*p53* mRNA (Figure 1m,n). This highlighted the advantage of PRIZE by co‐delivering Zn (II) for the repair of *p53* abnormalities in tumor cells with *p53*‐deficient.

In tumor cells with *p53* mutations, Mutp53 not only fosters tumor proliferation and metastasis through gain‐of‐function mechanisms, but also exerts a dominant‐negative effect on WT P53.^[^ ^9^, ^25^ ^]^ Therefore, degrading Mutp53 is essential for unleashing WT P53's tumor‐suppressive function. Studies indicate that increased intracellular Zn (II) levels can induce endogenous ROS generation, facilitating Mutp53 degradation via the ubiquitination pathway. Moreover, Zn (II) can partially restore Mutp53 conformation to that of WT P53.^[^ ^15b^ ^]^ Our research utilized confocal laser scanning microscopy (CLSM) to verify the induction of ROS by Zn (II), and confirmed a significant increase in WT P53 expression in MC38 cells through western blot analysis (Figures S11 and S12, Supporting Information). These findings underscore the adaptability of PRIZE in rectifying P53 abnormalities in tumor cells that are either deficient in or have mutations in *p53*.

### 2.3 PRIZE Domesticated Tumor Cells to Restore Immunogenicity, Transforming Them into an In Situ Antigen Reservoir

**Figure 2:**
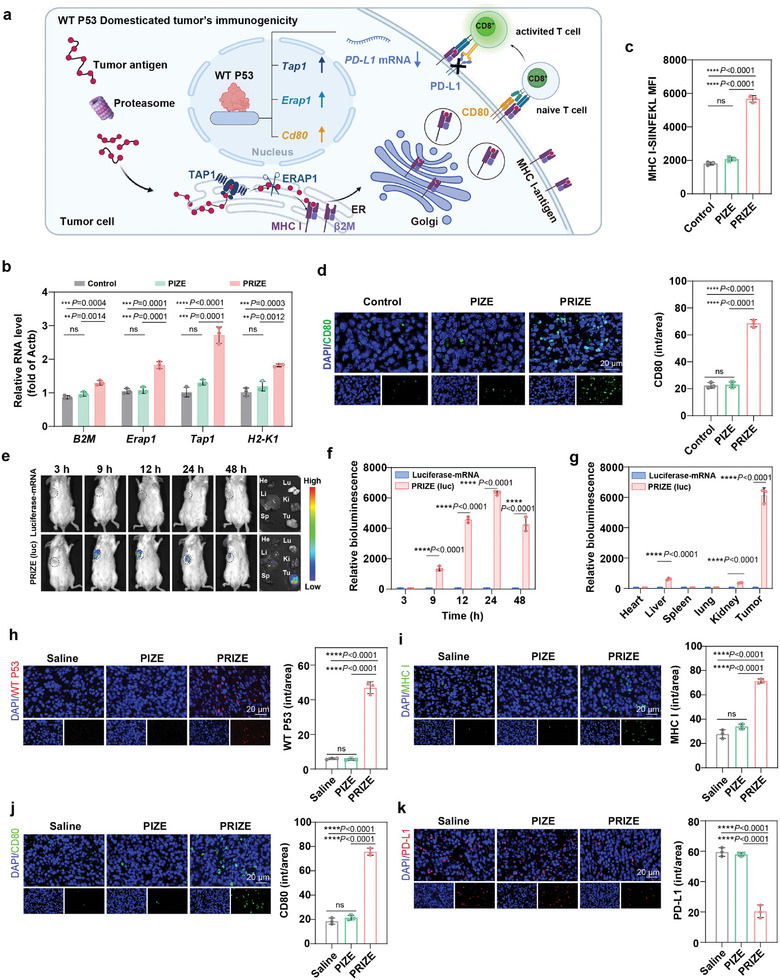
Restoring the immunogenicity of tumor cells through PRIZE domestication. a) The schematic illustration of WT P53 domesticated tumor cells into a highly immunogenic antigen repository. b,c) qRT‐PCR showed the induction of antigen presentation genes in 4T1 cells with different treatments. c) Mean fluorescence intensity (MFI) of the MHC‐SIINFEKL on the cell surface of 4T1‐OVA cells with different treatments assessed by flow cytometry (n = 3 biologically independent samples). d) The immunofluorescence images and quantitative analysis of immunofluorescence staining of cell surface CD80 in 4T1 cells post‐treatment. e) In vivo bioluminescence imaging after intravenous injection of PRIZE (Luc) in 4T1 tumor‐bearing mice at different time points and bioluminescence images of different organs collected from mice after the injections 48 h. f) The corresponding quantitative data showing the bioluminescence in 4T1 tumors at different time points (n = 3 biologically independent samples). g) The corresponding quantitative data showing the bioluminescence of different organs (n = 3 biologically independent samples). h–k) Representative images and quantitative analysis of immunofluorescence staining for WT P53, MHC I, CD80, PD‐L1 expression in 4T1 tumors after indicated treatments. Data are presented as mean ± SD. Statistical analyses were done using one‐way ANOVA with Tukey's multiple comparisons test and correction. *P <0.05, **P <0.01, ***P <0.001, ****P <0.0001, ns, not significant.

WT P53 directly contributes to the upregulation of antigen presentation genes through the MHC I pathway, including *B2M*, *Erap1*, *Tap1*, and *H2‐K1*.^[^ ^11^, ^26^, ^27^ ^]^ Besides, P53 as a direct regulator of the co‐stimulatory molecule CD80, when properly restored, could induce the expression of CD80 on the surface of tumor cells, enhancing their ability to present tumor antigens and thereby achieving effective immune activation (**Figure** **2**a).^[^ ^11^, ^28^ ^]^ To verify whether PRIZE has upregulated the expression of antigen presentation pathway‐related genes and CD80, we performed quantitative real‐time PCR (qRT‐PCR) and immunofluorescence staining analyses on tumor cells treated with PRIZE. Before that, we conducted a standard cell counting kit 8 assay to assess PRIZE cytotoxicity and determine the optimal dosing concentration at the cellular level. Consequently, a concentration of 30 µg mL⁻^1^ was chosen, which resulted in a cell viability of 74.7% (Figure S13, Supporting Information). qRT‐PCR results showed a significant upregulation of genes encoding MHC I molecules *B2M*, *H2‐K1*, *Tap1*, and *Erap1* in 4T1 cells after PRIZE treatment, compared with the PIZE and control groups (Figure 2b).^[^ ^29^ ^]^ This demonstrated that the upregulation of these genes is a benefit from *p53* mRNA within PRIZE. To verify the functional consequences of increased expression of antigen processing and presentation genes, we tested the presentation of heterologous MHC I‐bound peptide SIINFEKL, derived from chicken ovalbumin (OVA). Treatment with PRIZE successfully induced the cell surface presentation of OVA peptide in 4T1‐OVA cells, but not with PIZE and control groups (Figure 2c). Immunofluorescence staining demonstrated a significant increase in the surface expression of CD80 of 4T1 cells (Figure 2d). The significant elevation of MHC class I antigen presentation molecules and CD80 demonstrated that PRIZE enhances the immunogenicity of tumor cells through WT P53.

Inspired by our above results, we further evaluated whether PRIZE can domesticate tumor cells in vivo. Before that, we first evaluated the in vivo protein expression capability of PRIZE by intravenously injecting PRIZE (luc) loaded with firefly luciferase mRNA into 4T1 tumor‐bearing mice. The results revealed that compared to free luc‐mRNA, mice in the PRIZE (luc) group exhibited high luciferase expression at the tumor site and tumor tissue (Figure 2e–g). This indicated that VMN not only facilitates the precise targeting of mRNA to tumor sites, but also enables efficient protein expression. Next, we assessed the expression of P53 at the tumor site along with indicators of tumor immunogenicity, such as MHC I molecules and CD80. Saline, PIZE, and PRIZE were intravenously injected into different groups of 4T1 tumor‐bearing mice, with a nano‐formulation at an identical ZIF‐90 dose of 155 µg every 3 days for 5 times. Immunofluorescence results demonstrated that P53 was expressed at the highest levels in the PRIZE‐treated groups and we also observed a significant increase in MHC I molecules and CD80 expression in the PRIZE‐treated group, and a significant decrease in PD‐L1 expression compared to the other two groups (Figure 2h–k). The above results demonstrated that PRIZE effectively domesticated tumor immunogenicity in mice by rectifying P53 abnormalities.

The above results collectively demonstrated that PRIZE domesticates tumor cells abnormally by repairing P53, restored the immunogenicity of tumor cells, thereby transforming them into an in situ antigen reservoir, which established a robust foundation for effective T cell recognition and elimination of tumor cells.

### 2.4 Photothermal Triggered the Formation of ISCVs

**Figure 3:**
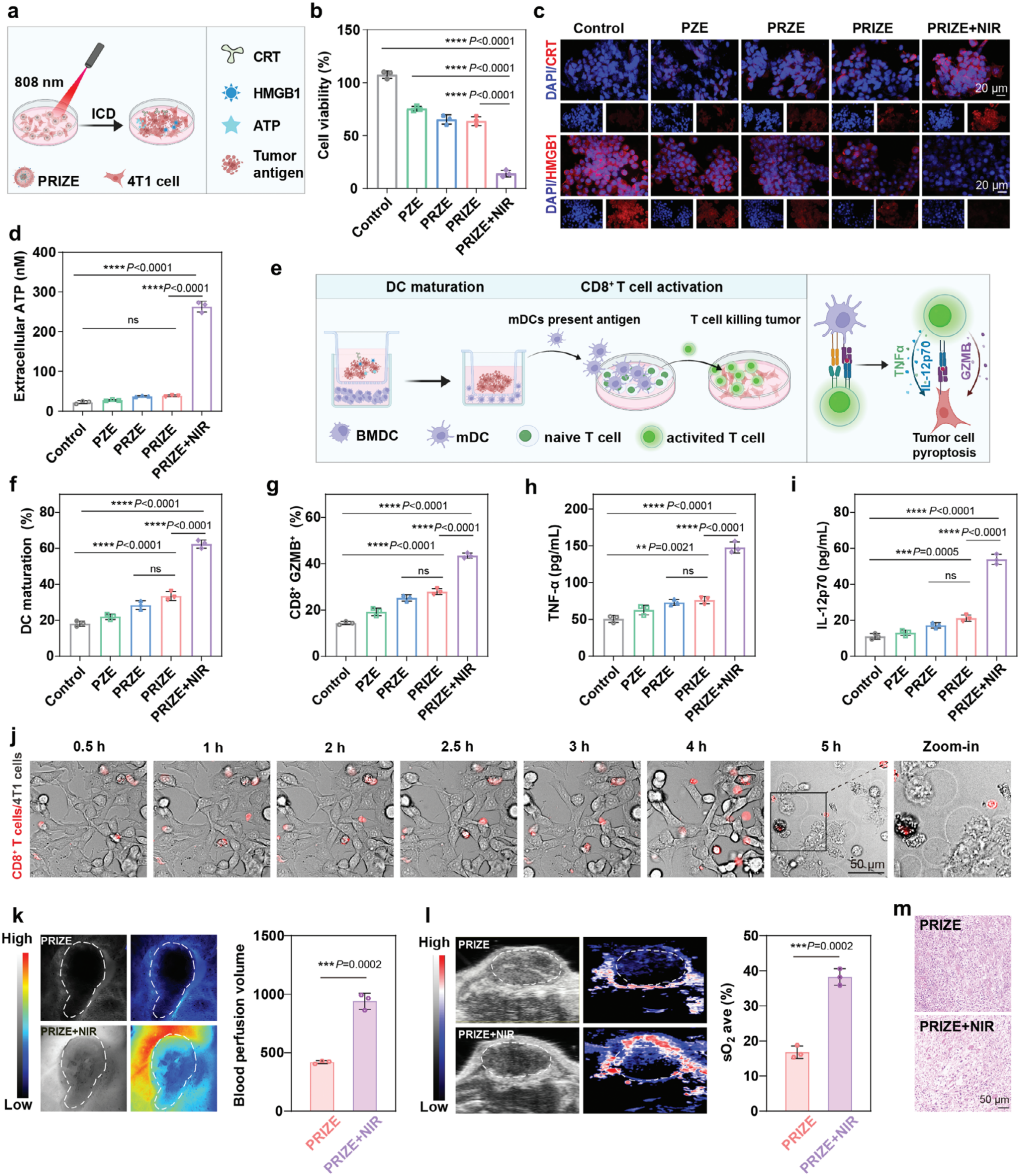
PRIZE+NIR triggered the formation of ISCVs. a) Schematic illustration of NIR‐accelerated ISCVs formation. b) Relative viability of 4T1 cells after 24 h treatment with PZE, PRZE, PRIZE, and PRIZE+NIR (43 °C, 5 min). c) Representative immunofluorescence images showing the expression of CRT and HMGB1 in 4T1 cells with different treatments. d) Extracellular ATP concentrations in 4T1 cells after different treatments (n = 3 biologically independent samples). e) Schematic illustration of the maturation of BMDCs and activation of CD8^+^ T cells. f) Quantitative flow cytometry data on maturing BMDCs after co‐culturing them with 4T1 cells for 24 h (n = 3 biologically independent samples). The 4T1 cells were pretreated with the indicated various formulations. g) Quantitative typical flow cytometry data of activated CD8^+^ T cells following co‐culture with the aforementioned BMDCs (n = 3 biologically independent samples). h,i) Production of h) TNF‐α and i) IL‐12p70 measured by ELISA after co‐culturing T cells with BMDCs (CD80^+^ CD86^+^) matured by 4T1 cells for 24 h (n = 3 biologically independent samples). The 4T1 cells were pretreated with PBS, PZE, PRZE, PRIZE, or PRIZE+NIR. j) Representative images showing the process of activated CD8^+^ T cells labeled with Cell Tracker™ DiI Dye attacking 4T1 cells. The T cells were activated by the BMDCs co‐cultured with 4T1 cells that received PRIZE+NIR treatment (n = 3 biologically independent samples). k) Blood perfusion images and quantitative changes of tumor sites in mice before and after NIR irradiation (n = 3 biologically independent samples). l) Representative PA imaging diagrams of 4T1 tumors before and after NIR. The quantification of average sO^2^ in tumors before and after NIR (n = 3 biologically independent samples). m) H&E staining of 4T1 tumors before and after NIR. Data are presented as mean ± SD. Statistical analyses were done using one‐way ANOVA with Tukey's multiple comparisons test and correction. *P <0.05, **P <0.01, ***P <0.001, ****P <0.0001, ns, not significant.

The advance in noninvasive phototherapy, including PTT and photodynamic therapy, provides opportunities for developing robust ISCVs (**Figure** **3**a).^[^ ^30^ ^]^ Benefit from co‐delivery of ICG with PRIZE to tumor cells, the cell survival rate was 13.6% after irradiated with NIR (43°C, 5 min), demonstrating excellent ICD efficacy (Figure 3b). ICD, associated with damage‐associated molecular patterns (DAMP) release, stimulates an antitumor immune response and triggers ISCVs formation.^[^ ^31^ ^]^ These DAMPs recruit immature DCs, aiding in their maturation when stimulated by dying tumor cells.^[^ ^32^ ^]^ In vitro experiments demonstrated that PRIZE+NIR significantly induced ICD in 4T1 cells and released DAMPs, such as calreticulin (CRT) exposure and high mobility group box 1 (HMGB1) and ATP release, underlining the significant impact of PTT on the ISCVs formation (Figure 3c,d). Subsequently, DCs maturation potential was further evaluated by collecting bone marrow cells from BALB/C mice and differentiating them into dendritic cells (BMDCs). It was found that co‐culturing BMDCs with PRIZE+NIR‐pretreated 4T1 cells increased DCs maturation prominently, further highlighting the importance of PTT in stimulating anti‐tumor immunity (Figure 3e, f; Figure S14a,b, Supporting Information).

Cytotoxic CD8^+^ T cells of the adaptive immune system are the most powerful effectors in the anticancer immune response and constitute the backbone of cancer immunotherapy.^[^ ^33^ ^]^ To investigate CD8^+^ T cell activation, we analyzed cytotoxic granzyme B (GZMB) expression using flow cytometry. Co‐culturing CD8^+^ T cells with BMDCs matured by PRIZE+NIR‐treated 4T1 cells significantly increased the percentage of CD8^+^ GZMB^+^ cells (43.3%), compared to others group (Figure 3g; Figure S14c,d, Supporting Information). Additionally, enzyme‐linked immunosorbent assay (ELISA) revealed a significant elevation of tumor necrosis factor‐α (TNF‐α) and interleukin 12 p70 (IL‐12p70) in the co‐culture system with PRIZE+NIR, indicating the activation of CD8^+^ T cell functionality (Figure 3h,i). Next, we assessed the tumor‐attacking capability of activated CD8^+^ T cells. CD8^+^ T cells activated by BMDCs matured with PRIZE+NIR‐treated 4T1 cells exhibited migration, aggregation, and sustained assault on tumor cells within 5 h, resulting in tumor cell eradication (Movie S2, Supporting Information). Morphological examination revealed a pyroptotic morphology in numerous cells in the PRIZE+NIR group, characterized by cell swelling and large bubble formation (Figure 3j).^[^ ^34^ ^]^ Therefore, PTT successfully triggered the release of antigens from PRIZE‐pretreated tumor cells, forming ISCVs and subsequently inducing an anti‐tumor immune response.

PTT may enhance immune cell infiltration by increasing the blood oxygen levels and facilitating the disruption of physical barriers in vivo.^[^ ^35^ ^]^ Not unexpectedly, laser blood flow imaging showed NIR irradiation increased the blood flow velocity of mouse, and photoacoustic imaging demonstrated elevated blood oxygen content in 4T1 tumor tissue (Figure 3k,l). Moreover, hematoxylin‐eosin staining (H&E) staining confirmed the expansion of loose regions within the tumor tissue after NIR treatment (Figure 3m), indicating the disruptive effect of PTT on physical barriers. These findings laid the foundation for the effective activation of the immune response and the achievement of potent anti‐tumor effects by PRIZE+NIR in vivo.

### 2.5 Therapeutic Efficacy of ISCVs in the Bilateral 4T1 Tumor Model

**Figure 4:**
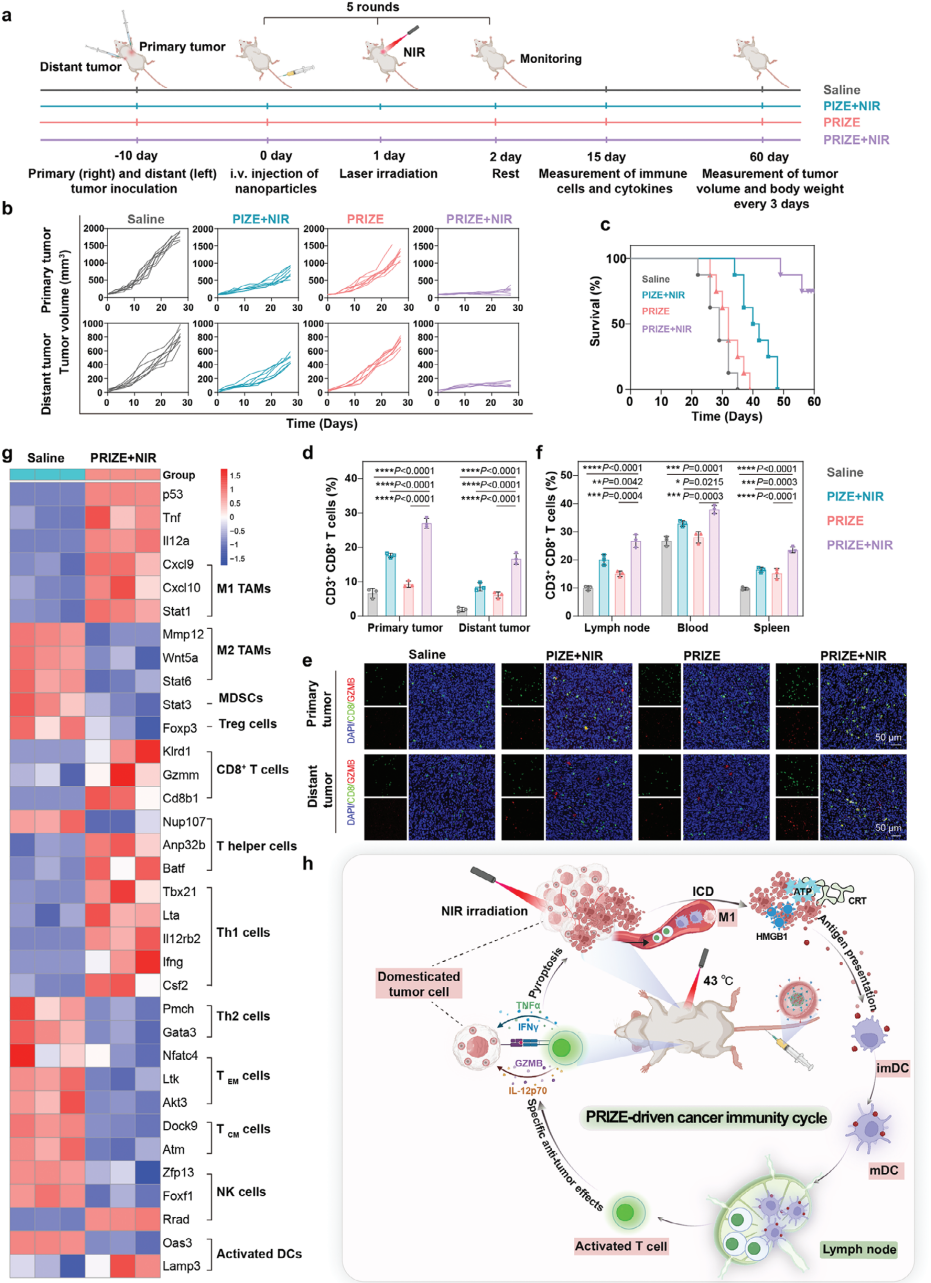
Therapeutic efficacy of ISCVs in the bilateral 4T1 tumor model. a) Schematic illustration of bilateral BALB/C tumor experimental design. For these mice, the tumors on the right and left sides are indicated as primary and distant tumors, respectively. b) Individual tumor growth kinetics of the primary and abscopal tumors are recorded every three days. c) Survival curve of each group (n = 8 biologically independent samples). d) Percentage of infiltrated CD3^+^ CD8^+^ T cells in primary tumor and distant tumor of mice in different groups (n = 3 biologically independent samples). e) Immunofluorescence analysis of CD8^+^ GZMB^+^ T cells in primary and distant tumors. f) Percentage of infiltrated CD3^+^ CD8^+^ T cells in the LNs, blood and spleen of mice in different groups (n = 3 biologically independent samples). g) Transcriptome analysis of genes associated with immune cell types in primary tumor tissues harvested from the mouse after treatment with saline, PRIZE+NIR. Differential gene cluster analysis was shown as a heat map (n = 3 biologically independent samples). h) Schematic illustration of PTT synergized with PRIZE domestication to activate CIC and enhance the efficacy of ISCV. Data are presented as mean ± SD. Statistical analyses were done using one‐way ANOVA with Tukey's multiple comparisons test and correction. *P <0.05, **P <0.01, ***P <0.001, ****P <0.0001, ns, not significant.

The antitumor efficacy of ISCVs was evaluated using a bilateral BALB/C tumor model. Mice were divided into four groups (n = 8 per group) once the primary tumor volume reached 100 mm^3^. They were then intravenously administered saline, PIZE+NIR, PRIZE, or PRIZE+NIR every three days, for a total of five injections. For the PIZE+NIR or PRIZE+NIR treated groups, an additional laser irradiation (43°C, 5 min) was applied to the right (primary) tumor 24 h post‐injection (**Figure** **4**a). Intravital experimental data revealed that PRIZE therapy or PIZE+NIR therapy individually suppressed the bilateral growth of 4T1 tumors compared to the saline‐treated control mice. However, the PRIZE+NIR treatment exhibited superior efficacy, evident from the growth curves of individual subjects and the average tumor volumes (Figure 4b; Figure S15, Supporting Information). Consequently, while mice in other groups succumbed within 60 days, ≈75% of those receiving combined treatment survived (Figure 4c). These findings underscored the significance of enhancing tumor cell immunogenicity through the repair of P53 abnormalities to improve the efficacy of ISCVs.

Furthermore, blood samples were collected for hematological and blood biochemical analyses. Liver and kidney function indices of mice treated with the nanoplatform remained within normal ranges, and hematological parameters showed no toxic effects of the nanoplatform (Figure S16a, Supporting Information). Additionally, H&E staining of vital organs revealed no apparent damage, confirming the safety of the PRIZE+NIR‐based antitumor therapy (Figure S16b, Supporting Information).

### 2.6 The ISCVs of PRIZE+NIR‐Based Established a Positive Feedback Loop to Continually Initiate the Cancer Immune Cycle

To gain a deeper understanding of the mechanism of PRIZE+NIR‐mediated therapy, immune responses were studied. PTT has the potential to trigger an ICD cascade, releasing TAA, DAMPs, and pro‐inflammatory cytokines, thereby generating an ISCVs effect.^[^ ^36^ ^]^ Consequently, we first confirmed that the expression of ICD markers HMGB1 and CRT was significantly elevated in the primary tumors of the NIR treatment group, indicating that NIR induces ICD in tumor cells, thereby initiating subsequent immune responses (Figure S17, Supporting Information). Then, utilizing flow cytometry, we assessed mature DCs in primary tumors and tumor‐draining LNs. As illustrated in Figures S18 and S19 (Supporting Information), NIR exposure fostered DC maturation in both tumor tissues and inguinal LNs, resulting in significantly elevated percentages of mature DCs (CD86^+^ CD80^+^) and MHC I^+^ DC across all NIR‐treated groups. Particularly, PRIZE+NIR treatment exhibited the highest DC maturation and antigen presentation rates among all treatments. Moreover, in NIR‐treated primary tumors, enhanced DC maturation facilitated the heightened accumulation of cytotoxic T lymphocytes (CTL), notably increasing the CD3^+^ CD8^+^ T cell percentages of primary and distant tumor by 20.36% and 14.70%, respectively, compared to saline groups (Figure 4d; Figure S20, Supporting Information). Importantly, the percentages of CD8^+^ GZMB^+^ T cells in primary or distant tumor significantly enhanced in PRIZE+NIR‐treated mice, predicting the effective tumor‐infiltrating CTL‐mediated immune responses that are favorable for tumor elimination (Figure 4e; Figure S21, Supporting Information).

To assess systemic anti‐tumor immune response, we conducted flow cytometry analysis of T cells in distant tumors, LNs, spleen, and peripheral blood. As shown in Figure 4f, Figure S22 (Supporting Information), the PRIZE+NIR treatment group elicited a higher proportion of CD3^+^ CD8^+^ T cells compared with PIZE+NIR group, demonstrating the effectiveness of PRIZE‐mediated tumor immunogenicity recovery in enhancing immune response. Encouraged by these findings, we measured the release of immune‐relevant cytokines within tumor tissues, such as IFN‐γ, TNF‐α, IL‐12p70, and IL‐10, using ELISA. The combination of PRIZE and NIR resulted in the highest levels of immune‐stimulating cytokines (IFN‐γ, TNF‐α, and IL‐12p70), accompanied by the lowest concentration of immunosuppressive cytokine (IL‐10) in tumors and serum (Figure S23, Supporting Information). Tumor cells domesticated via PRIZE displayed enhanced antigen presentation, making them more susceptible to recognition and elimination by CTLs. This, in conjunction with PTT, established a positive feedback loop that initiated the CIC.

Next, we sequenced RNA extracted from tumor tissues collected after treatment to further confirm the conclusion. Highly altered gene expression was apparent in the group treated with PRIZE+NIR (Figure S24a, Supporting Information). We observed that tumor tissues treated with PRIZE+NIR showed an upregulated expression of *p53* and many immune activation‐related genes as well as changes in immune cell types, including T cells, NK cells, and activated DCs (Figure 4g). Surprisingly, we found that the expression of genes related to tumor‐associated macrophages (TAMs) subtypes (M1 and M2), myeloid‐derived suppressor cells (MDSCs), and regulatory T cells (Tregs) had changed, and these changes might remodel the immunosuppressive tumor microenvironment (TME), further enhancing CIC. Moreover, analysis of apoptosis genes associated with P53 revealed its tumor‐suppressive role (Figure S24b, Supporting Information). Furthermore, Kyoto Encyclopedia of Genes and Genomes (KEGG) enrichment analysis identified differentially expressed gene (DEG) enrichment in multiple immune‐response‐associated signaling pathways (Figure S24c, Supporting Information). Flow cytometry was employed to analyze infiltrating immune cells in 4T1 primary tumors, focusing on TAMs, MDSCs, and Tregs. Our results, depicted in Figure S25 (Supporting Information), showed a significant decrease in MDSCs and Tregs proportions in the PRIZE+NIR group compared to saline group, along with an increase in the M1/M2 ratio, indicating the improvement effects of PRIZE+NIR on TME. In summary, in vivo results demonstrated that PRIZE+NIR effectively eliminated tumors by eliciting adaptive immune responses, and modulating TME, thus enhancing the efficacy of ISCVs. The remarkable tumor therapeutic effect demonstrated that our developed strategy of enhancing tumor cell immunogenicity through a *p53* mRNA delivery approach is a feasible method for improving the efficacy of ISCVs therapy (Figure 4h).

### 2.7 Inhibition of Tumor Metastasis and Tumor Recurrence by PRIZE+NIR

**Figure 5:**
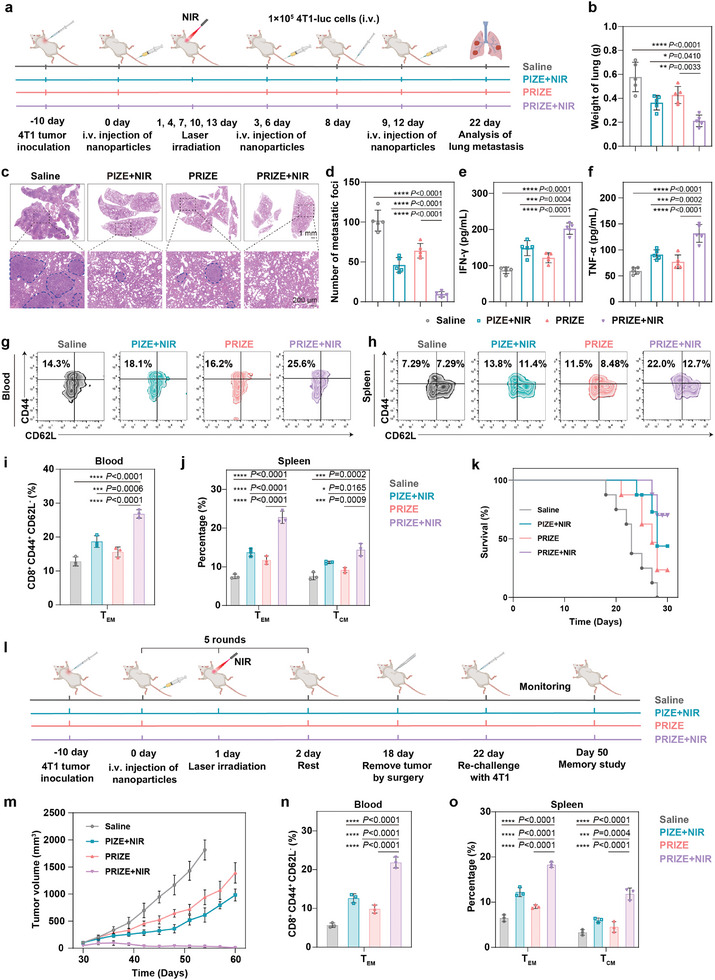
Prevention of tumor metastasis and long‐term memory effects in tumor recurrence. a) Schematic illustration of PRIZE+NIR‐triggered cancer immunotherapy in a 4T1 metastasis model. b) Weight of lungs collected from mice at the end of the study (n = 5 biologically independent samples). c) Representative H&E staining images of lung tissues collected at the end of the study. d) Quantification of lung metastasis nodes per mouse from mice at the end of the study (n = 5 biologically independent samples). e,f) Cytokine levels of IFN‐γ, and TNF‐α in blood serum after various treatments by ELISA assay (n = 5 biologically independent samples). g,h) Representative flow cytometry analysis of T_EM_ (CD8^+^ CD44^+^ CD62L^−^) in blood (g) and T_EM_ (CD8^+^ CD44^+^ CD62L^−^) and T_CM_ (CD8^+^ CD44^+^ CD62L^+^) in spleens (h) collected from mice of 4T1 metastasis model with the various treatments (n = 3 biologically independent samples). i,j) The percentage of T_EM_ (CD8^+^ CD44^+^ CD62L^−^) in blood and T_EM_ (CD8^+^ CD44^+^ CD62L^−^) and T_CM_ (CD8^+^ CD44^+^ CD62L^+^) in spleens collected from mice with the various treatments (n = 3 biologically independent samples). k) Overall survival rate of BALB/C mice with 4T1 lung metastasis in different groups (n = 8 biologically independent samples mice). l) Schematic illustration of treatment schedule for PRIZE+NIR‐mediated prevention of tumor recurrence in a 4T1 tumor model. m) Tumor growth curves. n) The percentage of effector memory T cells (T_EM_, CD8^+^ CD44^+^ CD62L^−^) in blood collected from mice with the various treatments (n = 3 biologically independent samples). o) The percentage of T_EM_ (CD8^+^ CD44^+^ CD62L^−^) and T_CM_ (CD8^+^ CD44^+^ CD62L^+^) in spleens collected from mice with the various treatments (n = 3 biologically independent samples). Data are presented as mean ± SD. Statistical analyses were done using one‐way ANOVA with Tukey's multiple comparisons test and correction. *P <0.05, **P <0.01, ***P <0.001, ****P <0.0001, ns, not significant.

Considering the robust immune responses and therapeutic effects achieved by PRIZE+NIR, we further assessed its potential in treating tumor metastasis and recurrence. 4T1 tumor‐bearing mice were randomly divided into four groups and treated with different formulations. On the 8th day, 1 × 10^5^ 4T1‐luc cells were intravenously injected into the mice to simulate tumor cell escape from a primary tumor. Pulmonary metastases were then detected until the demise of the mice or experimental termination (**Figure** **5**a). At the end of the study, PRIZE+NIR reduced the metastatic tumor burden in lungs, resulting in lung weights of 0.21 ± 0.03 g, close to normal lung weight among all treatment groups (Figure 5b).^[^ ^37^ ^]^ Also, the lung tissues in different groups were collected for metastasis analysis by H&E staining. As shown in Figure 5c,d and Figure S26 (Supporting Information), compared to the saline, PIZE+NIR, and PRIZE groups, the PRIZE+NIR group exhibited few pulmonary foci and normal lung morphology without swelling, with the number of metastatic nodules reduced by ≈60% compared to the saline group, indicating its potential in preventing metastatic tumors. Moreover, concentrations of IFN‐γ and TNF‐α in serum reached the highest values in PRIZE+NIR‐treated mice, indicating an optimized antitumor immune response (Figure 5e,f). We further utilized the IVIS Lumina II imaging system to monitor tumor metastasis. In contrast to saline‐treated mice with severe lung metastasis, no bioluminescence signal was detected in mice treated with PRIZE+NIR (Figure S27, Supporting Information). The stalwart inhibition of lung metastasis could be ascribed to the generation of the effector memory T cells (T_EM_, CD8^+^ CD44^+^ CD62L^−^) and the central memory T cells (T_CM_, CD8^+^ CD44^+^ CD62L^+^) in the spleens and T_EM_ in the peripheral blood after the treatment.^[^ ^38^ ^]^ PRIZE+NIR‐treated increased the frequency of T_EM_ cells in the peripheral blood of mice by 14.00% compared to the saline group (Figure 5g,i; Figure S28, Supporting Information). Additionally, in the PRIZE+NIR‐treated group, the proportions of T_EM_ and T_CM_ cells in the spleen were elevated by 15.25% and 6.82%, respectively, compared to the saline group (Figure 5h,j). As a result, the survival rate significantly improved to 70% for mice treated with PRIZE+NIR (Figure 5k). These findings supported the applicability of PRIZE+NIR in generating durable long‐term antitumor memory immune responses to inhibit tumor metastasis and prolong overall animal survival, highlighting the importance of restoring P53 levels in tumor cells for activating long‐lasting immune memory effect.

We further established a tumor rechallenge model to assess the enduring immune memory effect of PRIZE+NIR. BALB/C mice with 4T1 tumors received intravenous administration of saline, PIZE, or PRIZE. All tumors were surgically removed on the 18th day, after which mice were rechallenged with 4T1 tumor cells four days post‐treatment (Figure 5l). Results depicted in Figure 5m indicated complete regression of reinjected 4T1 tumors in mice treated with PRIZE+NIR. Moreover, PRIZE+NIR treatment led to peak levels of memory T cells in the peripheral blood and spleen, surpassing those of other groups, suggesting potent long‐term immune memory effects to prevent tumor recurrence (Figure 5n,o; Figure S29, Supporting Information). The above results indicated the effectiveness of leaving a well‐trained immune system before surgery to combat tumor recurrence.

### 2.8 The Antitumor Efficacy of PRIZE+NIR against the P53 Mutant MC38 Colon Cancer Tumor Model

**Figure 6:**
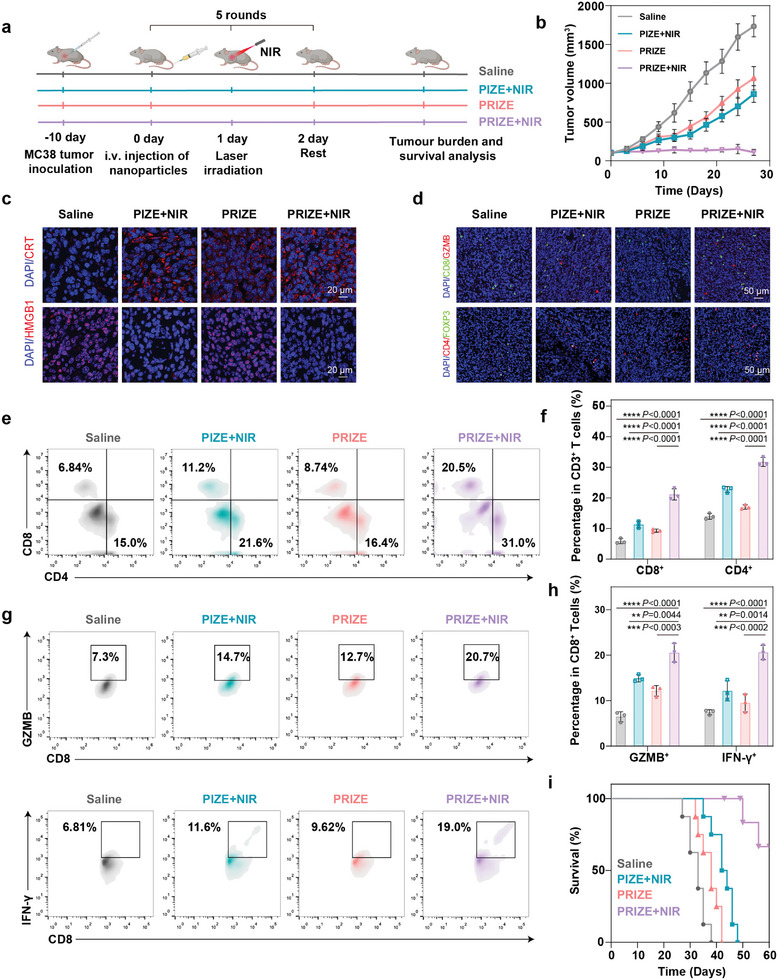
The antitumor effects of PRIZE+NIR in inhibiting P53 mutant tumors. a) Schematic illustration of PRIZE+NIR‐triggered cancer immunotherapy in a MC38 tumor model. b) Tumor growth curves of tumors (n = 8 biologically independent samples). c) Representative immunofluorescence images showing the expression of CRT, HMGB1 in tumors treated by the indicated formulations in combination with NIR. d) Immunofluorescence analysis of CD8^+^ GZMB^+^ T cells and CD4^+^ Foxp3^+^ T cells tumors of different groups. e,f) Flow cytometry and quantitative flow cytometry data of infiltrated CD3^+^ CD8^+^ T cells and CD3^+^ CD4^+^ T cells in tumors of mice in different groups (n = 3 biologically independent samples). g,h) Flow cytometry and quantification of expression levels of CD8^+^ GZMB^+^ T cells and CD8^+^ IFN‐γ^+^ T cells in tumors in different groups (n = 3 biologically independent samples). The representative gating strategies are provided in Figure S30 (Supporting Information). i) Overall survival rate of C57BL/6 mice with MC38 tumor in in different groups (n = 8 biologically independent samples). Data are presented as mean ± SD. Statistical analyses were done using one‐way ANOVA with Tukey's multiple comparisons test and correction. *P <0.05, **P <0.01, ***P <0.001, ****P <0.0001, ns, not significant.

Finally, we validated the therapeutic efficacy of PRIZE+NIR and its anti‐tumor immune response in a murine tumor model with P53 mutation (**Figure** **6**a). In C57BL/6 mice bearing MC38 tumor, PRIZE+NIR therapy exhibited superior efficacy in both restraining tumor growth and achieving complete tumor eradication (Figure 6b). We validated the induction of tumor cell ICD by NIR through immunofluorescence, demonstrating significant CRT externalization and HMGB1 translocation (Figure 6c). Post‐treatment, we evaluated the anti‐tumor immune response within the murine tumor model. Immunofluorescent images of CD8^+^ GZMB^+^ T cells and CD4^+^ Foxp3^+^ T cells revealed enhanced activation of CD8^+^ T cells after PRIZE+NIR intervention (Figure 6d). Flow cytometry analysis indicated a notable increase in T‐cell infiltration levels within tumor tissues following PRIZE+NIR treatment (Figure 6e,f). Furthermore, there was a significant rise in populations of CD8^+^ GZMB^+^ and CD8^+^ IFN‐γ^+^ T‐cells, suggesting that PRIZE+NIR enhanced the anti‐tumor activity of CD8^+^ T‐cells, eliciting robust immune responses (Figure 6g,h; Figure S30, Supporting Information). Importantly, survival curve analysis demonstrated that PRIZE+NIR treatment improved the survival rate of MC38 colon cancer mice to 60% within 60 days, highlighting its potent anti‐tumor efficacy (Figure 6i). The above results indicated that the proposed P53 repair strategy was applicable in tumor models with *p53* mutations.

## 3 Conclusion

Here, we designed PRIZE, a P53‐repair nanosystem based on a virus‐mimicking nanostructure, which efficiently delivers *p53* mRNA and Zn(II) to restore the immunogenicity of tumor cells and enhance the efficacy of ISCVs. PRIZE ensures the precise delivery of *p53* mRNA to tumor sites, stabilizes it with its biomineralized structure, and extends the half‐life of WT P53. Notably, our research demonstrates that PRIZE effectively corrects P53 abnormalities in tumor cells, domesticating them to improve antigen presentation and transforming them into an in situ antigen reservoir. Furthermore, in vivo experiments revealed that PRIZE facilitates the release of unique antigens under NIR laser irradiation, enabling in situ cancer vaccine inoculation. These findings highlight the critical role of restoring WT P53 levels in tumor cells to activate the CIC and achieve potent anti‐tumor effects. Our study not only introduces a novel strategy for repairing P53 abnormalities, but also underscores the importance of enhancing tumor cell immunogenicity in anti‐tumor therapy. Overall, this work offers a promising approach to accelerate the clinical translation of ISCVs without the need for personalized vaccines for each patient.

## 4 Experimental Section

### Materials

Imidazole‐2‐carboxyaldehyde and Zn (OAc)_2_·2H_2_O (Sigma, USA); *p53* mRNA (APExBIO, Houston, USA); DSPE‐PGE2000‐cRGD(fK) peptide [(DSPE‐PEG2000‐Mal)‐cyclic‐(Arg‐Gly‐Asp‐D‐Phe‐Lys)] was synthesized by Xi'an ruixi Biological Technology Co., Ltd. (Shaanxi, China); DSPE‐PGE2000‐L17E [(DSPE‐PEG2000‐Mal)‐CIWLTALKFLGKHAAKHEAKQQLSKL] was synthesized by ChinaPeptides Co., Ltd (Shanghai, China); Triphosphate (ATP) (New England Biolabs); Annexin V‐FITC Apoptosis Detection Kit (KeyGEN BioTECH); ATP Content Assay Kit (Beijing Solarbio Science & Technology Co., Ltd); Mouse cytokine ELISA kits for IL‐12p70 and IFN‐γ (Beijing Solarbio Science & Technology Co., Ltd); Mouse IL‐1β ELISA Kit (abs520001) (Absin (Shanghai) Biotechnology Co., Ltd); Mouse Tumor Necrosis Factor Alpha (TNFα) ELISA Kit (JL10484, Jianglai biology, Shanghai); Indocyanine Green (ICG) and Cycloheximide (CHX) (MedChemExpress (MCE) company); D‐Luciferin potassium salt (J&K Scientific); GM‐SCF and Bicinchoninic acid (BCA) protein assay kit (meilunbio); CCK‐8 Cell Proliferation and Cytotoxicity Assay Kit (abs50003) (Absin (Shanghai) Biotechnology Co., Ltd). Mouse CD8^+^ T Cell Isolation Kit (CS103‐01) and Mouse CD4^+^ T Cell Isolation Kit (CS102‐01) (Nanjing Vazyme Biotech Co., Ltd); Gel Rapid Extraction Kit (JiangSu CoWin Biotech (CWBIO)); StarMarker 1kb Ladder Plus (M015) and StarStain Red Plus10000× (E110) (GenStar (Beijing, China)); IL‐10 ELISA Kit (CSB‐E04594m‐IS, CUSABIO, https://www.cusabio.com/); Sparkjade ECL super (Shandong Sparkjade Biotechnology Co., Ltd.); T7 High Yield RNA Transcription kit, RNase inhibitor and ATP (Novoprotein Scientific (China)); Aminoallyl‐UTP (MedBio Pharmaceutical Technology Inc); MDM2 antibody (bs‐20790R) (Beijing Biosscn Biotechnology Co., Ltd).Anti‐p53 antibody [PAb 240] (ab26), anti‐MHC class I antibody [R1‐9.6] (ab281903), anti‐CD80 antibody (ab254579), anti‐PD‐L1 antibody [EPR20529] (ab213480), anti‐GAPDH antibody (ab8245), anti‐HMGB1 antibody (ab18256), anti‐Calreticulin antibody [EPR3924] (ab92516), goat Anti‐Mouse IgG H&L (HRP) (ab205719), goat Anti‐Rabbit IgG H&L (HRP) (ab205718), anti‐CD41 antibody [EPR17876] (ab181582) and anti‐Hemoglobin subunit alpha antibody [EPR3608] (ab92492) were ordered from Abcam (Cambridge, UK). Anti‐mouse/rat ki‐67 antibody (14‐5698‐82), anti‐Caspase 3 antibody (700 182), Alexa Fluor^TM^ 488 goat anti‐mouse IgG (A11001), Alexa Fluor^TM^ 568 goat anti‐mouse IgG (A11004), Alexa Fluor^TM^ 488 goat anti‐rabbit IgG (A11008), Alexa Fluor^TM^ 568 goat anti‐rabbit IgG (A11011), anti‐CD47 antibody (PA5‐114984) were was purchased from Invitrogen (Waltham, MA, USA). Anti‐P53 antibody (WT P53) (clone PAb1620) was purchased from Sigma (St. Louis, MO, USA). Rabbit Anti‐Granzyme B antibody (bs‐1351R) and enhanced chemiluminescence (ECL) reagent were obtained from Bioss Antibodies (Beijing, China).

The antibodies for flow cytometry including Brilliant Violet 421™ anti‐mouse CD11c (117 343), APC anti‐mouse CD86 (159 215), Brilliant Violet 650™ anti‐mouse CD86 Antibody (105 035), PE anti‐mouse CD80 (104 707), PE anti‐mouse H‐2K^b^ bound to SIINFEKL (141 604), FITC anti‐mouse CD3 (100 204), APC/Cyanine7 anti‐mouse CD3 (100 222), FITC anti‐mouse CD4 (116 003), APC anti‐mouse CD8a (162 306), APC/Cyanine7 anti‐mouse CD8a (100 714), FITC anti‐human/mouse granzyme B (GZMB) (515 403), FITC anti‐mouse IFN‐γ (505 805), PerCP/Cyanine5.5 anti‐mouse CD206 (MMR) (141 715), PE anti‐mouse/human CD11b (101 207), FITC anti‐mouse CD45 (157 213), PerCP/Cyanine5.5 anti‐mouse CD45 (103 131), Brilliant Violet 421™ anti‐mouse F4/80 (123 131), APC anti‐mouse Gr‐1 (108 411), PE anti‐mouse CD62L (161 203), Alexa Fluor® 647 anti‐mouse FOXP3 (320 014), APC anti‐mouse/human CD44 (103 012), and TruStain FcX™ PLUS (anti‐mouse CD16/32) Antibody (156 603), were purchased from Biolegend (San Diego, CA, USA). Ghost Dye™ UV 450 Fixable Viability Dye (80 862) was purchased from Cell Signaling Technology (Danvers, MA, USA).

### Cell Lines

4T1 murine breast cancer cells, MC38 murine colorectal cancer cell line, Luc‐4T1 cells (catalog no. LZQ0016) were purchased from the National Infrastructure of Biomedical Cell Line Resource (Beijing, China). 4T1‐OVA cell line (CBPG0020) and MC38‐OVA cell line (CBPG0016) were purchased from Nanjing Cobioer Biosciences Co., Ltd (Nanjing, China). Furthermore, Luc‐4T1 cells, MC38 cells, 4T1‐OVA cells and MC38‐OVA cells were cultured and maintained in RPMI 1640 culture medium (Procell Life Science&Technology Co., Ltd.). 4T1 cells were cultured in SNLM‐113 (Wuhan Sunncell Biotechnology Co., Ltd.). Both media were supplemented with 10% fetal bovine serum (Lonsera, Suzhou Shuangru Biology Science& Technology Co., Ltd) and 1% penicillin‐streptomycin. All cells were cultured in an atmosphere at 37 °C with 5% CO_2_. CELLSAVING (Cat.No.C40100) was purchased from New Cell & Molecular Biotech.

### Preparation of PRIZE

Pre‐prepared RBCM were thoroughly mixed with Z@R@I in a mass ratio of 3:1. The mixture was successively filtered through membranes with pore sizes of 800, 450, and 200 to obtain RBCM‐Z@R@I. 1mg of DSPE‐PEG‐cRGD peptide and DSPE‐PEG‐L17E peptide were dissolved separately in 1 mL of DMF. Subsequently, 100 µL of each solution was added dropwise to 1 mL of RBCM‐Z@R@I solution (3 mg mL⁻^1^). After incubating at 37 °C for 2 h, the mixture was centrifuged at 12000 rpm for 10 min to yield PRIZE.

### In Vitro Cellular Uptake and Analysis of Lysosomal Escape

For the cellular uptake study, 4T1 cells were seeded at 35 mm confocal dish at 4 × 10^4^ per dish and cultured for 24 h. After removing the culture medium, cells were treated with Cy5‐labeled PRIZE at 37 °C for different times. To quantify cell uptake, the cells were washed three times with PBS and then collected for flow cytometry analysis. To visualize the lysosomal escape of nanoparticles, 4T1 cells were treated with RGD‐RBCM‐Z@R@I and PRIZE for 0, 1, 2, and 4 h. The cells were then stained using LysoTracker Green (50 nM) at 37 °C for 8 min and washed twice with PBS. Finally, cell nuclei were stained with Hoechst 33 342 (1 ×) for 10 min and washed three times with PBS before being imaged by laser scanning confocal microscope (CLSM600, Sunny Optical Technology, China).

### Analysis of qRT‐PCR

4T1 cells (5 × 10^5^) were cultured in six‐well plates for 24 h. Then, the cells were treated with PBS, PIZE, PRIZE for 4 h at doses equiv. to 30 µM. The total mRNA was isolated by TRIeasy^TM^ Total RNA Extraction Reagent (10606ES60; Yeasen, Shanghai, China) following the manufacturer's manual. The complementary DNA (cDNA) was synthesized using a high‐capacity cDNA reverse transcription kit (Applied Biosystems). The qRT‐PCR was carried out using PerfectStart® Green qPCR SuperMix (cat. no. AQ601‐01; TransGen Biotech, China) and detected in BioRad CFX96 touch qRT‐PCR detection system. The relative gene expressions were analyzed by CFX maestro software.

### Analysis of In Vivo Cytokine Concentrations

The tumor tissues of the mice were collected 2 days after various treatments. Then, the weights of the tumor tissues were weighed accurately, and the tumor tissues were mixed with normal saline (the proportion of tissue weight (mg): normal saline (µL) = 1:9). The mixtures were homogenized under ice bath for 10 min to obtain the 10% homogenization suspensions. Next, the suspensions were centrifuged for 10 min at 3000 rpm, and the supernatants were collected for ELISA assay to determine the intratumoral cytokine contents according to the manufacturer's instructions.

### Statistical Analysis

All quantitative data are presented as mean ± standard deviation (S.D.) from three or more independent runs. Significant differences between different groups were determined using two‐tailed unpaired Student's t‐tests for two‐group comparisons and one‐way analysis of variance (ANOVA) with post hoc Tukey's test for multiple‐group comparisons (*P <0.05, **P <0.01, ***P <0.001 and ****P <0.0001, ns, not significant). *P* <0.05 is considered a statistically significant difference. Statistical analyses were performed on Graphpad Prism 8.3.2.

## Conflict of Interest

The authors declare no conflict of interest.

## Supporting information



Supporting Information

Supporting Information Movie 1

Supporting Information Movie 2

## Data Availability

The data that support the findings of this study are available from the corresponding author upon reasonable request.
