# Supplementary material for: Restoring Tumor Cell Immunogenicity Through Ion‐Assisted p53 mRNA Domestication for Enhanced In Situ Cancer Vaccination Effect
Source: Adv Sci (Weinh). 2025 Feb 18;12(14):2500825. doi: 10.1002/advs.202500825 (PMC11984859; doi:10.1002/advs.202500825)
Supplement: Supplementary file 1 — Supporting Information [file ADVS-12-2500825-s002.docx]

Supporting Information

Restoring Tumor Cell Immunogenicity through Ion-assisted *p53* mRNA Domestication for Enhanced In-Situ Cancer Vaccination Effect

Yan Liang^#^, Jingge Zhang^#^, Jinjin Wang, Yuhe Yang, Xinyu Tan, Shuguang Li, Zhenzhen Guo, Zhenzhong Zhang, Junjie Liu*, Jinjin Shi*, Kaixiang Zhang*

**Experimental Section**

*Animals*: Female BALB/C mice (5-week-old) and C57BL/6 mice (5-week-old) were purchased from Beijing HFK Bio-Technology. All the mice (three to five mice per cage) were housed in standard, infection-free housing room, with 12 h light:12 h dark cycles, stable temperature at ~23°C, and stable humidity of ~40% in the vivarium at the Zhengzhou University. All animal studies were carried out following the guidelines of the Regional Ethics Committee for Animal Experiments and the Care Regulations approved by the Institutional Animal Care and Use Committee of Zhengzhou University. The animal laboratory's accreditation number is 110 322 211 102 955 054.

*Preparation of ZIF-90*: 5.77 mg of 2-ICA was dissolved in 400 μL of N,N-dimethylformamide (DMF) to obtain the organic phase, which was magnetically stirred. 1.97 mg of Zn (OAc)_2_·2H_2_O was dissolved in 90 μL of DEPC-treated water to form the aqueous phase. The aqueous phase was rapidly added to the organic phase and vigorously stirred for 10 minutes. The resulting formulation was centrifuged (1500 rpm, 3 min) and the supernatant was discarded. The precipitate was washed sequentially with 500 μL of anhydrous ethanol, centrifuged (6500 rpm, 3 min), and the supernatant was discarded again. The precipitate was washed twice with 500 μL of DEPC-treated water, centrifuged (6500 rpm, 3 min), and the supernatant was discarded to obtain ZIF-90.

*Preparation of Z@R@I*: 5.77 mg of 2-ICA was dissolved in 400 μL of DMF to obtain the organic phase, which was magnetically stirred. 1.97 mg of Zn (OAc)_2_·2H_2_O was dissolved in 100 μL of DEPC-treated water to form the aqueous phase. After incubating 10 μg of mRNA with the aqueous phase for 5 minutes, the aqueous phase was rapidly added to the organic phase and vigorously stirred for 10 minutes. The resulting formulation was centrifuged (1500 rpm, 3 min) and the supernatant was discarded. The precipitate was washed sequentially with 500 μL of anhydrous ethanol, centrifuged (6500 rpm, 3 min), and the supernatant was discarded again. The precipitate was washed twice with 500 μL of DEPC-treated water, centrifuged (6500 rpm, 3 min), and the supernatant was discarded to obtain the precipitate. The precipitate was resuspended in 500 μL of boric acid solution with a pH of 8.5, mixed with 20 μL of amino-modified ICG (3 mg/mL) and incubate at 37°C for 30 minutes. Centrifuge at 6500 rpm for 3 minutes, discard the supernatant to obtain Z@R@I.

*Characterization of ZIF-90, Z@R@I, RBCM-Z@R@I, PRIZE*: A Malvern Zeta Sizer Nano-ZS instrument was selected to detect the particle size, polydispersity index (PDI) and zeta potential of ZIF-90, Z@R@I, RBCM-Z@R@I, PRIZE. Transmission electronic microscopy (TEM) was selected to visualize the morphology of ZIF-90, Z@R@I, PRIZE. To assess the stability of PRIZE, measurements were taken of size changes, PDI, ICG and mRNA leakage in PBS, medium, and medium with 10% FBS over a span of 1 to 7 days.

*Capillary electrophoresis examination of mRNA stability*: Z@R@I samples, stored in DEPC-treated water at 4°C for different durations, were degraded using 5 mM ATP. Subsequently, RNA integrity was assessed following the instructions provided for capillary electrophoresis (BIOptic Qsep1. New Taipei City, Taiwan).

*Evaluation of near-infrared performance of NPs*: To evaluate photothermal performance *in vivo*, 9 hours after intravenous injection of PRIZE, the tumor site in the mice was continuously irradiated (808 nm, 2 W/cm^2^). The temperatures were recorded by an infrared thermal camera (FLIR E40, USA) and treated with associated software (FLIR Tools, version 4.0.13330.1003) every 15 s during NIR laser irradiation. All the experiments for evaluating NIR photothermal performances were carried out at the same room temperature.

*Cell immunofluorescent staining*: 4T1 and MC38 cells were seeded in 6-well plates (2 × 10^5^ cells per well) for 24 h and treated with different nanocomplexes (30 μg/mL) for 4 h, respectively. After that, the medium was replaced with fresh one and the incubation was continued for 24 hours. Then, the cells were fixed with 4% paraformaldehyde (PFA) and permeabilized treatment with 0.1%Triton X-100 for 10 min, washed with PBS for three times, and incubated with 5% BSA for 1 hour at room temperature. After removing the BSA with three washes of PBS, the samples were incubated with the primary antibodies overnight at 4°C. Following three washes with PBS, the samples were incubated with the corresponding fluorescence-labeled secondary antibodies for 30 minutes at room temperature. The samples were further stained with DAPI, washed with PBS, and mounted on coverslips for imaging with CLSM.

*Measurement of ICD in vitro*: The ICD effect was characterized by pro-apoptotic CRT cell surface overexpression, extracellular HMGB1 release, and ATP secretion. 4T1 cells were seeded in 6-well plates (2 × 10^5^ cells per well) for 24 h and treated with different nanocomplexes (30 μg/mL) for 4 h. After that, the PRIZE+NIR group was irradiated (808 nm, 2.0 W/cm²) for maintaining 43℃ 5 minutes, then the medium was replaced with a fresh one and the incubation was continued for 24 hours. Next, the cells were imaged as described in the “Cell immunofluorescent staining” section. Extracellular ATP detection: 4T1 cells were seeded in 6-well plates (2 × 10^5^ cells per well) for 24 h. After that, the cells were treated with different nanocomplexes (30 μg/mL) for 12 h. Then, the supernatant in 6-well plates were collected respectively to detect the extracellular ATP concentrations by the ATP assay Kit.

*In vitro maturation of BMDCs and activation of CD8^+^ T cells*: To investigate the maturation of BMDCs, 4T1-OVA cells (1 × 10^5^) were seeded on the top wells (i.e., inserts) of transwells and treated with PBS, PZE, PRZE, PRIZE and PRIZE+NIR for 8 h. Afterward, immature BMDCs were seeded in the bottom wells of the transwells at 1 × 10^6^ cells per well. At 24 h, the BMDCs were collected and stained with Brilliant Violet 421™ anti-mouse CD11c, PE anti-mouse CD86, PE anti-mouse CD80. For the detection of T cell activation, CD8^+^ T cells were extracted from mouse spleens using a reagent kit. Then, CD8^+^ T cells (1 × 10^6^) were co-cultured with the pre-incubated BMDCs (1 × 10^5^) in 96-well plate. After 3 days, cells were stained with APC/Cyanine7 anti-mouse CD3, APC anti-mouse CD8a, and FITC anti- mouse GZMB antibodies, and analyzed by flow cytometry. The co-culture medium was also collected to examine the secretion of IL-12p70 and TNF-α using ELISA kits by following the protocols provided by the manufacturer.

*In vitro anticancer capability via activating CD8^+^ T cells*: 4T1 cells were seeded in µ-Dish^35mm, low^ (ibidi, Fitchburg, WI, USA) at a density of 1 × 10^4^ cells per dish and placed in the cell-incubator stage (mounted on the LEICA TCS SP8 STED) maintained at 37°C with 5% CO_2_. The CD8^+^ T cells (2 × 10^3^ per dish) activated were labeled with Cell Tracker™ DiI Dye (0.5 µM) as aforementioned were added into the dishes with 4T1 cells and monitored for 4 h under microscope.

*Western blot analysis*: To perform western blot analysis, the cytopr teins of 4T1 cells after different treatments were collected. Specifically, the RIPA lysis buffer was added to the suspended cells (250 μL/10^6^ cells). After repeated aspiration for 30 min (ice bath), the supernatant was collected by centrifugation (12000 rpm, 10 min, 4°C). Then the Western blot analysis of P53 was performed following the standard protocol. β-actin was used as the loading control.

*RNA-seq analysis*: The BALB/C mice (4–6 weeks, female) were randomly divided into the same four groups and receiving corresponding treatments as above. Total RNA of different treatments was extracted using Trizol reagent kit (Invitrogen, Carlsbad, CA, USA) according to the manufacturer’s protocol.

*In vivo biodistribution of NPs*: The *in vivo* distribution of NPs was investigated in BALB/C mice bearing 4T1 tumor. In brief, a total of 1 × 10^6^ 4T1 cells were injected into the axillary region of the right forelimb of each mouse. When the volume of tumor reached approximately 100 mm^3^, mice were randomly distributed into three groups and intravenously injected with saline, free Cy5-mRNA, Z@R@I, RBCM-Z@R@I and PRIZE. At 1, 3, 6, 9, 12, 24 and 30 h, whole-animal images of all mice were acquired using a PerkinElmer (Waltham, MA, USA) IVIS instrument. At the end of the study, mice were euthanized and their critical organs including hearts, livers, spleens, lungs, kidneys, and tumors were harvested and imaged by IVIS. Moreover, circulation of NPs in blood was also studied by collecting blood from the mice at various time points and imaged by a microplate reader.

*In vivo expression of mRNA*: Using Luciferase mRNA, PRIZE nanoparticles and free Luc mRNA were prepared for *in vivo* imaging. At various time points post-intravenous administration (with mRNA content of 3 μg), *in vivo* imaging was conducted on 4T1 tumor-bearing BALB/C mice using the IVIS imaging system. Following imaging, mice were euthanized, and major organs (heart, liver, spleen, lungs, kidneys) along with tumors were dissected for ex vivo fluorescence imaging.

*Ex vivo immunofluorescence analysis*: After various treatments, the tumors were harvested and sectioned. The slices were then stained according to the procedure outlined in the “Cell Immunofluorescent Staining” section.

*The detection of oxygen levels and blood flow velocity in tumors*: The photoacoustic (PA) imaging was used to detect oxygen content in tumor tissues (Vevo® LAZR 2100, Visual Sonics, Canada). First, 4T1 tumor-bearing mice were established. After the tumor grew to 200 mm^3^, the oxygen content detection module of PA imaging was used to detect the blood oxygen status of the tumor tissues. Subsequently, PRIZE was administered to the mice via tail vein injection. After 24 hours, laser irradiation was performed, and the oxygen content in the tumor tissues was measured both before and after laser irradiation. The analysis software was used to quantify the oxygen content in the tumor tissue.

*Antitumor activity and safety evaluation of the 4T1 bilateral tumor model*: To establish a bilateral tumor model, 1 × 10^6^/100 μL 4T1 tumor cells in PBS were subcutaneously transplanted into the right flank of a female BALB/C mice as the primary tumor, then the distant tumor was conducted via injecting 4T1 cells (3.0×10^5^ cells per mouse) into the subcutaneously left flank of the same mouse. When the primary tumor volume reached about 100 mm^3^, the mice were randomly divided into four groups and were intravenously injected with 200 µL of saline, PIZE+NIR, PRIZE, or PRIZE+NIR every three days, for a total of five injections. For the PIZE+NIR or PRIZE+NIR treated groups, an additional laser irradiation (808 nm, 2.0 W/cm²) for maintaining 43℃ 5 minutes was applied to the right (primary) tumor 24 hours post-injection under the supervision of a thermal imager. Both PIZE and PRIZE were administered in a nano-formulation with an identical ZIF-90 dose of 155 μg per injection. The length (L) and width (W) of the subcutaneous tumors and the body weights were measured every other day after administration. The tumor volumes were calculated by the formula of (L × W^2^)/2. When the tumor volume was larger than 2000 mm^3^, the mice were euthanized by cervical dislocation. At the end of therapy, some mice were sacrificed and the main organs (heart, liver, spleen, lung, and kidneys), as well as bilateral tumors, were harvested, fixed in 4% paraformaldehyde solution, and sectioned into slices for H&E, TUNEL, Ki67 and Caspase3 staining. For safety evaluation, At the end of treatment, histological samples of the main organs excised (heart, liver, spleen, lungs, and kidneys) were collected for pathological evaluation, as well as blood samples for hematological and blood biochemical analysis.

*Ex Vivo Detection of Tumor-Infiltrating Lymphocytes*: Immune Cell Analysis: Following assessment of *in vivo* anti-tumor activity, immune cells and their secreted cytokines were analyzed. Tumors, lymph nodes, spleens, and blood were further analyzed. Simply put, obtained tumors were cut into small pieces and digested with DNAse I (0.1 mg mL^−1^, Roche, Basel, Switzerland) and Collagenase IV (2.0 mg mL^−1^, Roche) at 37°C for 1 hour. Single-cell suspensions were filtered through a 70 µm filter prior to flow cytometry analysis, and stained with the following marker combinations and gating: Dendritic cells (CD11c^+^ CD80^+^ CD86^+^), (CD45^+^ CD11c^+^ MHC I^+^), infiltrating T cells (CD3^+^ CD4^+^ CD8^+^), activated CD8 (CD3^+^ CD8^+^ GZMB^+^), (CD3^+^ CD8^+^ IFN-γ^+^), tumor-associated macrophages (CD45^+^ CD11b^+^) M1 (F4/80^+^ CD206^−^ CD86^+^), tumor-associated macrophages M2 (F4/80^+^ CD206^+^ CD86^−^), myeloid-derived suppressor cells (MDSCs, CD11b^+^ Gr-1^+^), Treg cells (CD3^+^ CD4^+^ Foxp3^+^). T cells (CD3^+^ CD4^+^ CD8^+^) from collected lymph nodes, blood, and spleen cells, as well as dendritic cell maturation in lymph nodes (CD86^+^ CD80^+^), (CD11c^+^ MHC I^+^), were determined, and flow cytometry data were analyzed using FlowJo (v10, BD). Moreover, for the bilateral 4T1 tumor model and pulmonary metastasis model, the cytokines IFN-γ, TNF-α, IL-12p70 and IL-10 were detected in serum using ELISA Kits.

*Evaluation of metastasis and recurrence*: To construct the lung metastasis model, 1 × 10^6^ 4T1 tumor cells were transplanted into the flank of the mice. When tumors reached about 100 mm^3^, mice were randomly divided 4 into groups, and intravenously injected with different formulations like before. After 24 h, the mice were intravenously injected with 4T1-Luc cells (1 × 10^5^). After intraperitoneal injection of luciferin (150 mg/kg), the lung metastasis of 4T1-Luc was imaged with an IVIS Lumina II under anesthesia. After another 15 days, the mice were killed, and their lungs were excised. Lung metastasis nodules were then manually counted, and lung tissue sections were subjected to H&E staining.

To construct the recurrence model, BALB/C mice were subcutaneously transplanted with 1 × 10^6^ 4T1 tumor cells. When tumors reached about 100 mm^3^, mice were randomly divided into 4 groups, and intravenously injected with different formulations like before. On day 15, tumors in different groups were removed by surgery. On day 19, the mice were challenged by subcutaneous 1 × 10^6^ 4T1 tumor cells. Tumor growth was evaluated every four days. On day 60, splenocytes were isolated and the percentage of effect memory T cells (CD3^+^ CD8^+^ CD44^+^ CD62L^−^) and century memory T cells (CD3^+^ CD8^+^ CD44^+^ CD62L^+^) was detected by flow cytometry.


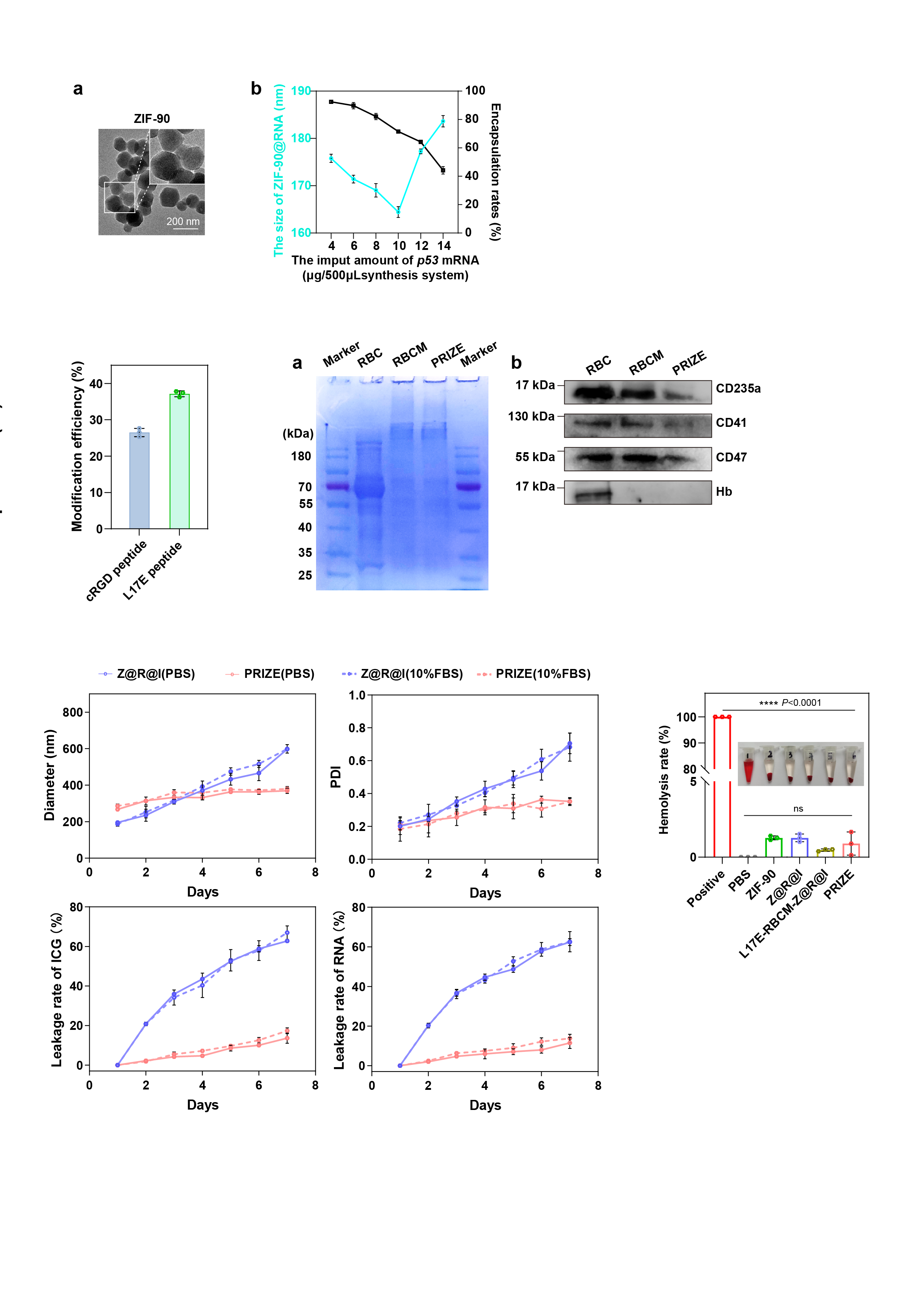


**Figure S1.** a) Representative TEM image of ZIF-90. b) The mRNA encapsulation efficiency and particle size of ZIF-90@*p53* mRNA synthesized with different *p53* mRNA input amount (n = 3 independent samples).


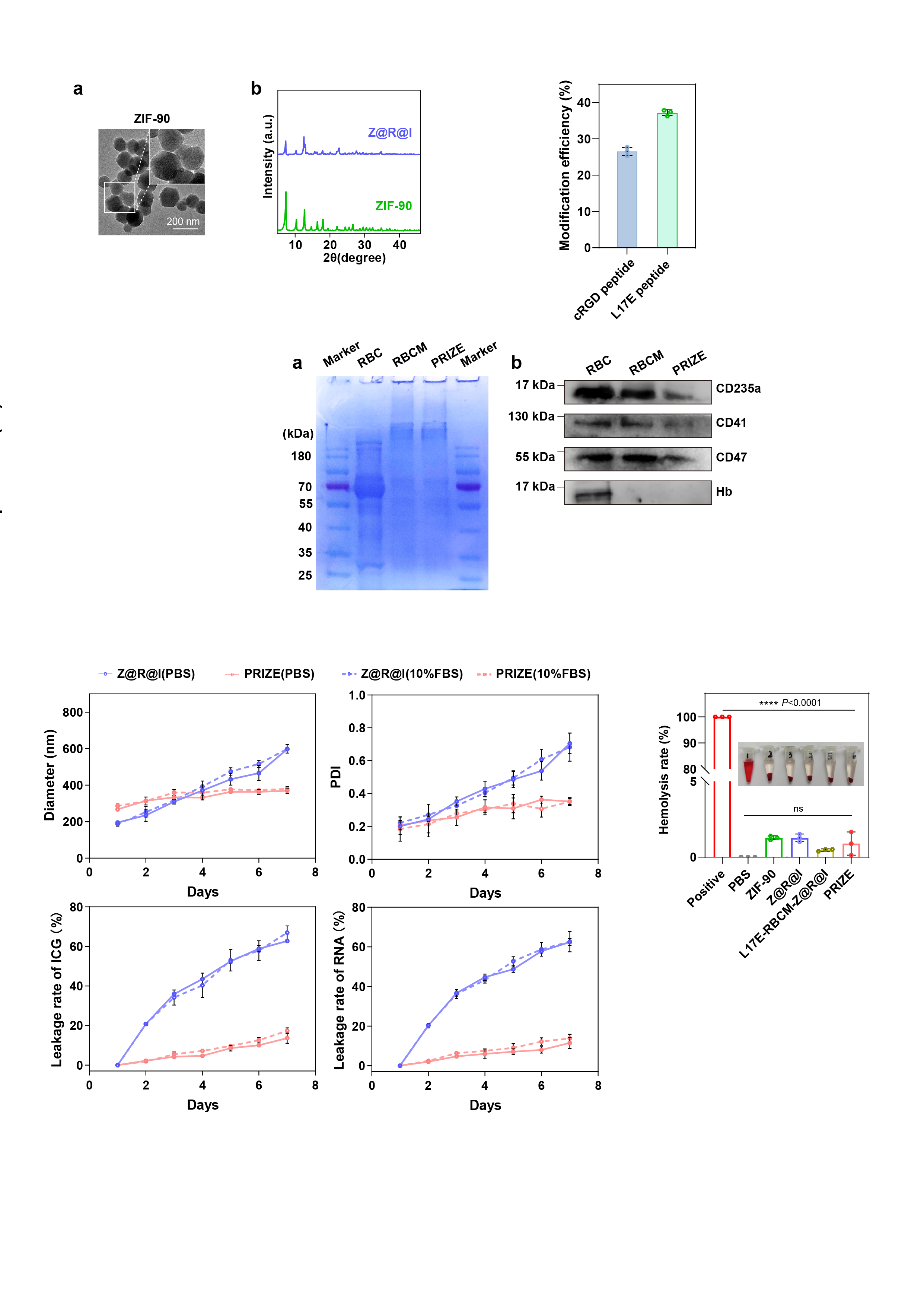


**Figure S2.** The modification efficiency of cRGD peptide and L17E peptide on the surface of RBCM (n = 3 independent samples).


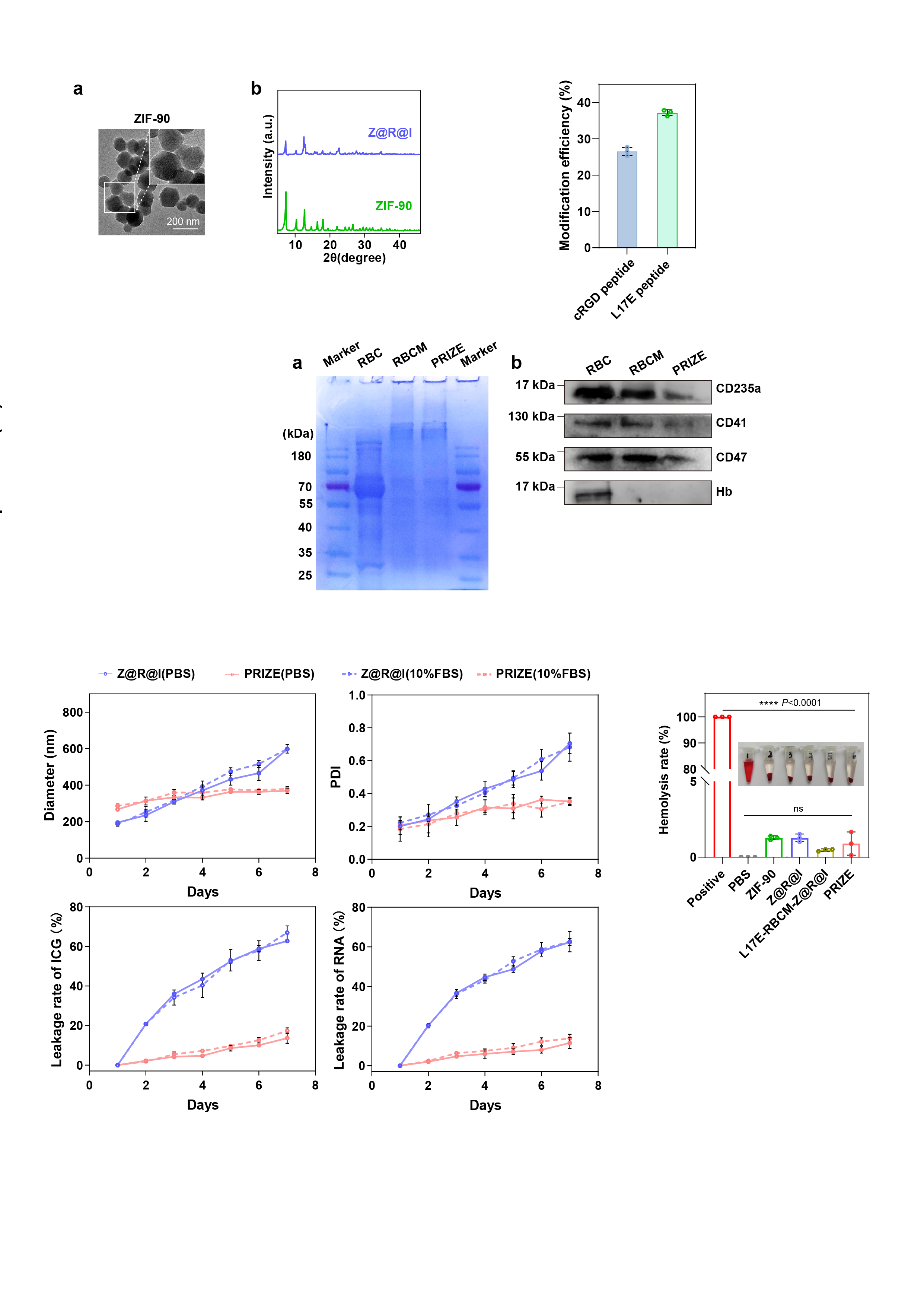


**Figure S3.** The stability of Z@R@I and PRIZE in 10% FBS or PBS within 7 days (n = 3 independent samples). Data are shown as mean ± SD.


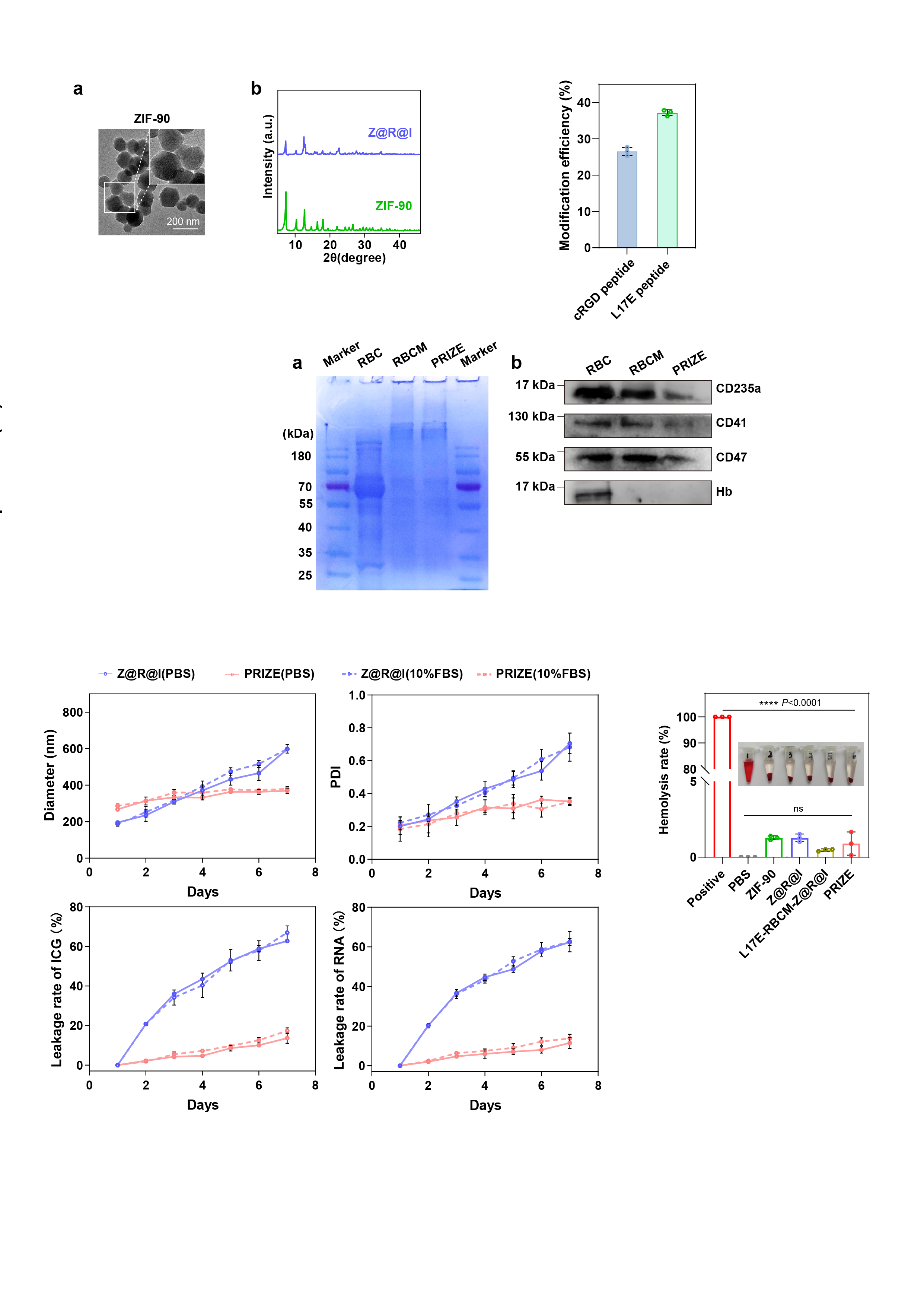


**Figure S4.** In vitro hemolysis rates of ZIF-90, Z@R@I, L17E-RBCM-Z@R@I and PRIZE. Statistical analyses were done using one-way ANOVA with Tukey’s multiple comparisons test and correction (n = 3 biologically independent samples). **P* < 0.05, ***P* < 0.01, ****P* < 0.001, *****P* < 0.0001, ns, not significant. Data are shown as mean ± SD.


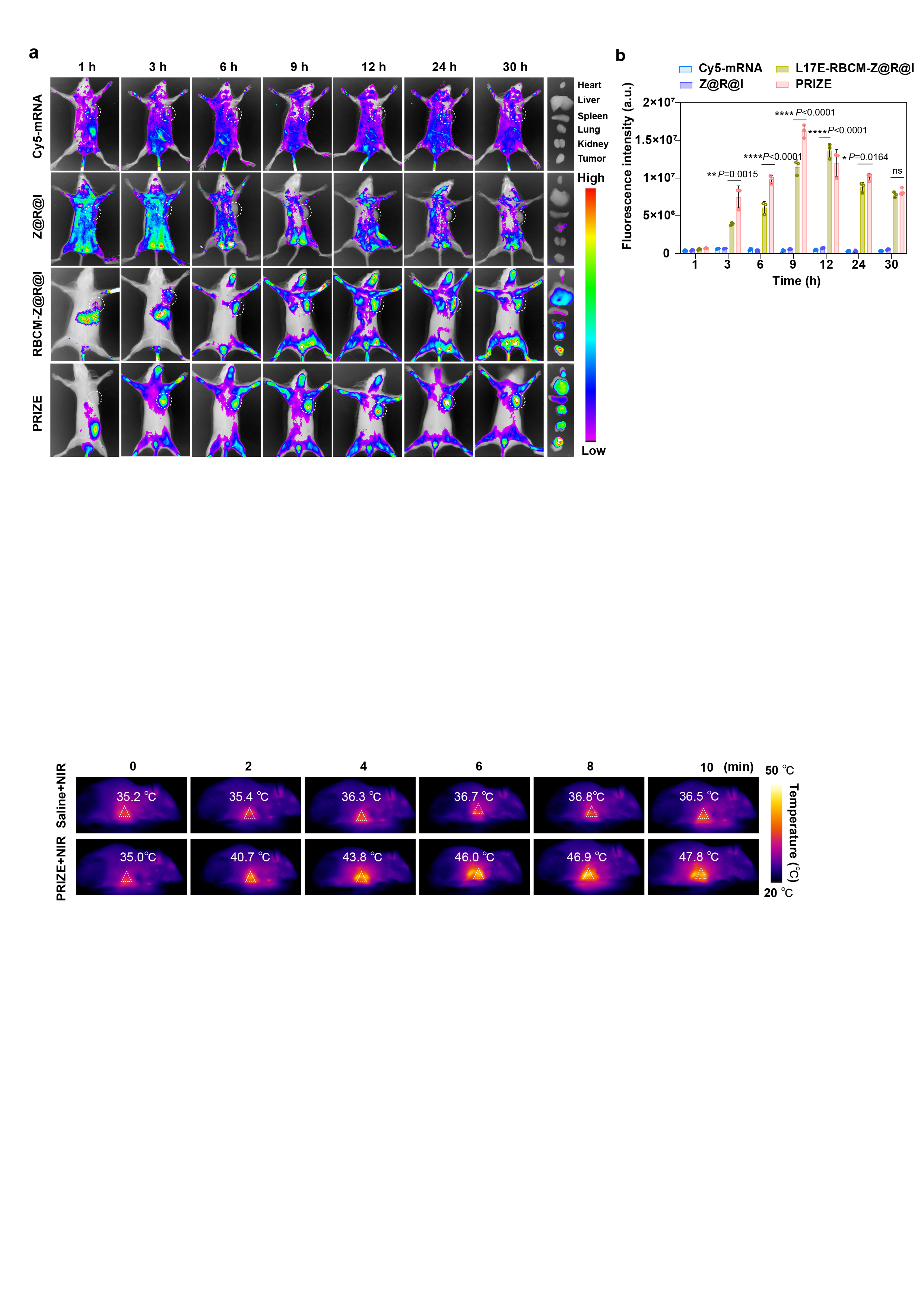


**Figure S5.** *In vivo* fluorescence imaging after intravenous injection of various formulations with Cy5-*p53* mRNA in 4T1 tumor-bearing mice at different time points (a) and the corresponding quantification of mean fluorescence intensity (MFI) values (b). (a) Typical IVIS images showing the Cy5 fluorescence in different organs including tumors collected from mice injected via tail vein with various formulations with Cy5-*p53* mRNA at the end of *in vivo* fluorescence imaging (n = 3 biologically independent samples). Statistical analyses were done using one-way ANOVA with Tukey’s multiple comparisons test and correction. **P* < 0.05, ***P* < 0.01, ****P* < 0.001, *****P* < 0.0001, ns, not significant. Data are presented as mean ± SD.


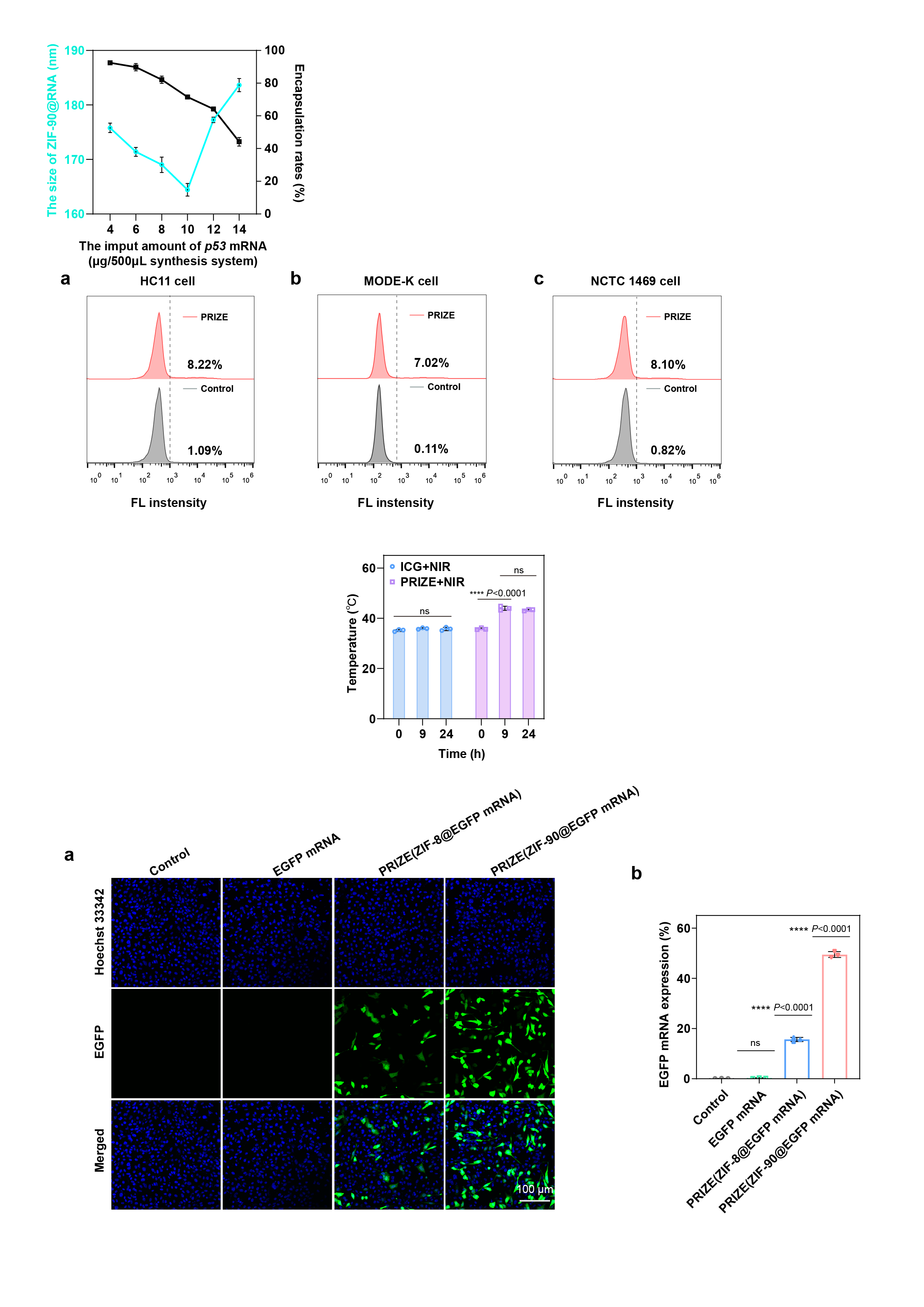
**Figure S6.** Evaluation of PRIZE’s photothermal conversion efficiency at tumor site (n = 3 biologically independent samples). Statistical analyses were done using one-way ANOVA with Tukey’s multiple comparisons test and correction. **P* < 0.05, ***P* < 0.01, ****P* < 0.001, *****P* < 0.0001, ns, not significant. Data are presented as mean ± SD.


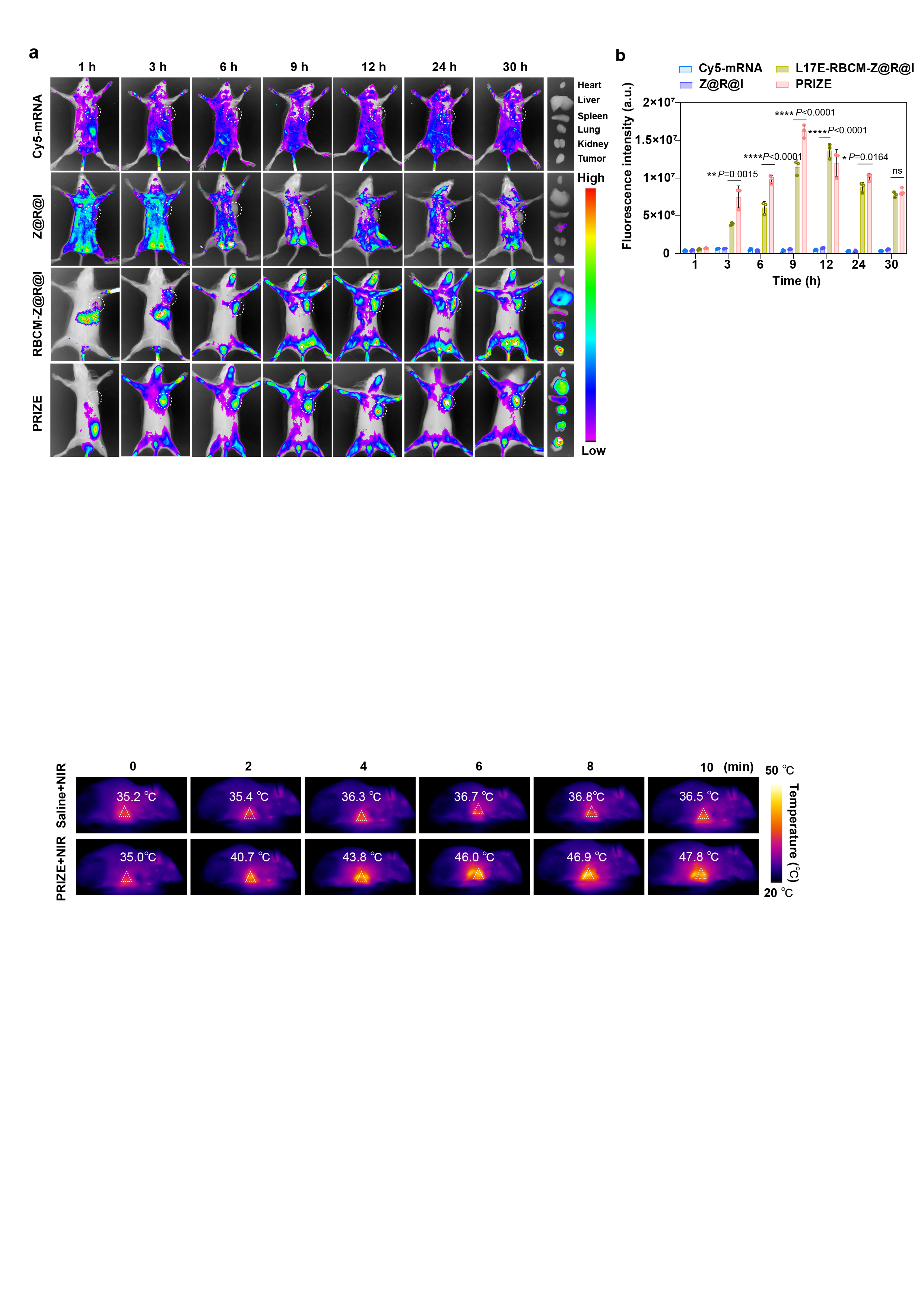


**Figure S7.** Infrared thermal images of mice treated with saline or PRIZE under persistent 2.0 W/cm^2^ laser irradiation (808 nm).


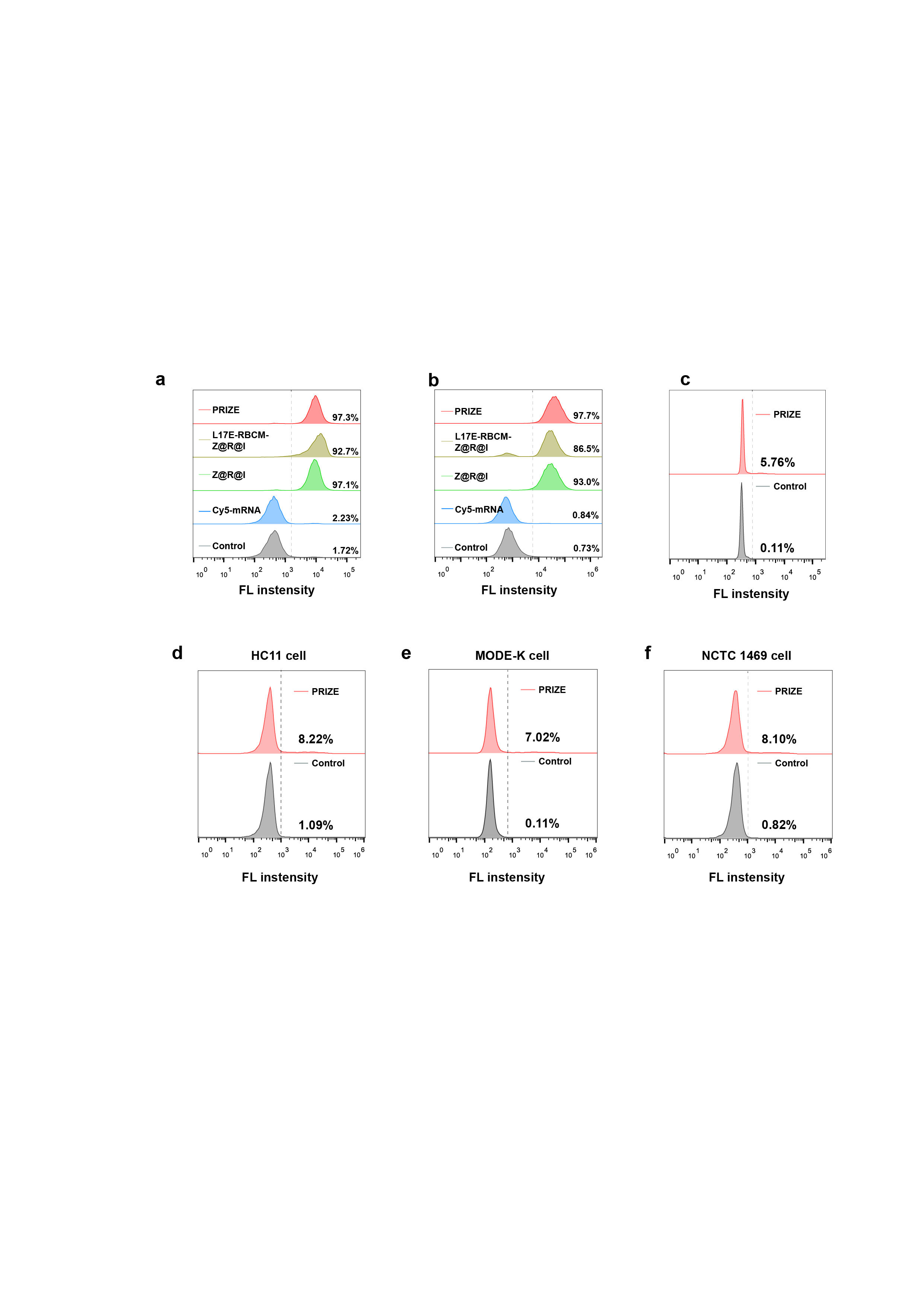


**Figure S8.** PRIZE enhanced cellular uptake mRNA in cancer cells with negligible uptake in RAW264.7 cells. a-b) Flow cytometry analysis of the intracellular internalization of (a) 4T1 cells (b) MC38 cells after incubation with free *p53* mRNA, Z@R@I, L17E-RBCM-Z@R@I and PRIZE (*p*53 mRNA labeled with Cy5) for 4 h. c) Flow cytometry analysis of cellular uptake of PRIZE in RAW264.7 cells after incubation for 4 h.


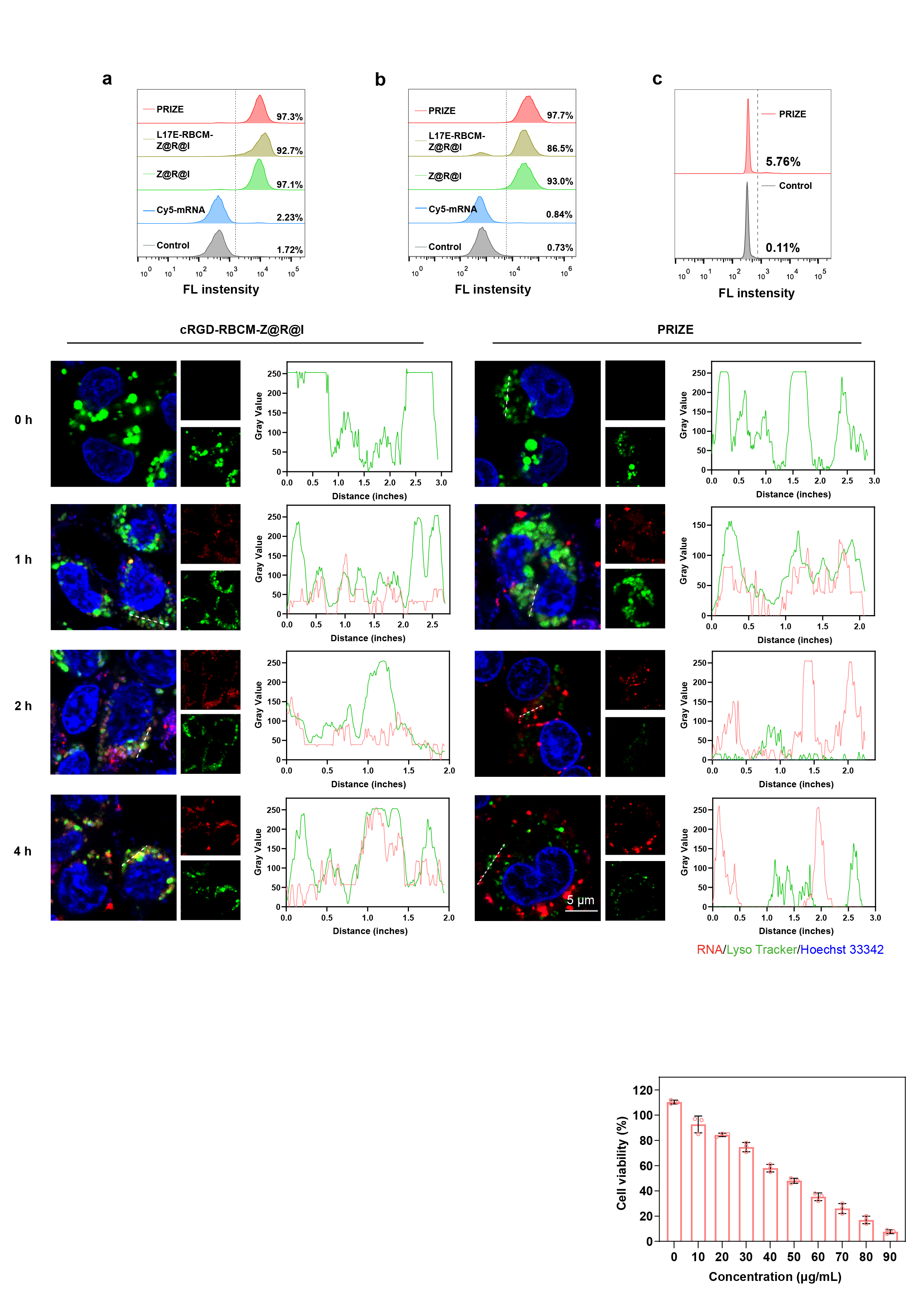


**Figure S9.** Subcellular localization imaging of NPs in 4T1 cells with or without L17E peptide was conducted via CLSM, employing various incubation time intervals as indicated on the scale bar (5 μm).


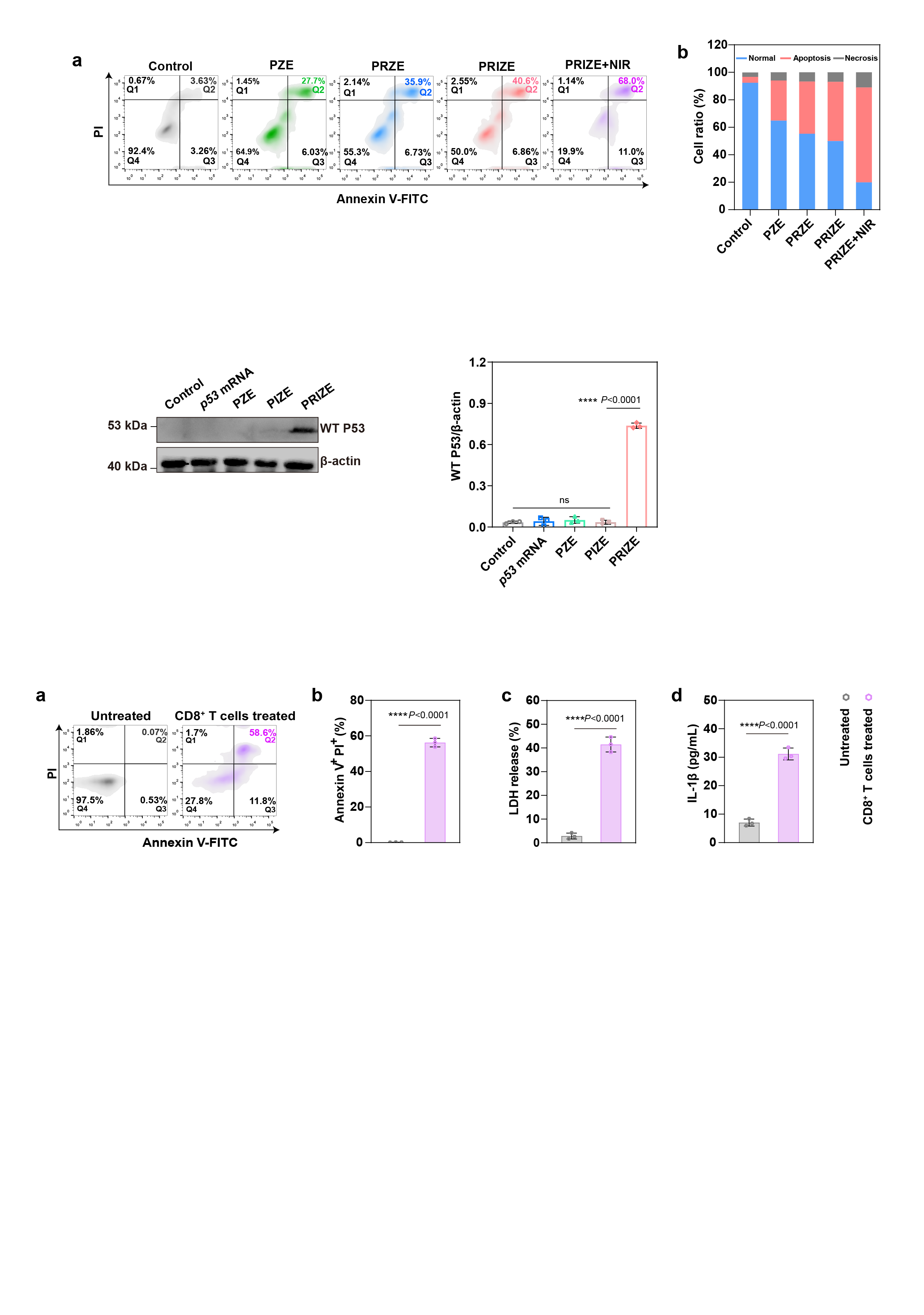


**Figure S10.** Western blot and quantitative analysis of P53 expression after treatments. (n = 3 biologically independent samples). Statistical analyses were done using one-way ANOVA with Tukey’s multiple comparisons test and correction. **P* < 0.05, ***P* < 0.01, ****P* < 0.001, *****P* < 0.0001, ns, not significant. Data are presented as mean ± SD.


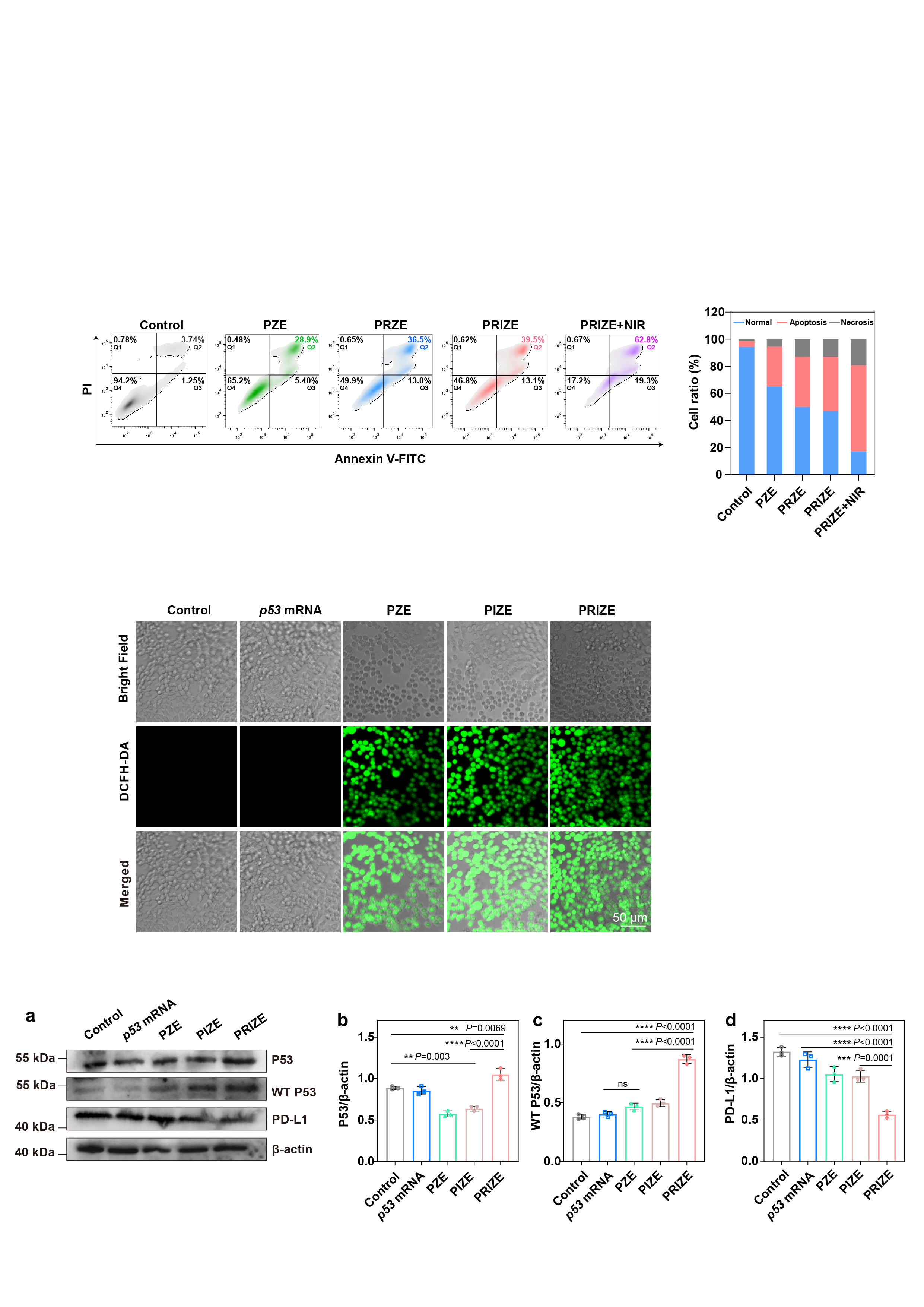


**Figure S11**. CLSM images of ROS levels of MC38 cells in various treatment groups, the 2, 7-dichlorodihydrofluorescein diacetate probe was used as a fluorescent probe to stain intracellular ROS.

**
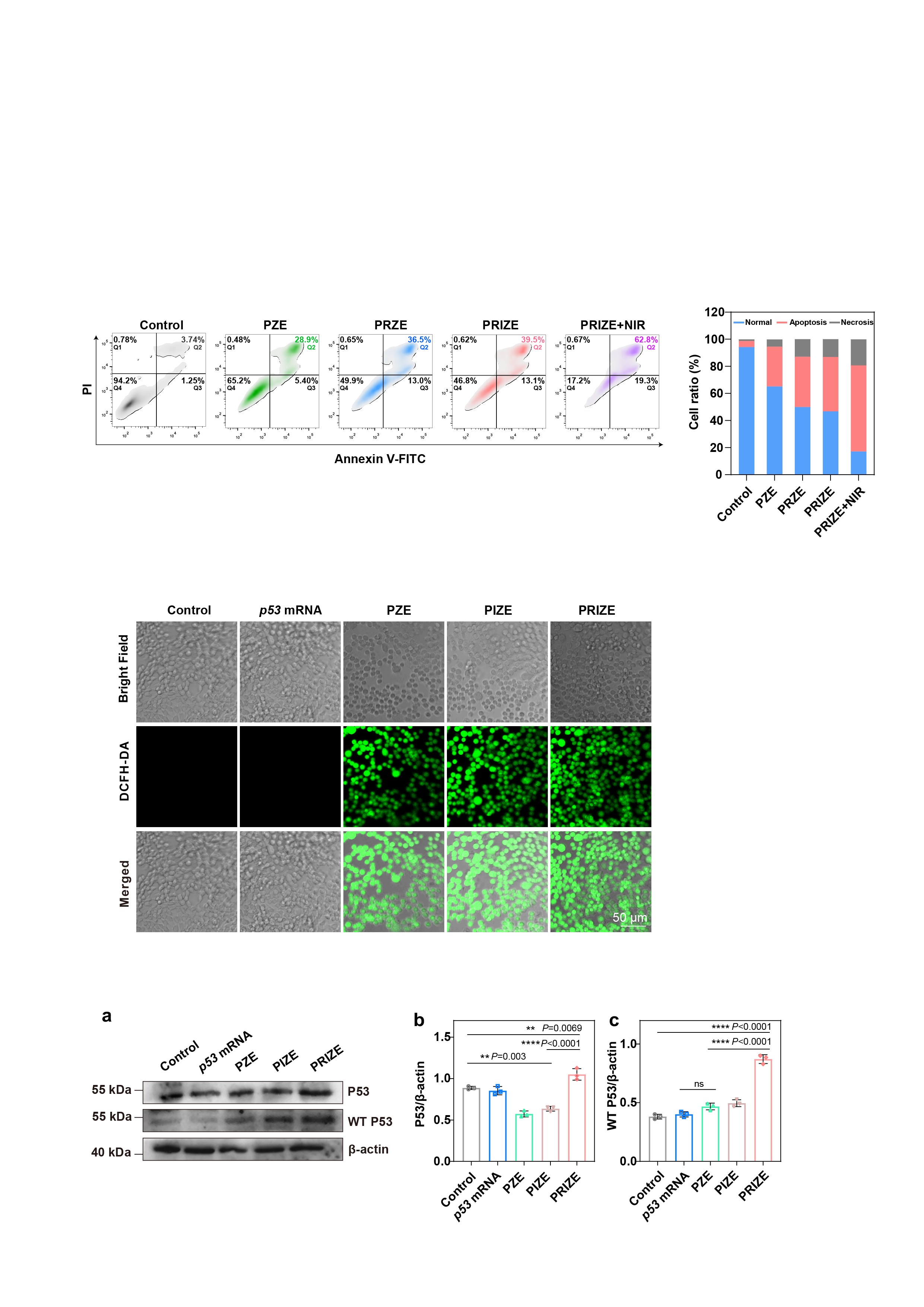
**

**Figure S12**. a) Western blot analysis of total P53, WT P53 protein in MC38 cells with different treatments. b**-**c) Relative protein amount was quantified by image densitometry (n = 3 biologically independent samples). Statistical analyses were done using one-way ANOVA with Tukey’s multiple comparisons test and correction. **P* < 0.05, ***P* < 0.01, ****P* < 0.001, *****P* < 0.0001, ns, not significant. Data are presented as mean ± SD.


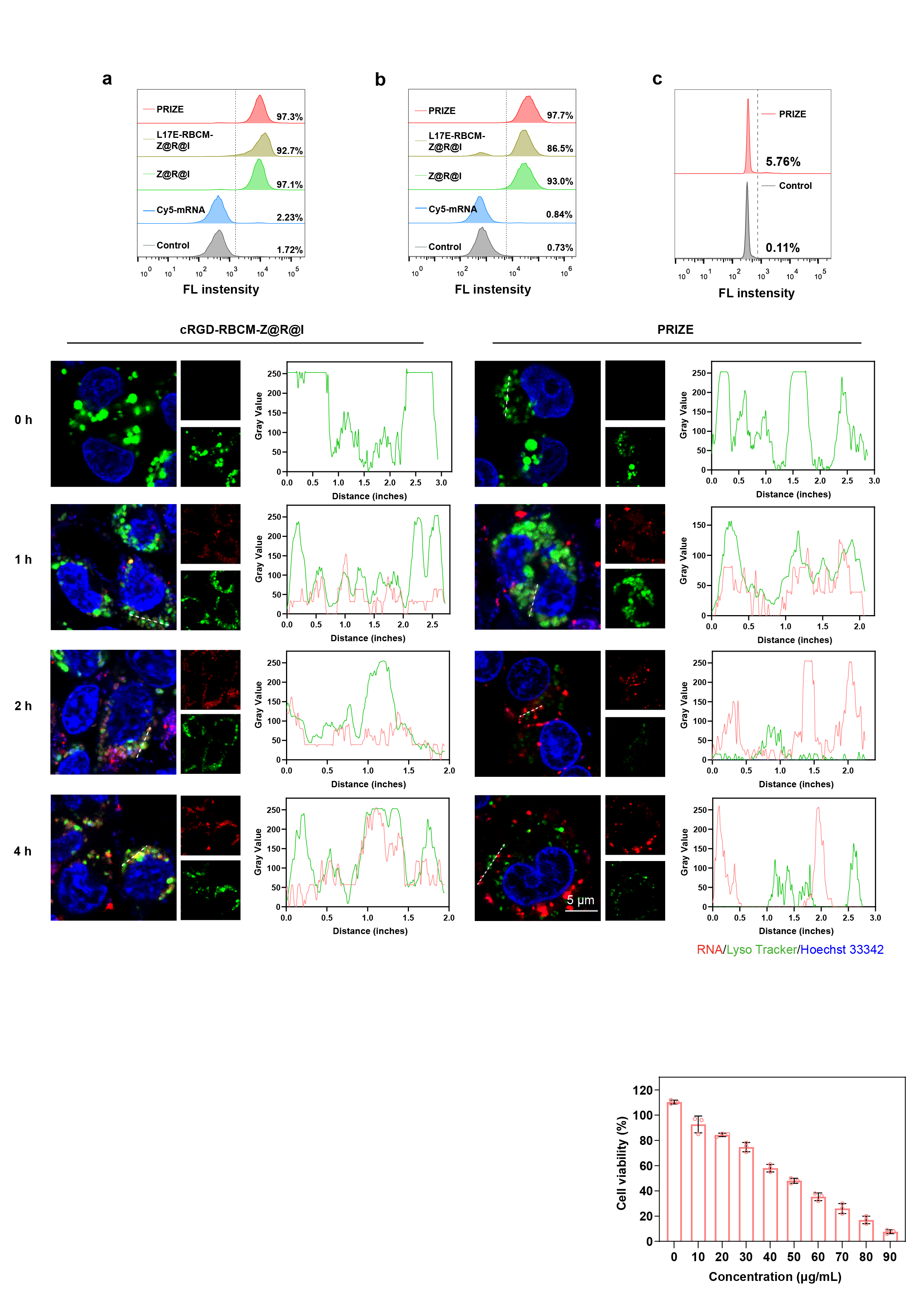


**Figure S13.** Relative viability of 4T1 cells after 24 h treatment with different concentrations of PRIZE (n = 3 biologically independent samples).

.

**
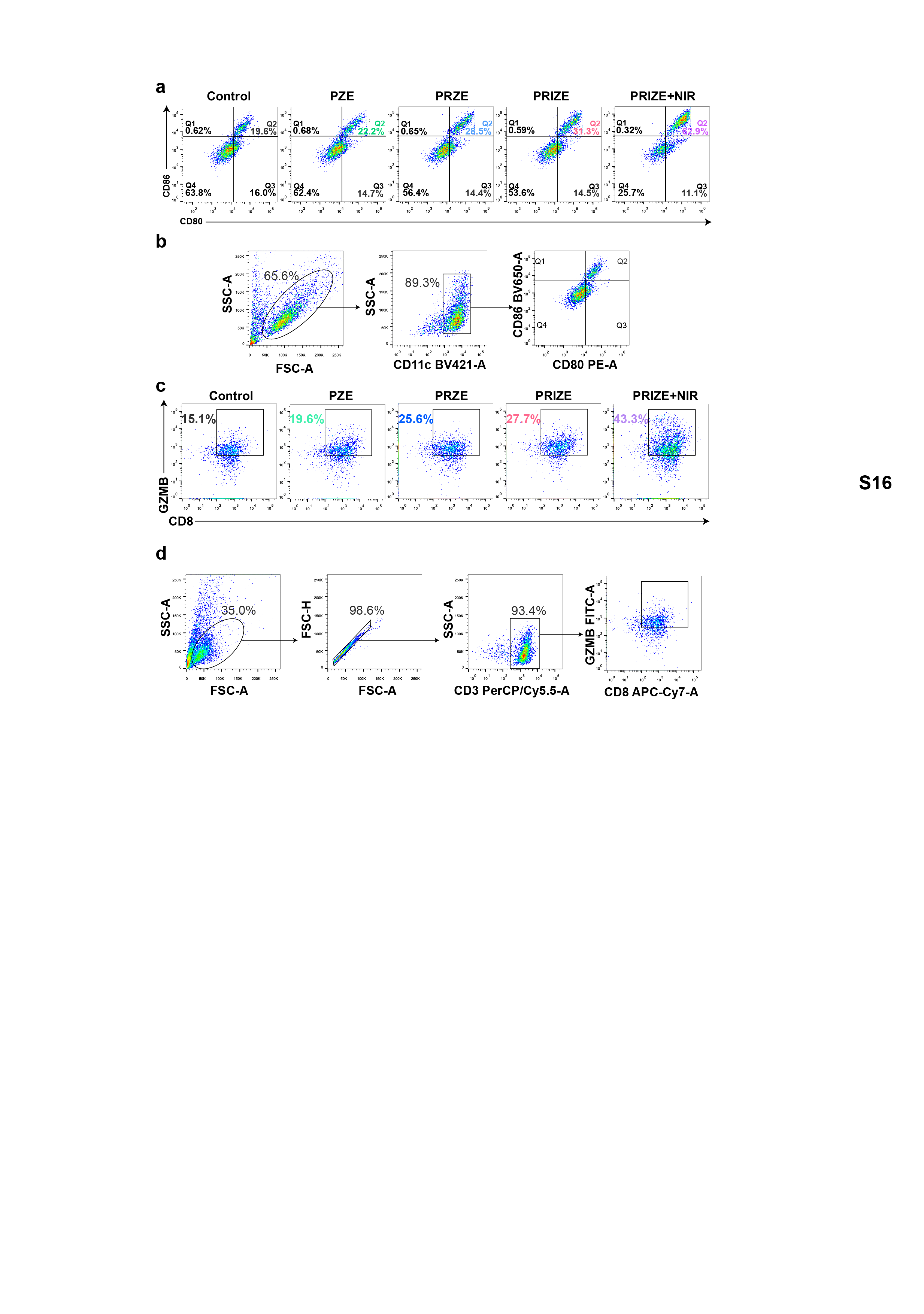
**

**Figure S14.** a) Representative flow cytometry analysis of CD80, CD86, positive cells (gated on CD11c^+^ cells) of BMDCs after different treatments. b) The gating strategy for flow cytometry analysis of CD11c^+^ CD80^+^/CD86^+^ DCs in BMDCs. c) Typical flow cytometry plots of activated CD8^+^ T cells following co-culture with the BMDCs. d) The gating strategy for flow cytometry analysis of CD3^+^ CD8^+^ GZMB^+^ T cells.


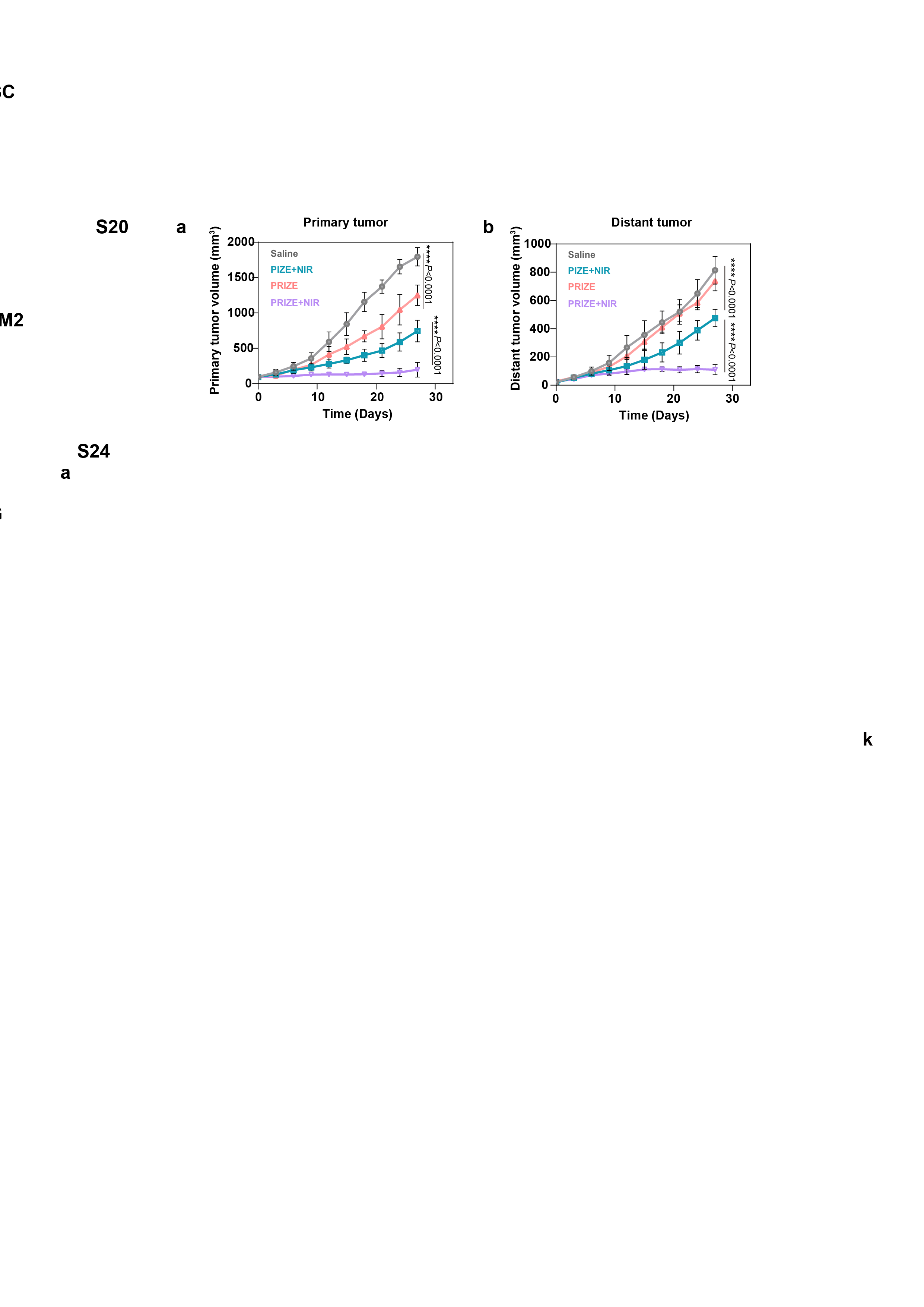


**Figure S15.** a) Tumor growth curves of the primary tumors and distant tumors (n = 8 biologically independent samples). Statistical analyses were done using one-way ANOVA with Tukey’s multiple comparisons test and correction. **P* < 0.05, ***P* < 0.01, ****P* < 0.001, *****P* < 0.0001, ns, not significant. Data are presented as mean ± SD.


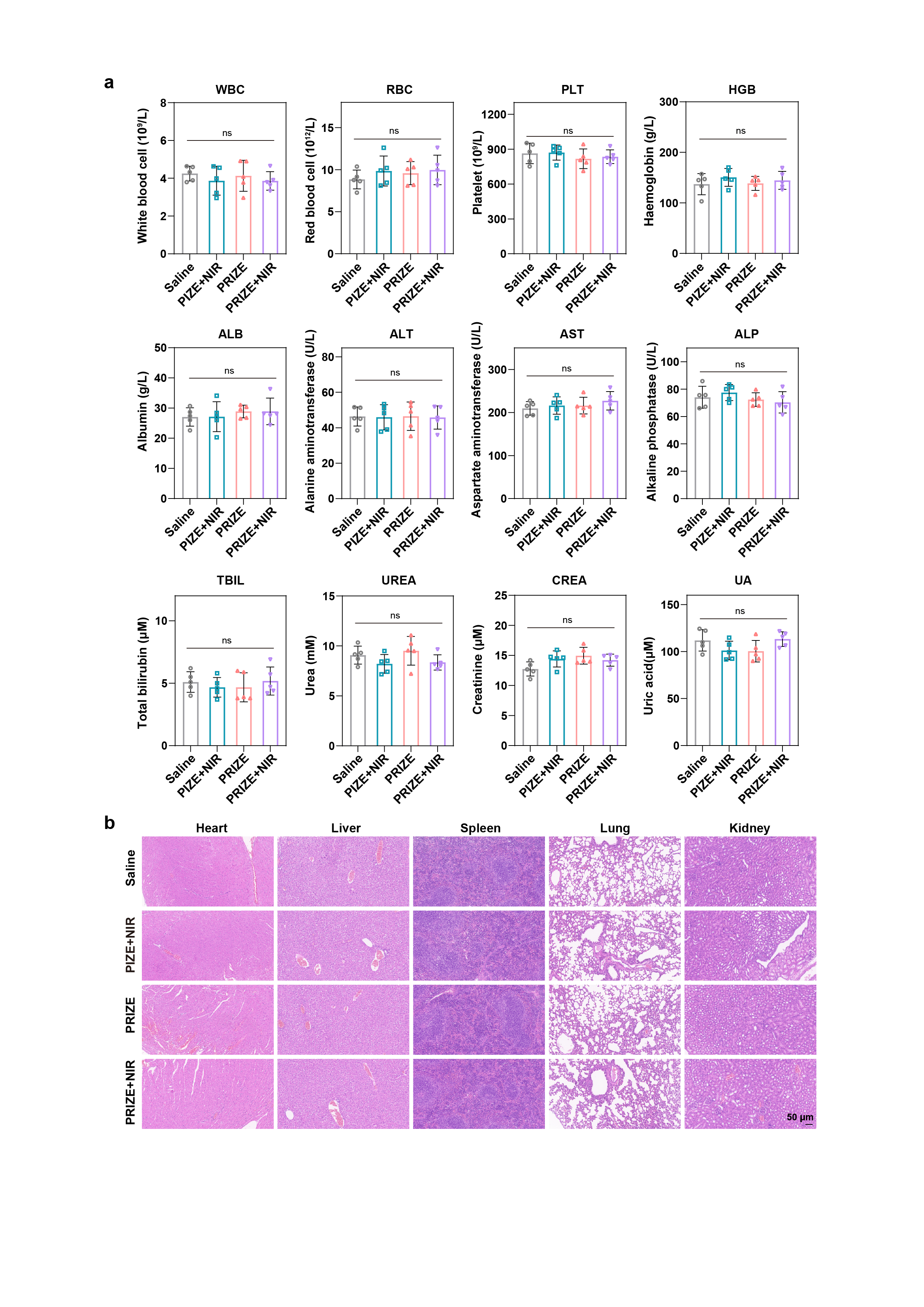


**Figure S16.** a) Blood parameter analysis and blood biochemistry analysis of mice with different treatments (n = 5 biologically independent samples). NIR irradiation at 43℃ for 5 min. WBC, white blood cell; RBC, red blood cell; PLT, platelet; HGB, hemoglobin; ALB, albumin; ALT, plasma alanine aminotransferase; AST, aspartate aminotransferase; ALP, alkaline phosphatase; TBIL, total bilirubin; UREA, urea; CREA, creatinine; UA, uric acid. b) H&E staining of major organs including heart, liver, spleen, lung, and kidney collected from mice treated with saline, PIZE+NIR, PRIZE, and PRIZE+NIR. **P* < 0.05, ***P* < 0.01, ****P* < 0.001, *****P* < 0.0001, ns, not significant. Data are presented as mean ± SD.


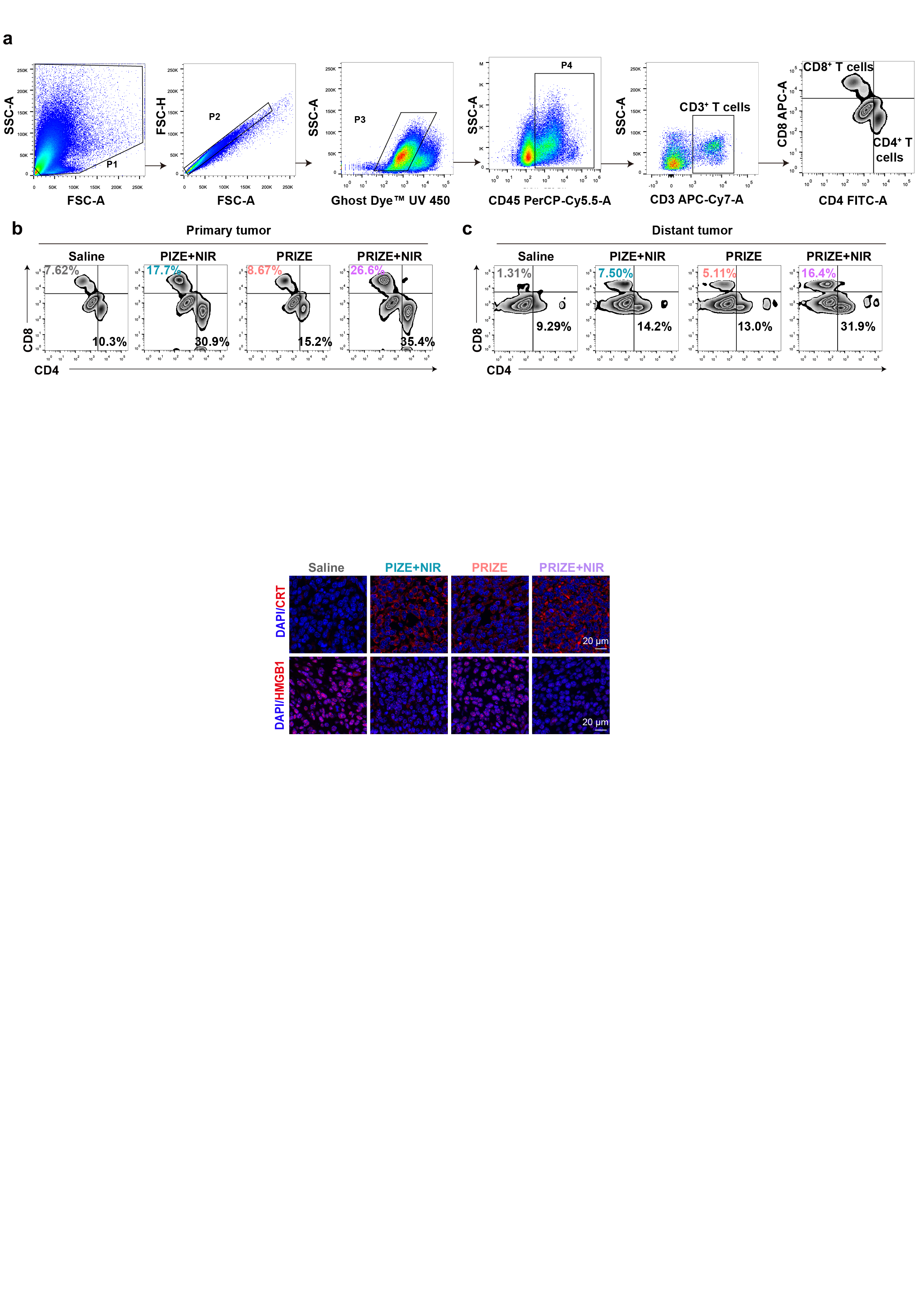


**Figure S17.** Representative immunofluorescence images showing the expression of CRT, and HMGB1 in primary tumors treated by the indicated formulations in combination with NIR.


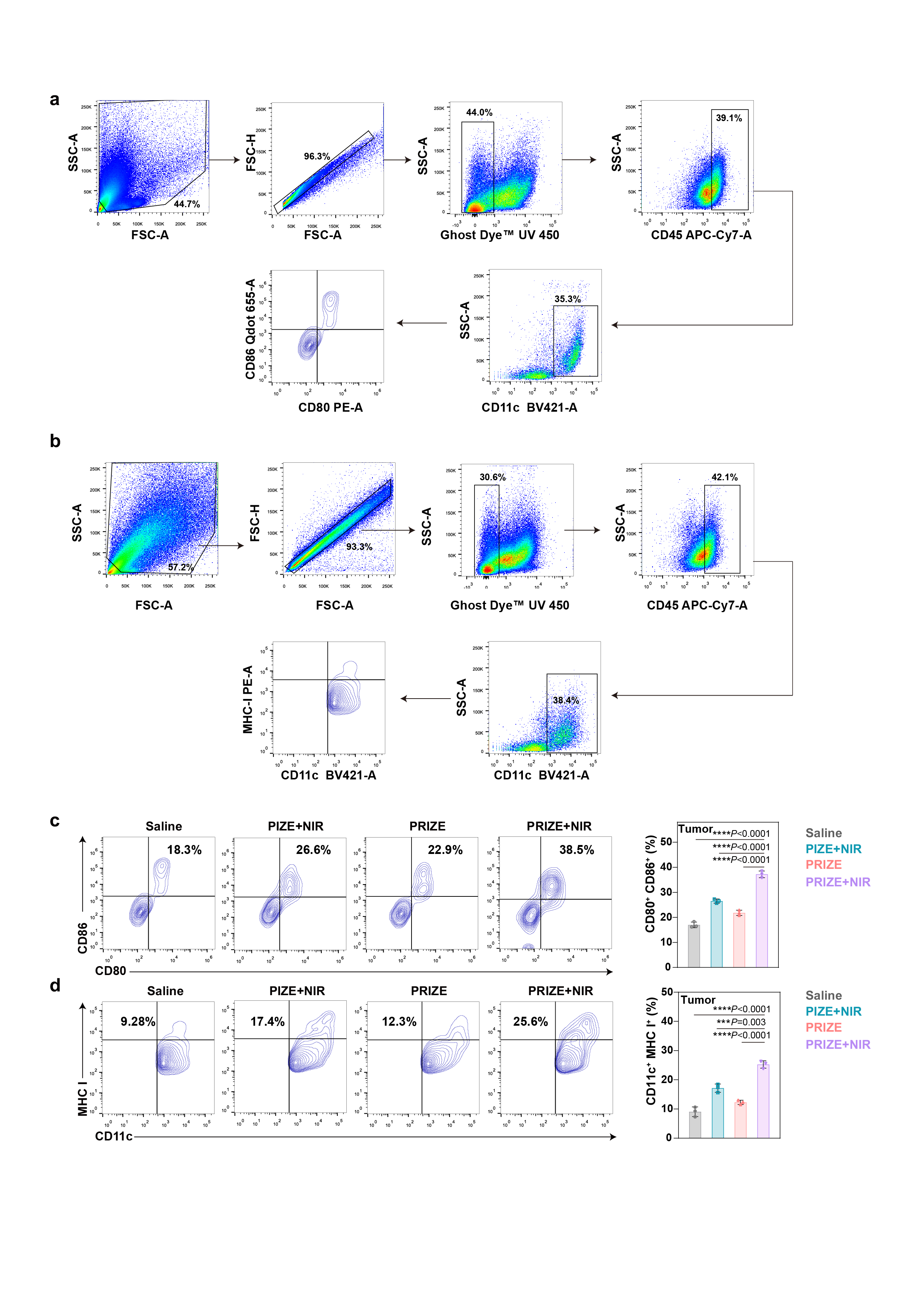


**Figure S18.** a-b) The gating strategy for flow cytometry analysis of DCs in the tumor. c) Representative flow cytometry plots (left) and quantitative analysis (right) of CD80^+^ CD86^+^ DCs in tumor after different treatments. d) Representative flow cytometry plots (left) and quantitative analysis (right) of CD11c^+^ MHC I^+^ DCs in tumor after different treatments. **P* < 0.05, ***P* < 0.01, ****P* < 0.001, *****P* < 0.0001, ns, not significant. Data are presented as mean ± SD.


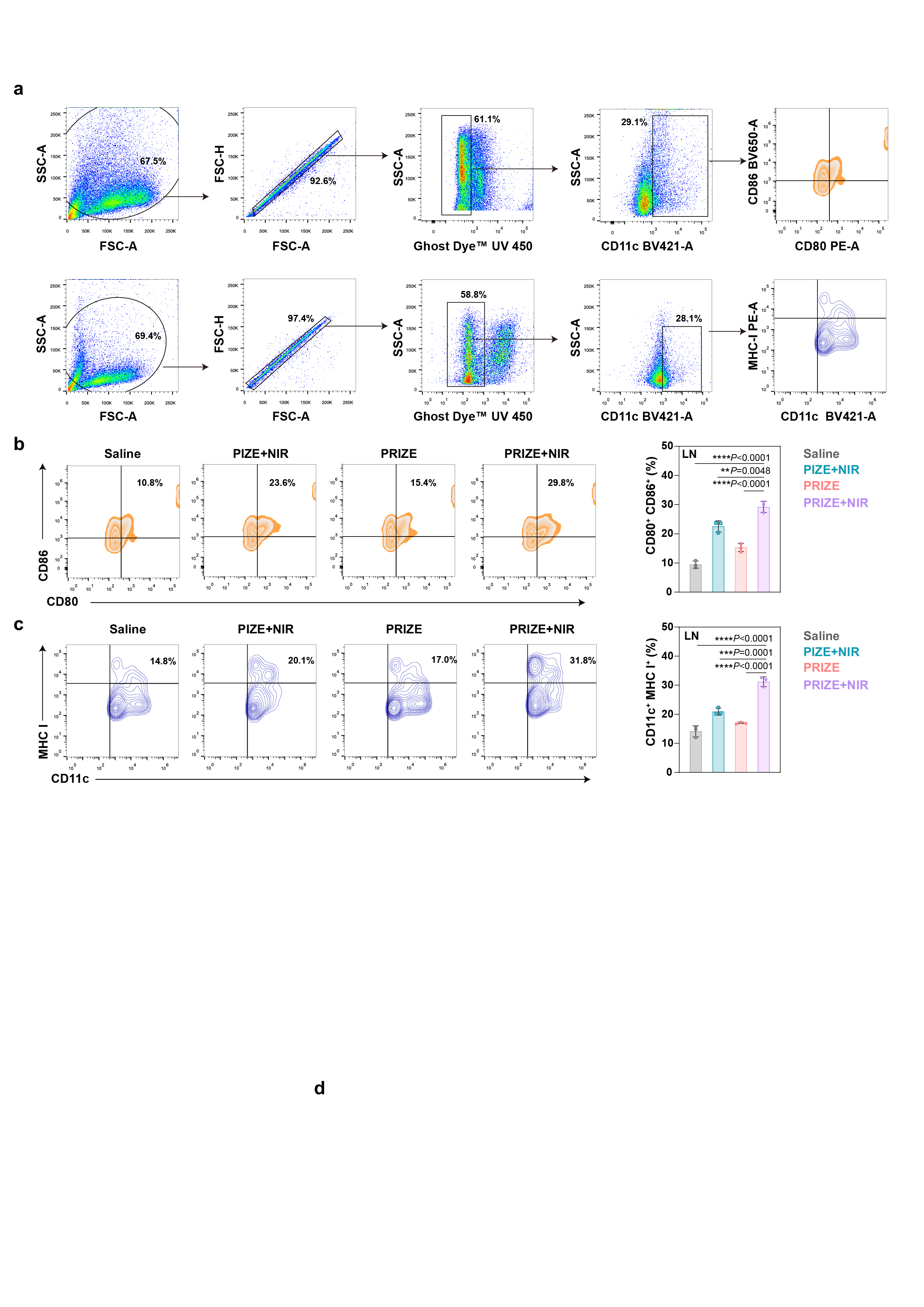


**Figure S19.** a) The gating strategy for flow cytometry analysis of DCs in LNs. b) Representative flow cytometry plots (left) and quantitative analysis (right) of CD80^+^ CD86^+^ DCs in LNs after different treatments. c) Representative flow cytometry plots (left) and quantitative analysis (right) of CD11c^+^ MHC I^+^ DCs in tumor after different treatments. **P* < 0.05, ***P* < 0.01, ****P* < 0.001, *****P* < 0.0001, ns, not significant. Data are presented as mean ± SD.


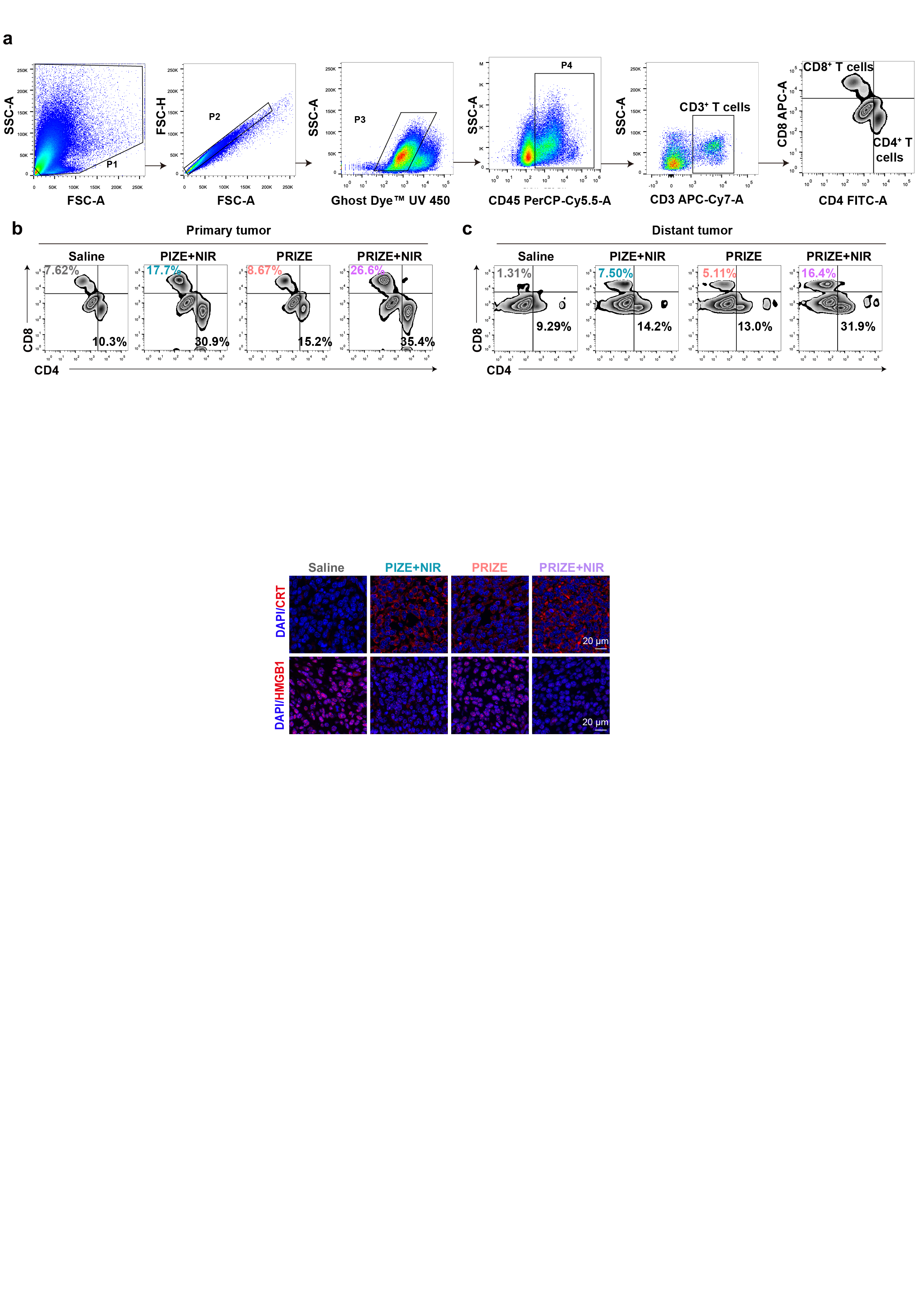


**Figure S20.** a) Representative gating strategy for flow cytometry analysis CD8^+^ T cells (gated on CD3^+^ cells) in tumors. b-c) Representative flow cytometry analysis of infiltrated CD8^+^ T cells (gated on CD3^+^ cells) in the primary tumor (b) and distant tumor (c) of mice in different groups.


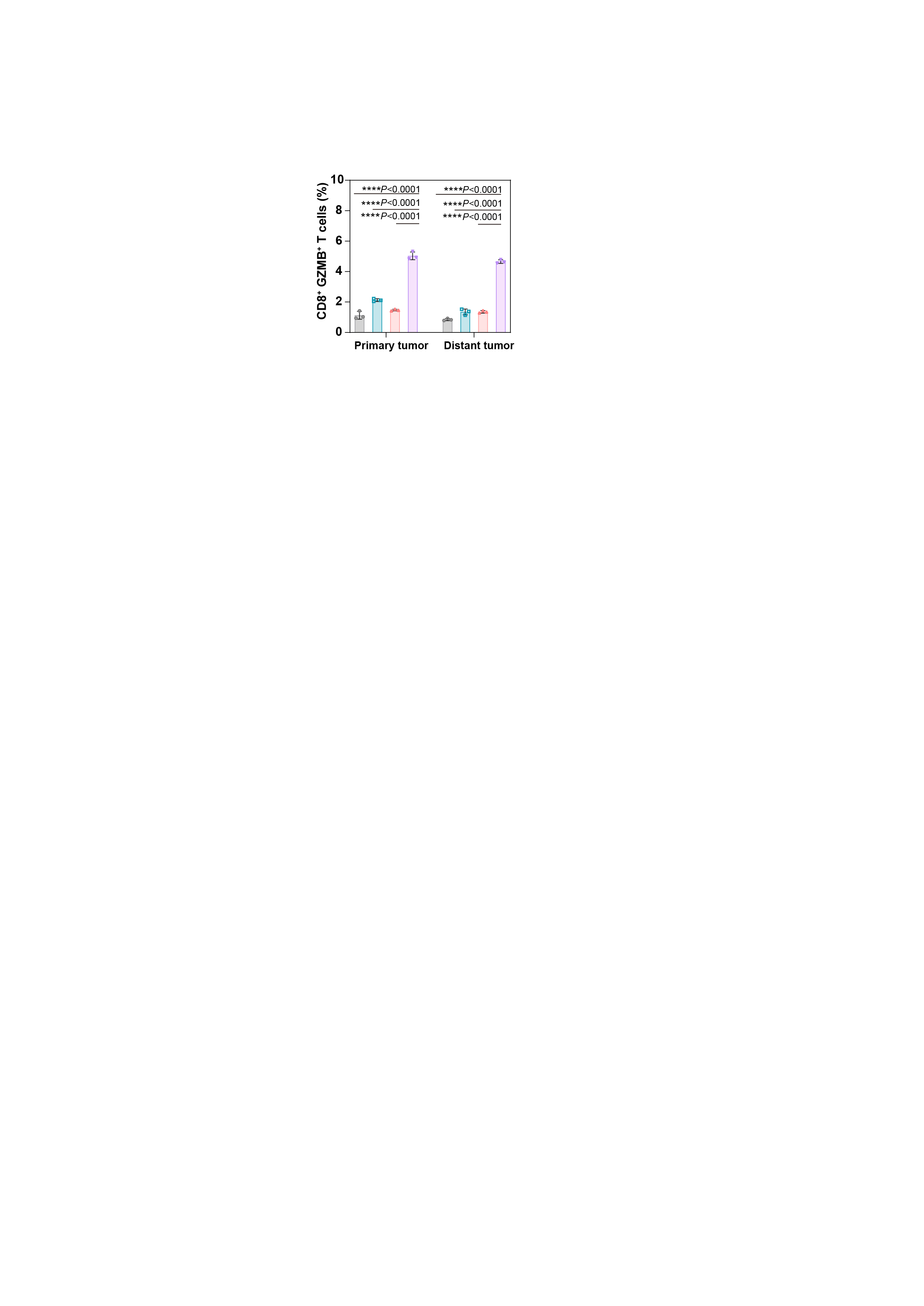


**Figure S21.** Quantitative analysis of CD8^+^ GZMB^+^ T cells in primary and distant tumors.


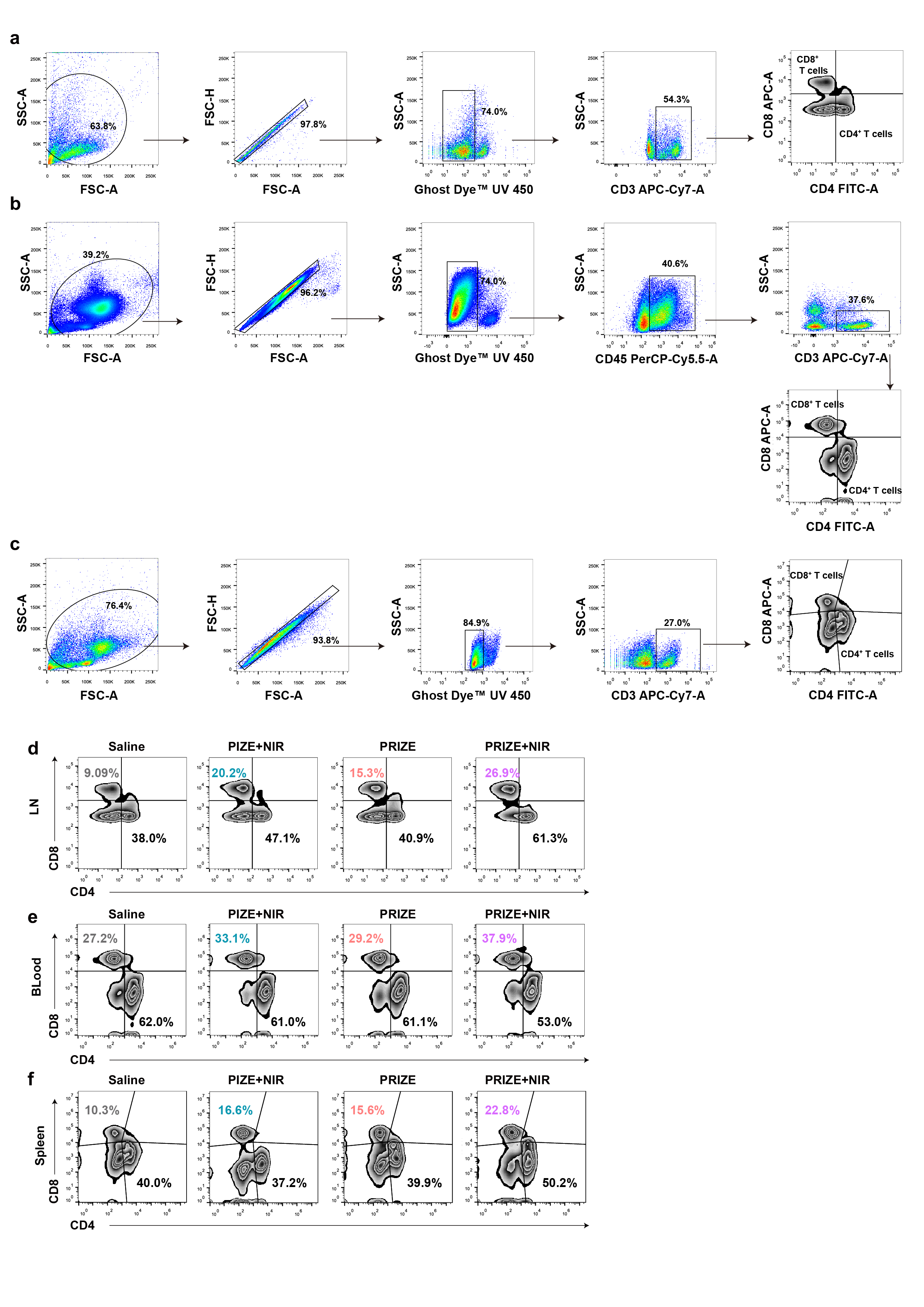


**Figure S22.** a**-**c) The gating strategy for flow cytometry analysis of CD3^+^ CD8^+^ T cells in LNs (a), blood (b), spleens (c). d**-**f) Representative flow cytometry analysis of infiltrated CD3^+^ CD8^+^ T cells in LNs (d), blood (e), spleens (f) of mice in different groups.


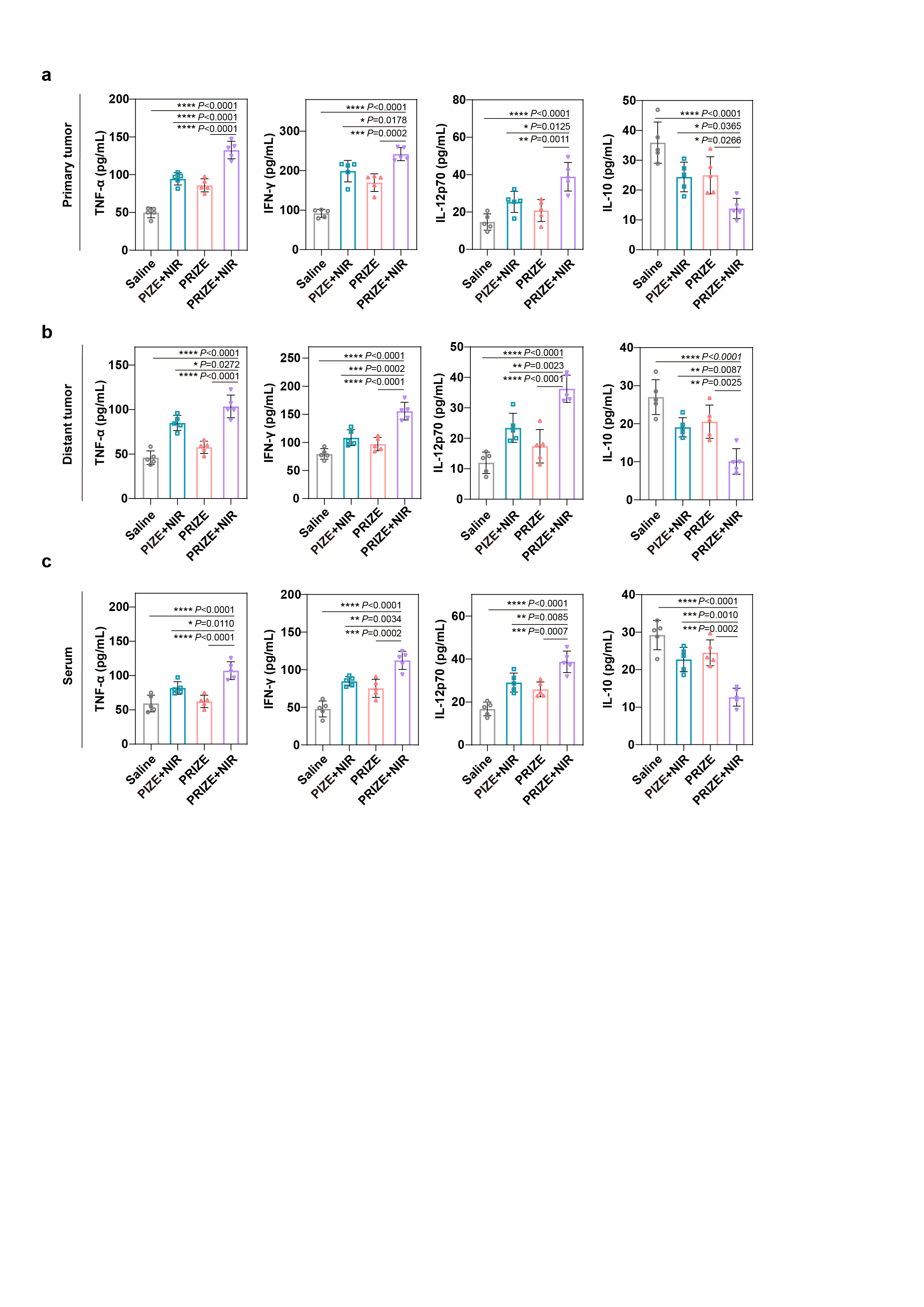


**Figure S23.** a-c) Quantitative ELISA analysis of IFN-γ, TNF-α, IL-12p70 and IL-10 cytokine levels in (a) primary tumor, (b) distant tumor and (c) serum of mice (n = 5 biologically independent experiments). Statistical analyses were done using one-way ANOVA with Tukey’s multiple comparisons test and correction. **P* < 0.05, ***P* < 0.01, ****P* < 0.001, *****P* < 0.0001, ns, not significant. Data are presented as mean ± SD.


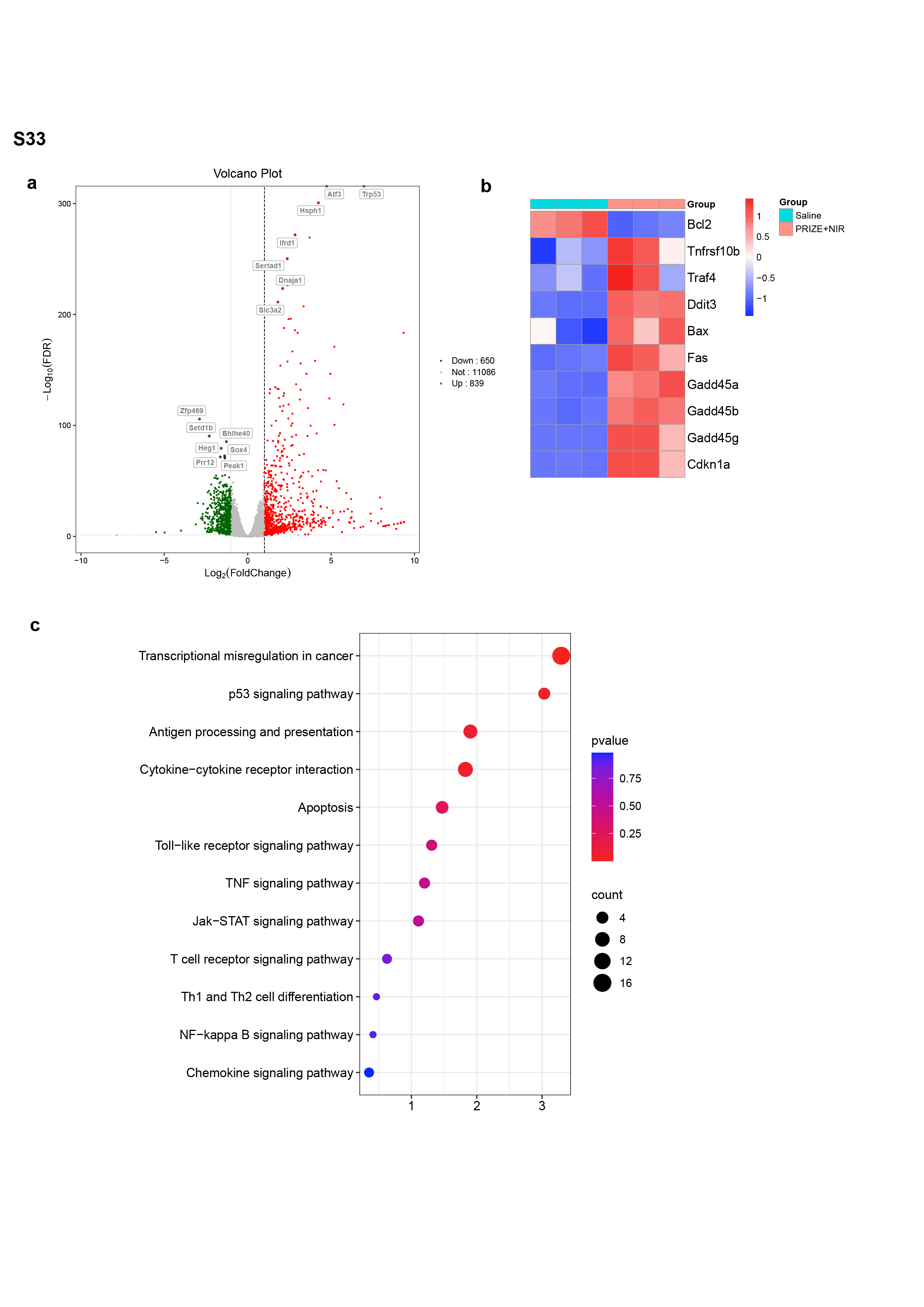


**Figure S24.** a) Volcanic map of differentially expressed genes (DEGs). The up-regulated genes are represented by red dots and the down-regulated genes by green dots. b) Heat map showing the normalized expression of differentially expressed apoptosis genes associated with P53. c) KEGG enrichment analysis of the DEGs between saline and PRIZE+NIR-treated tumors (analysis was performed by one-sided hypergeometric distribution test followed by false discovery rate correction).


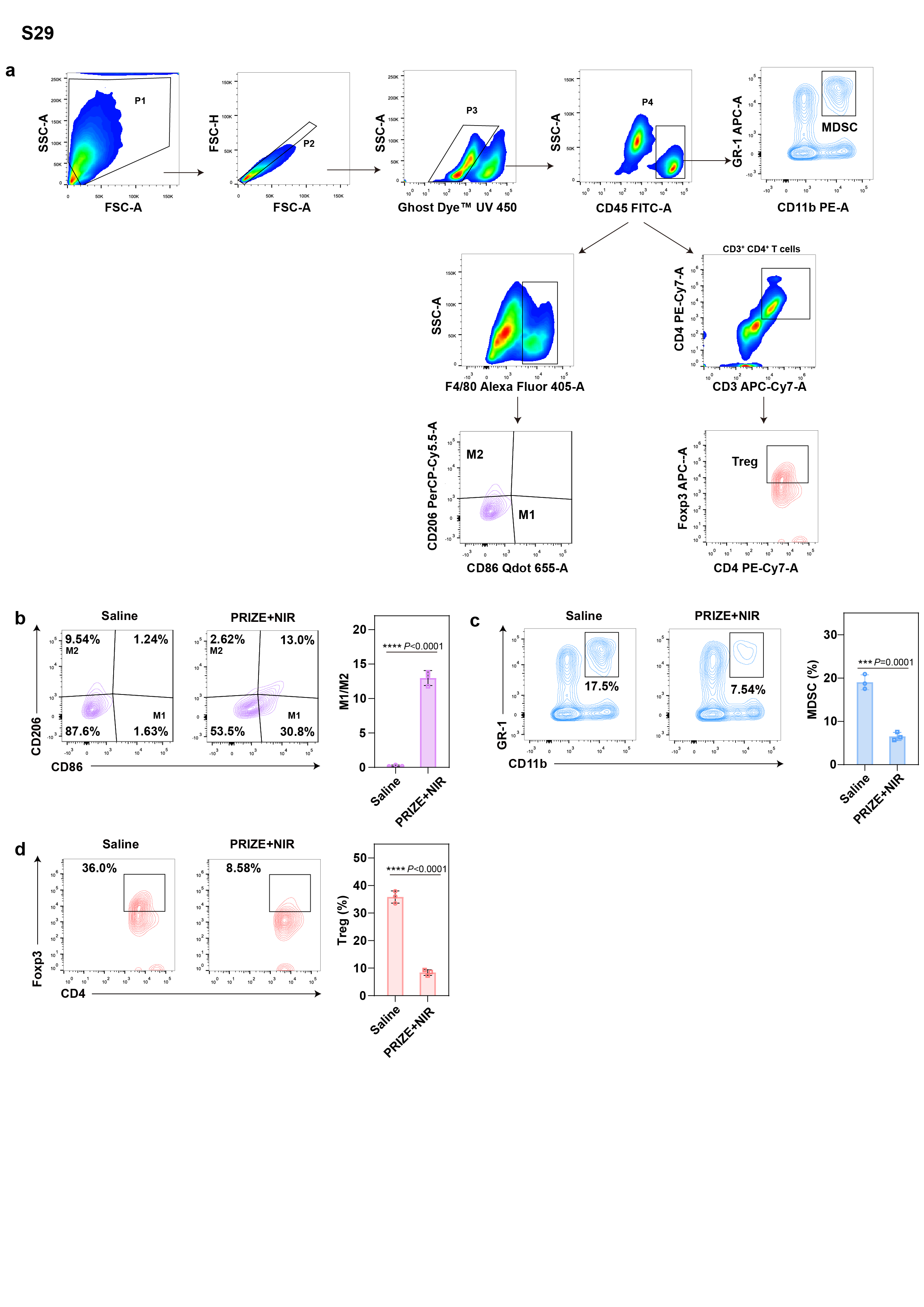


**Figure S25.** a) Representative gating strategies for flow cytometry analysis of MDSCs, M1 TAMs, M2 TAMs, and Tregs in tumor. b) Representative flow cytometry analysis of M1 TAMs (F4/80^+^ CD206^-^ CD86^+^), M2 TAMs (F4/80^+^ CD206^+^ CD86^+^ and quantitative flow cytometry data on the ratio of tumor associated macrophages M1 to M2. c-d) Representative flow cytometry analysis and quantitative flow cytometry data of MDSCs (CD11b^+^ Gr-1^+^) (c) and Treg (CD4^+^ Foxp3^+^) (d) of primary tumor of mice in different groups (n = 3 biologically independent experiments).


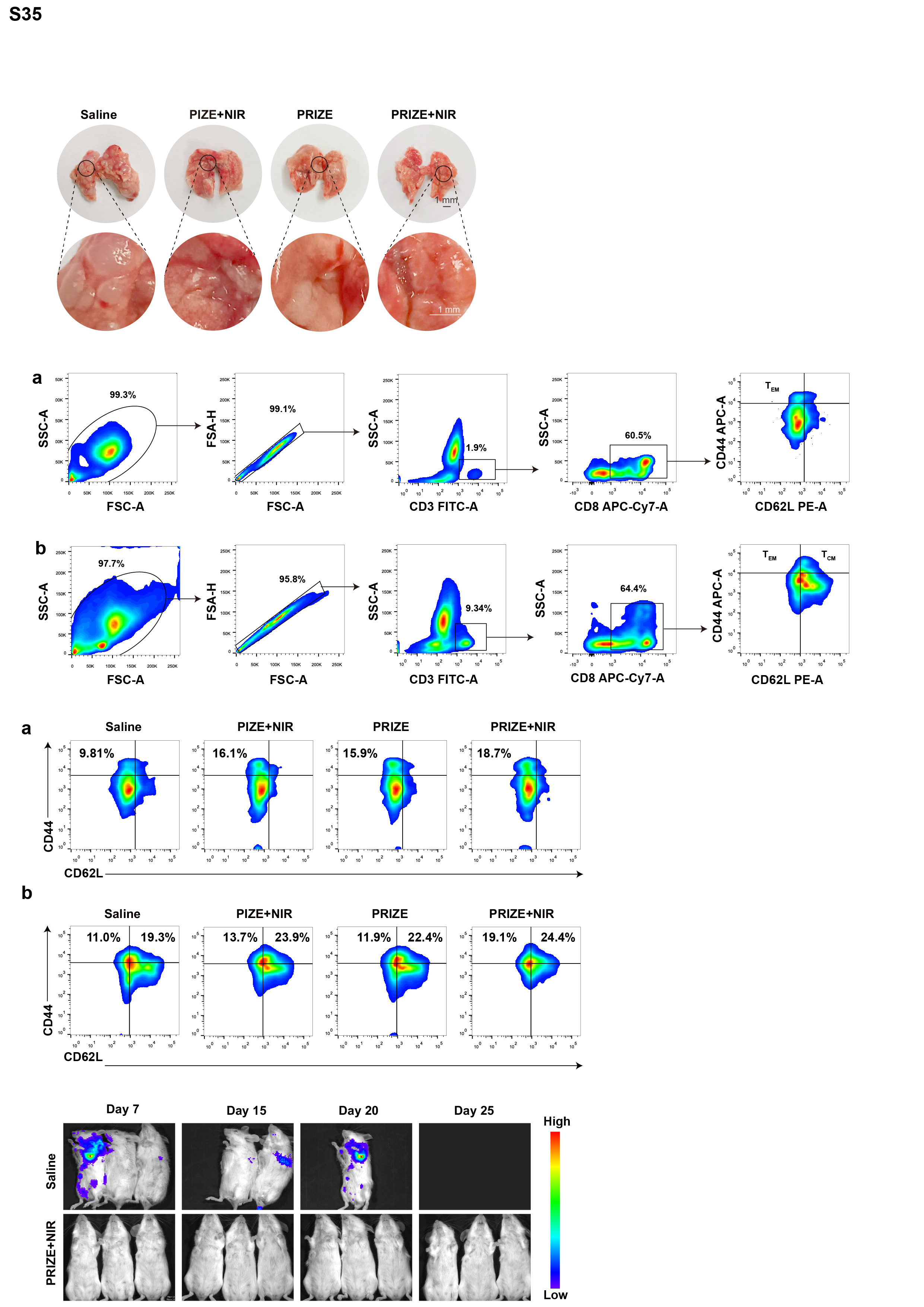


**Figure S26.** Representative photographs of lung tissues isolated at the end of the study.


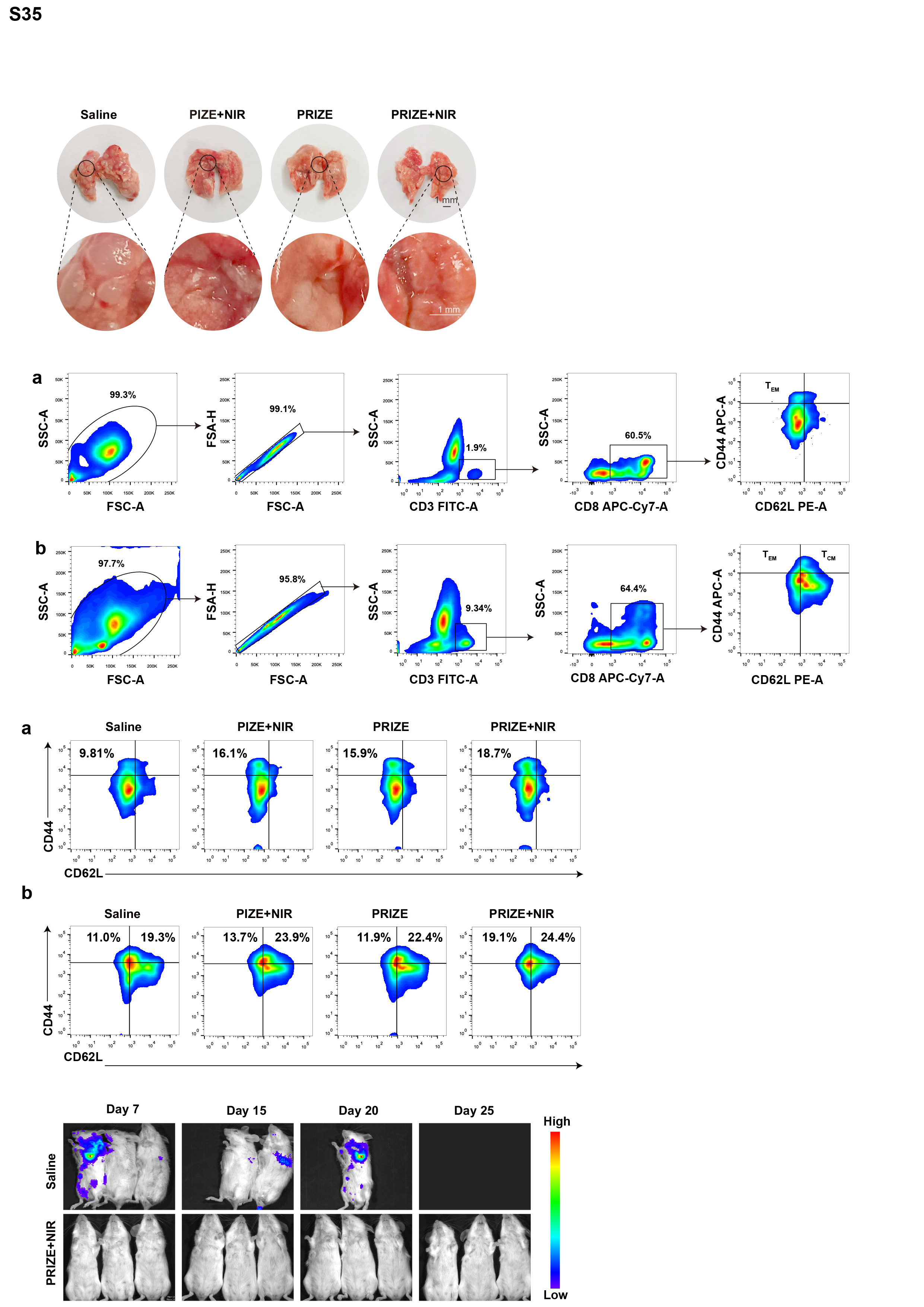


**Figure S27.** Bioluminescence images of saline and PRIZE+NIR-treated mice after intravenous injection of Luc-4T1 cells.


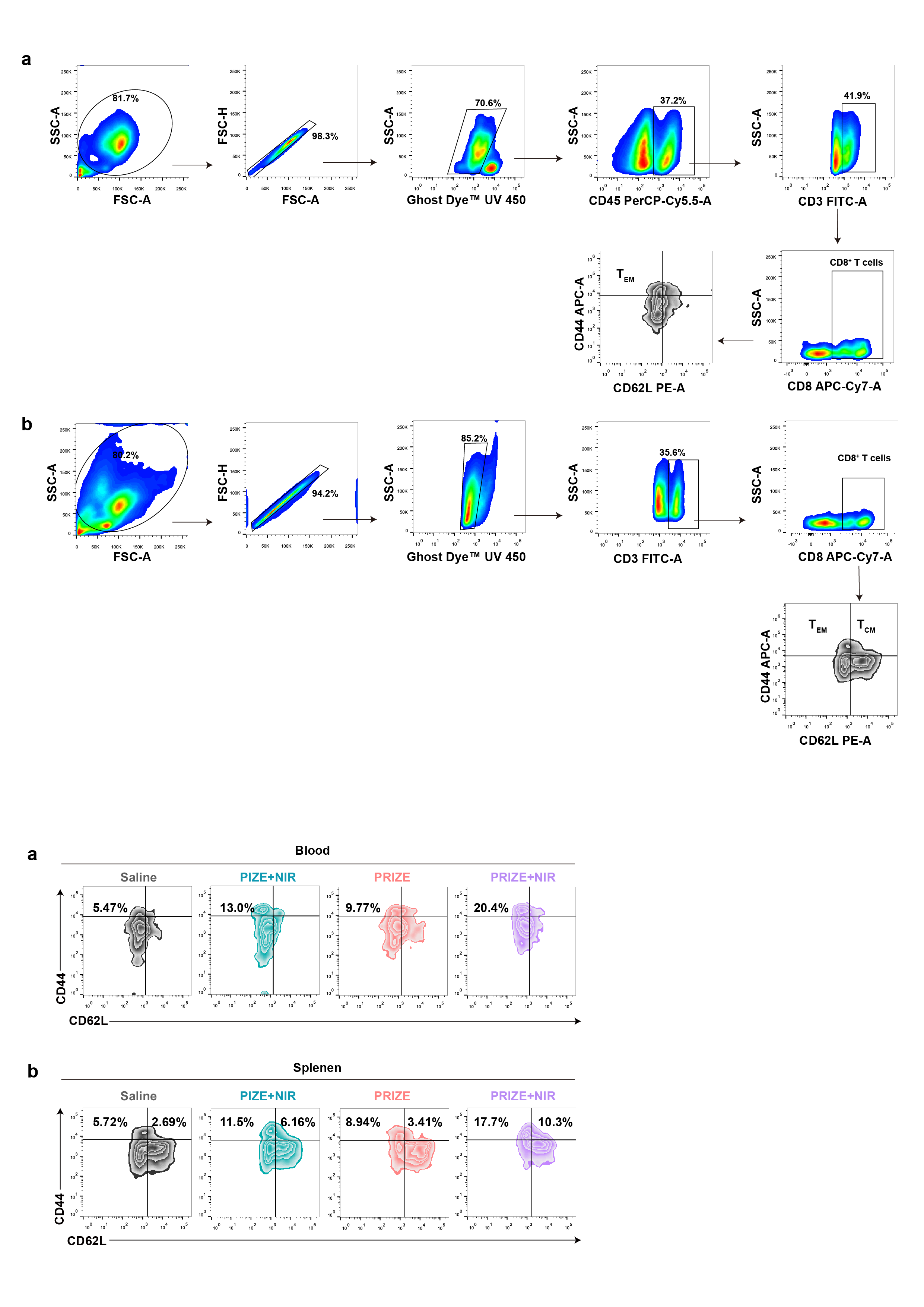


**Figure S28.** a) The gating strategy for flow cytometry analysis of T_EM_ in blood. b) The gating strategy for flow cytometry analysis of T_EM_ and T_CM_ in spleens. The gating strategy for T_EM_ (CD8^+^ CD44^+^ CD62L^−^) in blood, T_EM_ (CD8^+^ CD44^+^ CD62L^−^) and T_CM_ (CD8^+^ CD44^+^ CD62L^+^) in spleens used in Figure S29a-b, Supporting Information.


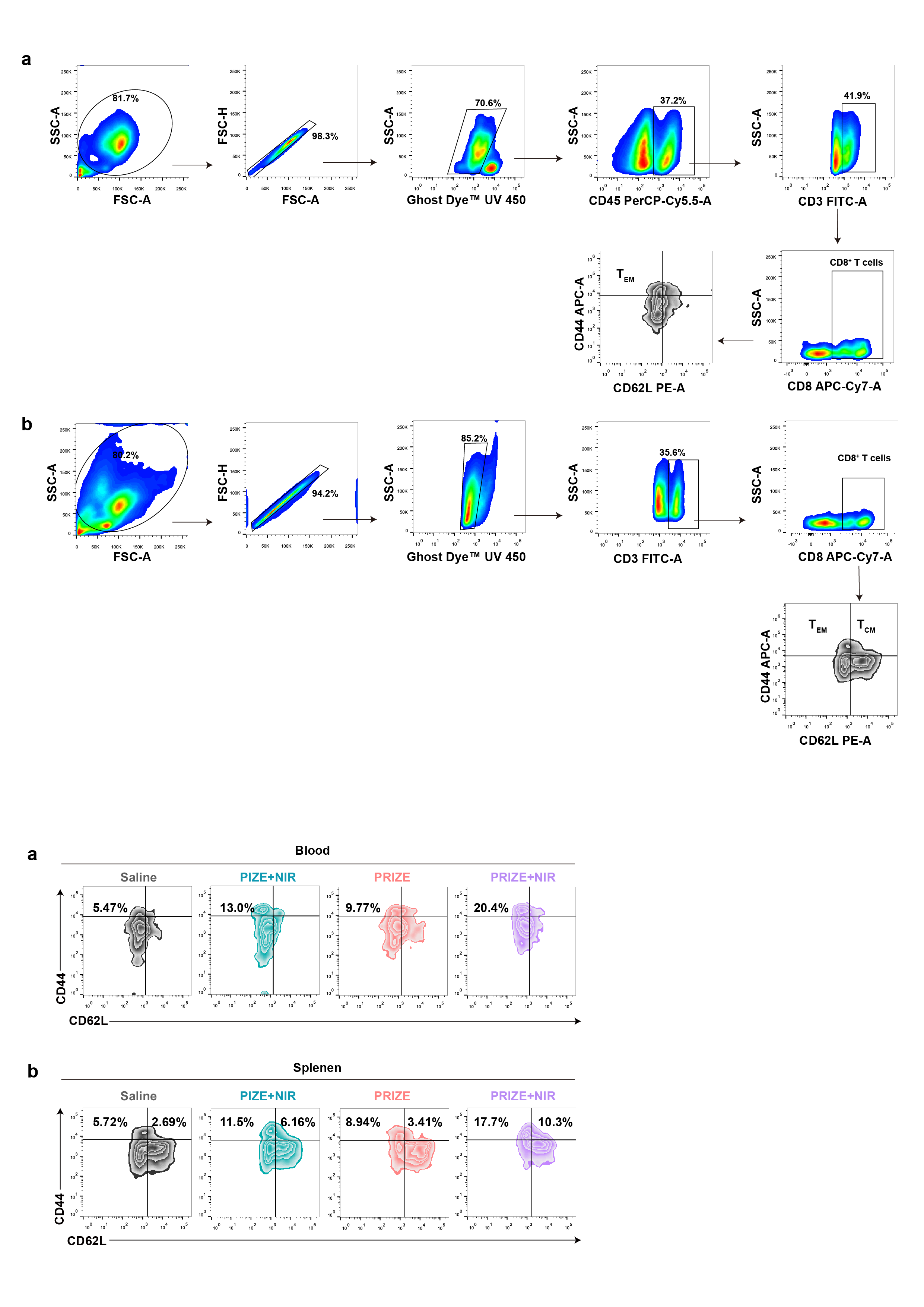


**Figure S29.** a) Representative flow cytometry analysis of T_EM_ (CD8^+^ CD44^+^ CD62L^−^) in blood collected from tumor recurrence in 4T1 tumor model with the various treatments. b) Representative flow cytometry analysis of T_EM_ (CD8^+^ CD44^+^ CD62L^−^) and T_CM_ (CD8^+^ CD44^+^ CD62L^+^) in spleens collected from tumor recurrence in 4T1 tumor model with the various treatments.


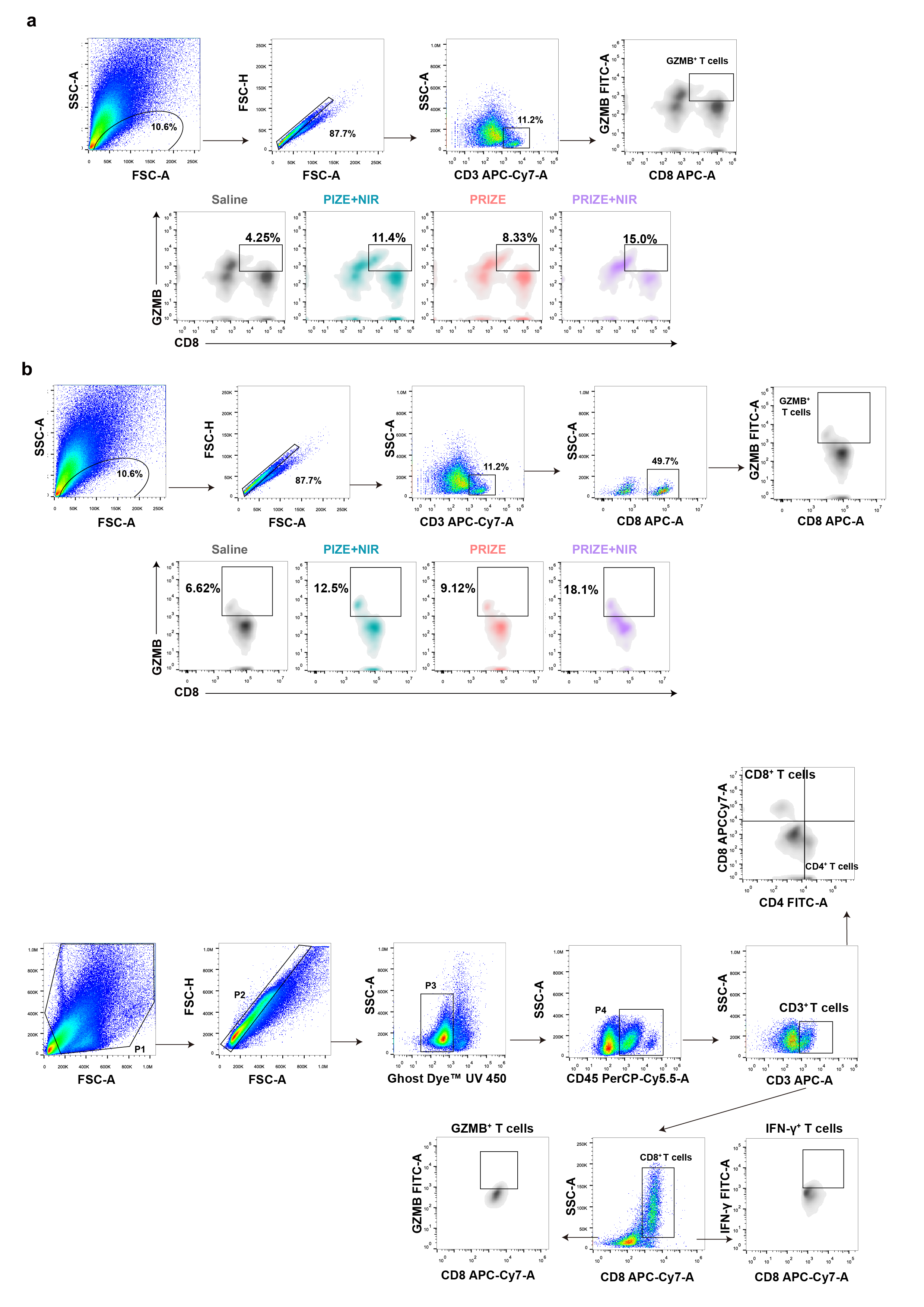


**Figure S30.** Representative gating strategies for analyzing infiltrated T cell in MC38 tumors.

**Table S1:** **mRNA sequences used in this study**

| ***p53* mRNA** | ATGACTGCCATGGAGGAGTCACAGTCGGATATCAGCCTCGAGCTCCCTCTGAGCCAGGAGACATTTTCAGGCTTATGGAAACTACTTCCTCCAGAAGATATCCTGCCATCACCTCACTGCATGGACGATCTGTTGCTGCCCCAGGATGTTGAGGAGTTTTTTGAAGGCCCAAGTGAAGCCCTCCGAGTGTCAGGAGCTCCTGCAGCACAGGACCCTGTCACCGAGACCCCTGGGCCAGTGGCCCCTGCCCCAGCCACTCCATGGCCCCTGTCATCTTTTGTCCCTTCTCAAAAAACTTACCAGGGCAACTATGGCTTCCACCTGGGCTTCCTGCAGTCTGGGACAGCCAAGTCTGTTATGTGCACGTACTCTCCTCCCCTCAATAAGCTATTCTGCCAGCTGGCGAAGACGTGCCCTGTGCAGTTGTGGGTCAGCGCCACACCTCCAGCTGGGAGCCGTGTCCGCGCCATGGCCATCTACAAGAAGTCACAGCACATGACGGAGGTCGTGAGACGCTGCCCCCACCATGAGCGCTGCTCCGATGGTGATGGCCTGGCTCCTCCCCAGCATCTTATCCGGGTGGAAGGAAATTTGTATCCCGAGTATCTGGAAGACAGGCAGACTTTTCGCCACAGCGTGGTGGTACCTTATGAGCCACCCGAGGCCGGCTCTGAGTATACCACCATCCACTACAAGTACATGTGTAATAGCTCCTGCATGGGGGGCATGAACCGCCGACCTATCCTTACCATCATCACACTGGAAGACTCCAGTGGGAACCTTCTGGGACGGGACAGCTTTGAGGTTCGTGTTTGTGCCTGCCCTGGGAGAGACCGCCGTACAGAAGAAGAAAATTTCCGCAAAAAGGAAGTCCTTTGCCCTGAACTGCCCCCAGGGAGCGCAAAGAGAGCGCTGCCCACCTGCACAAGCGCCTCTCCCCCGCAAAAGAAAAAACCACTTGATGGAGAGTATTTCACCCTCAAGATCCGCGGGCGTAAACGCTTCGAGATGTTCCGGGAGCTGAATGAGGCCTTAGAGTTAAAGGATGCCCATGCTACAGAGGAGTCTGGAGACAGCAGGGCTCACTCCAGCCTCCAGCCTAGAGCCTTCCAAGCCTTGATCAAGGAGGAAAGCCCAAACTGCTAG |
| --- | --- |

**Table S2:** **Primers used for qRT-PCR**

| **Name** | **Sequence (5’~3’)** |
| --- | --- |
| *B2M* (mouse) | Forward: TTCTGGTGCTTGTCTCACTGA  Reverse: CAGTATGTTCGGCTTCCCATTC |
| *Tap1* (mouse) | Forward: GGACTTGCCTTGTTCCGAGAG  Reverse: GCTGCCACATAACTGATAGCGA |
| *Erap1* (mouse) | Forward: TAATGGAGACTCATTCCCTTGGA  Reverse: AAAGTCAGAGTGCTGAGGTTTG |
| *H-2K1* (mouse) | Forward: ACCAAACACAAGTGGGAGCA  Reverse: CATGGGCCTTTGGGGAATCT |
| *PD-L1* (mouse) | Forward: ACTTGTACGTGGTGGAGTATGGC  Reverse: TGCTCATCTTCCTTTTCCCAGTA |
| *Actb* (mouse) | Forward: GTCGTACCACAGGCATTGTGATGG  Reverse: GCAATGCCTGGGTACATGGTGG |
